# Dielectric stacking-engineered scalable reconfigurable transistor platform for adaptive logic circuits

**DOI:** 10.1038/s41467-026-75864-2

**Published:** 2026-07-24

**Authors:** Pengfei Zhu, Chi Zhang, Jingbo Yang, You-Wei Guo, Shida Huo, Enxiu Wu, Fei Wang, Che-Yi Lin, Jyun-Hong Chen, Hongling Chu, Zhaorui Liu, Song Zhao, Jun Li, Mengjiao Li, Yen-Fu Lin, Jianhua Zhang

**Affiliations:** 1 School of Microelectronics, Shanghai University, Shanghai, China; 2 Department of Physics, National Chung Hsing University, Taichung, Taiwan; 3 Graduate Program in Semiconductor and Green Technology, Academy of Circular Economy, National Chung Hsing University, Nantou City, Taiwan; 4 State Key Laboratory of Precision Measurement Technology and Instruments, School of Precision Instruments and Opto-electronics Engineering, Tianjin University, Tianjin, China; 5 Department of Applied Physics, Tunghai University, Taichung, Taiwan; 6 National Applied Research Laboratories, Taiwan Semiconductor Research Institute, Hsinchu, Taiwan; 7 Shanghai Key Laboratory of Automotive Intelligent Connected Interaction Chips and Systems, Shanghai, China

**Keywords:** Electronic devices, Electronic devices, Electronic properties and materials

## Abstract

Although reconfigurable van der Waals devices featuring flexible logic transformation offer a promising strategy toward adaptable architectures to accommodate diverse computational demands, reliable polarity control and scalable integration remain challenging. Here, we demonstrate a reconfigurable field-effect transistor based on the scalable dielectric oxide-van der Waals quasi-floating-gate configuration, enabling nonvolatile polarity switching and multi-state programmability. Charge trapping engineering in an atomic-layer Al_2_O_3_/HfO_2_/Al_2_O_3_ dielectric stack achieves performance with nonvolatile conductance update (>6-bits for 1000 s), robust endurance (>3 × 10^5^ cycles), and well-balanced electron/hole transport (current mismatch ratio ~ 1%). TCAD simulation and surface potential analysis reveal oxygen vacancies-dominated polarity switching dynamics. Using a silicon-compatible top-gate dielectric process and complementary design, diverse logic gates—including eight Boolean operations and seamless AND-OR-Invert/OR-AND-Invert transformations—are accommodated into compact reconfigurable logic-in-memory circuits. These transistors also simplify ternary content-addressable memory design, underscoring their potential for efficient logic-in-memory computing.

## Abstract (web summary)

Reconfigurable devices offer a promising strategy toward adaptable architectures to accommodate diverse computational demands. Zhu et al. report a van der Waals transistor with nonvolatile polarity control and multi-state memory, enabling logic-in memory, Boolean functions, and ternary content-addressable memory.

## Introduction

The rapid advancement of artificial intelligence (AI) and the Internet of Things (IoT) imposes stringent demands on computational architectures, including high computing power, energy-efficient data transfer, and reconfigurable hardware resources^1–4^. In-memory computing architectures built on emerging memory technologies offer a promising route to mitigate the latency and energy overheads inherent in conventional computing systems^5–10^. Yet, the fixed electrical properties of silicon-based CMOS devices—dictated by doping profiles and layout-dependent effects—limit the dynamic allocation of hardware resources, posing a fundamental challenge in meeting diverse computational needs^11–14^.

Reconfigurable field-effect transistors (RFETs) attract considerable interest for their ability to dynamically modulate energy bands and channel polarity via electrostatic gating^15–17^. This flexible polarity control enables logic circuits with runtime reconfigurability and simplifies the design of ternary content-addressable memories (TCAMs), which are essential for high-speed search operations in memory-intensive applications^18–24^. Early RFET implementations using carbon nanotubes demonstrate polarity tuning via fixed bottom-gate voltages to shift channel energy bands^25,26^. However, these electrostatic devices are volatile and require constant gate bias, leading to static power dissipation and thermal issues^16,27^.

The emergence of two-dimensional van der Waals (vdW) materials opens pathways to nonvolatile reconfigurability^28–30^. For instance, optical gating and ferroelectric polarization using materials like AlScN or HZO—grown via atomic layer deposition (ALD)—are used to achieve improved device uniformity and multi-level storage^31–34^. Despite this progress, reliable polarity control and scalable integration in nanoscale vdW RFETs remain challenging. Top-gate integration, in particular, is often hampered by thermal budget constraints, poor lithographic compatibility, and unwanted dielectric-induced doping^35–39^. Consequently, most vdW RFETs still rely on bottom-gate geometries, which limit their applicability in 3D integrated circuits. Although mechanically exfoliated boron nitride (BN) can be used in top-gate configurations, such approaches lack scalability and impede circuit-level control^16,40^. Achieving highly reliable multi-state programmability and scalable reconfigurable device array calls for innovation in both electrical modulation strategies and gating control engineering.

Inspired by the multi-level storage behavior of floating-gate memories, we develop a scalable vdW RFET based on an Al₂O₃/HfO₂/Al₂O₃ (AHA) multi-dielectric quasi-floating-gate structure (AHA-RFET). Electrical charge trapping in the AHA stack induces a vertical field that reversibly modulates channel conduction, enabling 64 distinct states (6-bit) with retention exceeding 1000 s. A partially top-gated design introduces a stair-like band profile in the channel, yielding closely matched N- and P-type transfer characteristics in terms of current, threshold voltage, and subthreshold swing. Technology Computer-Aided Design (TCAD) simulations of electric field and surface potential evolution further clarify the role of the AHA stack and top-gate geometry in enabling multi-state programmability and polarity reconfiguration. By combining high-quality ALD top-gate dielectrics with a low thermal budget (≤200 °C), we demonstrate AHA-RFETs in logic circuits, including inverters with a voltage gain of 51, a four-transistor (4T) logic gate implementing eight Boolean functions (NOR, NAND, OR, AND, IMP, NIMP, RIMP, RNIMP), and eight-transistor (8T) AND-OR-Invert (AOI) and OR-AND-Invert (OAI) circuits. Moreover, we present a compact TCAM cell based on AHA-RFETs that reduces area footprint by 87.5% while supporting parallel search operations. These results highlight the potential of vdW AHA-RFETs for energy- and area-efficient logic-in-memory computing.

## Results

### Device architecture and functional features

**Fig. 1:**
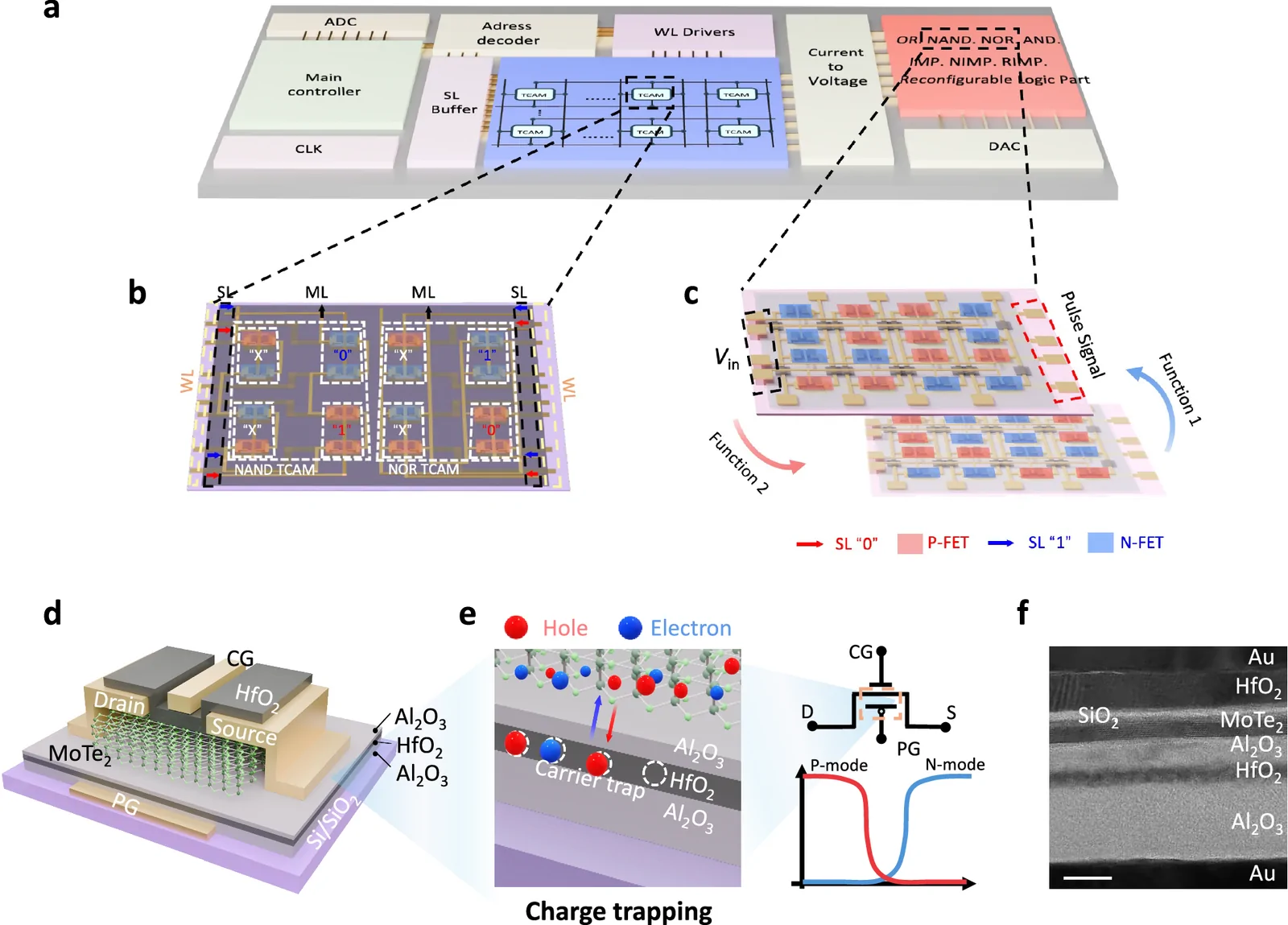
The structure design and functional features of AHA-RFETs. Overall framework of in-memory computing based on AHA-RFET (**a**), including ternary content-addressable memory design (**b**), reconfigurable logic circuit design (**c**). Note that SL, WL, and ML are the abbreviations of search line, word line, and match line. **d** Schematic illustration of the proposed AHA-RFET device, which comprises an ambipolar channel MoTe_2_, an Al_2_O_3_/HfO_2_/Al_2_O_3_ stack charge trapping layer with carrier traps, and a dual-gate structure featuring both a partially covered top gate and a fully covered bottom gate. **e**. Illustrations of the carrier trapping and N/P polarity control that can be realized in the proposed AHA-RFETs. Note that PG and CG are the abbreviations of program gate and control gate. **f**. Cross-sectional scanning transmission electron microscopy image of AHA-RFET. Scale bar, 20 nm.

Emerging in-memory computing paradigms generally demand hardware capable of dynamic logic reconfiguration and high-throughput parallel search, which is enabled by efficient reconfigurable circuits and TCAM (Fig. 1a)^19,34^. RFETs represent a promising path toward such systems by allowing nonvolatile control over channel polarity within a single device. This polarity programmability enables the same physical circuit to implement multiple Boolean logic functions, while also supporting compact TCAM designs through paired RFET configurations. The distinct nonvolatile polarization states inherently generate three distinct matching responses (“1”/“0”/“X”) (Fig. 1b, c).

To realize these functionalities in a scalable manner, we develop a vdW AHA-RFET based on an oxide quasi-floating-gate structure and dual-gate topology. As illustrated in Fig. 1d, the device consists of a multilayer oxide dielectric stack, an ambipolar vdW channel, a localized top control gate (CG), and a global bottom programming gate (PG). Serving as the core polarity-defining element, the AHA quasi-floating gate configuration dynamically modulates the charge transport by regulating electron and hole tunneling and trapping behaviors within the atomically thin Al_2_O_3_ and HfO_2_ layers, respectively^41–43^. The electron affinity mismatch between Al_2_O_3_ and HfO_2_ creates a high tunneling barrier that suppresses interlayer leakage, enabling nonvolatile polarity retention^44^. Furthermore, the scalable top-gate architecture reshapes unipolar conduction behavior by establishing a humped energy band profile that enables precise selection of carrier type (Fig. 1e)^45^.

### Nonvolatile logic reconfigurable modulation

**Fig. 2:**
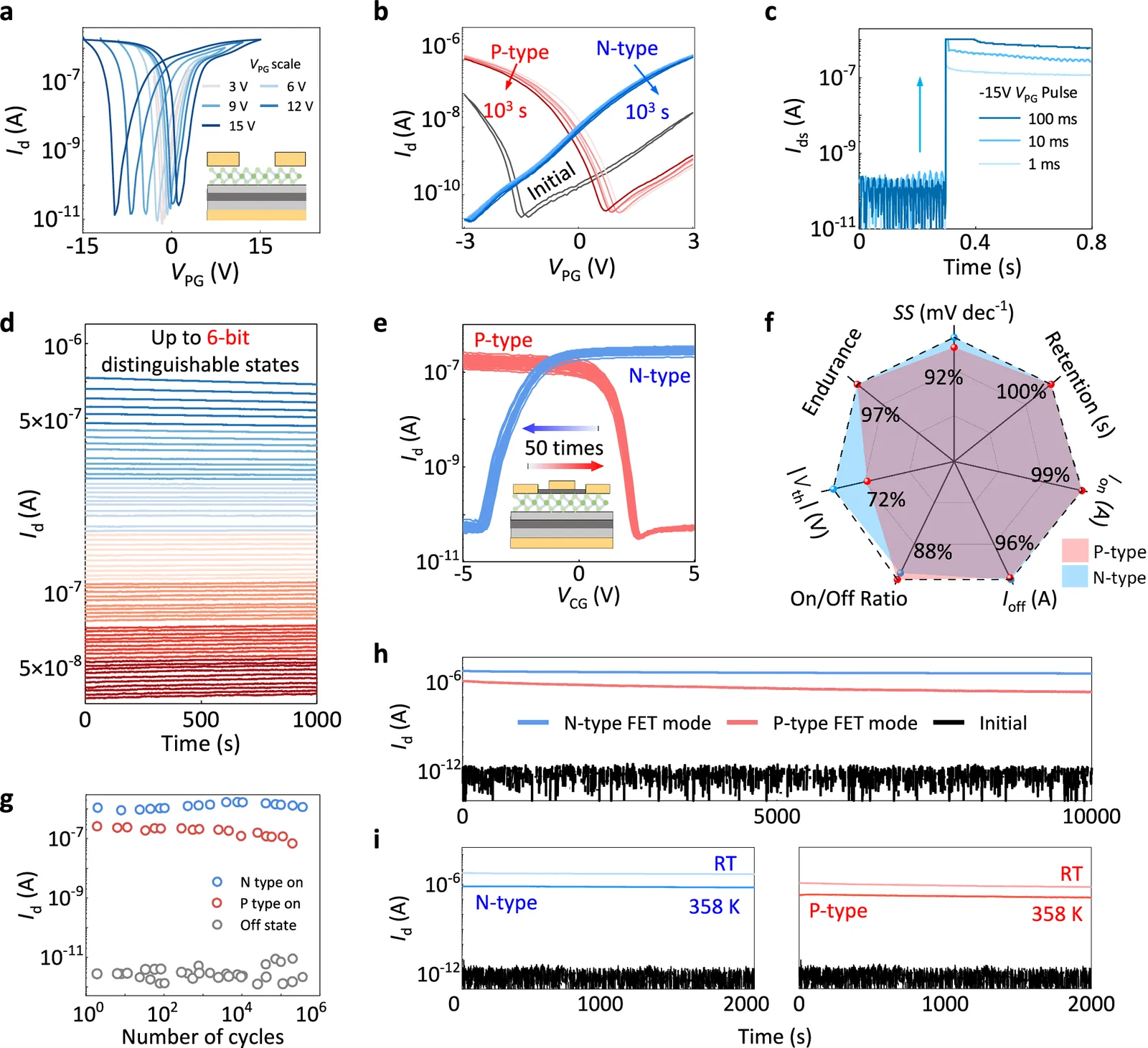
Electrical performance of the AHA-RFET. **a** Transfer characteristics (*I*_d_-*V*_PG_) measured at *V*_ds_ = 0.1 V under different *V*_PG_. A memory window of ∼11 V is defined by the hysteresis in the transfer curves, as observed from the threshold voltage shift during a bidirectional *V*_PG_ sweep from −15 V to +15 V. Inset: Schematic of the single-gate AHA-RFET. **b** Transfer characteristics (*I*_d_-*V*_PG_) of AHA-RFET in initial, P-type, and N-type states, measured at *V*_ds_ = 0.1 V; *V*_PG_ sweep from −15 V to +15 V and recorded at intervals over a period of 10³ s. **c**. Pulse-based programming speed of AHA-RFETs. Transient *I*_ds_ responses under alternating programming (–15 V) pulses with varying widths from 1 ms to 100 ms. **d** Retention performance of multilevel states in AHA-RFET device (measured at *V*_ds_ = 0.1 V and *V*_PG_ = *V*_CG_ = 0 V). **e** The transfer characteristics (*I*_d_-*V*_CG_) under CG control with 50 polarity switching cycles, showing the polarity transition shifts from bipolar to N-type FET mode or P-type FET mode, at *V*_ds_ = 0.1 V and *V*_PG_ = 0 V. Inset: Schematic of the dual-gate AHA-RFET. **f** Radar chart of key device parameters after N/P configuration, highlighting the balanced polarity matching. The on-state current, off-state current, and on/off ratio—parameters exhibiting exponential variation—are evaluated after logarithmic normalization to enable a comparative analysis. Note that SS is the abbreviation of subthreshold swing. **g**. Endurance test of AHA-RFET under hole trapping and electron trapping. The hole trapping/detrapping is repeatedly programmed using cycles with −15 V (100 ms) pulses and 5 V (100 ms) pulses. **h**. Retention characteristics of AHA-RFET in N-type FET mode and P-type FET mode, measured for 10^4^ s at *V*_ds_ = 0.1 V and *V*_PG_ = 0 V. **i** Retention characteristics of AHA-RFET in N-type FET mode and P-type FET mode, measured at temperatures of RT (300 K) and 358 K, *V*_ds_ = 0.1 V and *V*_PG_ = 0 V.

Taking a MoTe_2_-based ambipolar semiconductor as an example, Fig. 1f and Supplementary Figs. 1 and 2 present systematic morphological and compositional characterizations of the fabricated AHA-RFET devices. Cross-sectional transmission electron microscopy (TEM) and elemental mapping analysis reveal a well-defined multilayer device structure, featuring a 6.5 nm MoTe_2_ ambipolar channel and an Al_2_O_3_/HfO_2_/Al_2_O_3_ (8/8/24 nm) dielectric stack atop the PG electrode. This specific 8/8/24 nm AHA configuration is optimized among various thickness designs to precisely balance the trade-off among several critical parameters, such as operating voltage, programming speed, and non-volatile charge retention (Supplementary Fig. 3). Such an optimized dielectric engineering ensures both robust reconfiguration and long-term stability for the designed structure. Atomic force microscopy (AFM) confirms structural integrity (Supplementary Fig. 4), while energy-dispersive X-ray spectroscopy (EDS) verifies the elemental distribution within the oxide-vdW heterostructure (Supplementary Figs. 1 and 2). Figure 2a presents the electrical characteristics of the fabricated AHA-RFET under the PG regime. The device initially exhibits typical ambipolar conduction, with the transfer curve developing a memory window (Δ*V*) of up to 11 V as the PG voltage sweep range increases. This hysteresis is reproducible across the tested devices with top-gate configuration, during which the HfO_2_ layer provides robust encapsulation against moisture and oxygen (Supplementary Fig. 5). This scenario is validated by an 8-month ambient aging test, confirming negligible performance degradation and effective protection of MoTe_2_ (Supplementary Fig. 6). Note that related comparison experiments are conducted within a nitrogen-filled glovebox to exclude the effect of ambient artifacts (Supplementary Figs. 7 and 8).

Reversible polarity switching between N-type (under negative PG pulses) and P-type (under positive PG pulses) behavior is demonstrated in Fig. 2b. Each state retains its characteristics for over 1000 s without distinct degradation, confirming the nonvolatility of the polarity modulation in the proposed AHA-RFET devices. Building on this stable polarity control, we further achieve multilevel conductance manipulation by regulating the trapped charge density via precise *V*_PG_ pulses. As shown in Fig. 2c, pulses as short as 1 ms effectively trigger conductance switching, with current levels scaling correspondingly following the varying pulse duration. Consequently, 64 distinct nonvolatile conductance states (6-bit) are realized (Fig. 2d). Each state remains stable for 1000 s and is reproducibly modulated across a dynamic range of 10^−11^ to 10^−6^ A over multiple cycles, highlighting the AHA stack’s multi-level storage capabilities (Supplementary Fig. 9). The AHA-RFET’s multi-state capability further extends to miniaturized geometries of 460-nm channel length (*L*_ch_), for which the reproducible 6-bit multilevel conductance manipulation demonstrates consistent structural scalability (Supplementary Fig. 10). This performance at reduced dimensions suggests that the AHA stack provides robust electrostatic control, effectively mitigating the stochastic fluctuations typically encountered in short-channel devices. Beyond spatial scalability, the AHA-RFET achieves fully program/erase (P/E) switching under rapid pulses with minimal energy consumption, while maintaining robust reversibility and cyclic endurance without significant fatigue behavior of the atomic tunneling layer (Supplementary Fig. 11). These metrics are highly competitive among AHA-based charge-trapping devices (Supplementary Table 1).

Although bottom-gate operation enables polarity modulation, complete suppression of ambipolar transport remains challenging for most reported RFET devices. To address this, we introduce a top-gate design to precisely shape the energy band landscape and the carrier contributions of the ambipolar semiconductor channel. As expected, the incorporation of a top-gate structure enables unipolar transport for both electron and hole branches, showing enhanced polarity control capabilities. Specifically, based on the initial ambipolar transport, a negative programming pulse triggers hole trapping, favoring N-type dominance, while a positive pulse induces pronounced P-type operation (Supplementary Fig. 12). These reconfigurable characteristics are reproducible over 50 switching cycles, confirming the robustness of device reconfiguration (Fig. 2e). Notably, this synergistic polarity modulation extends beyond MoTe_2_ to WSe_2_ and ReSe_2_, showing that the AHA architecture is applicable to the tested devices (Supplementary Fig. 13). Given the better current matching behavior of P- and N-branches, MoTe_2_ devices are employed for the following logic-circuit demonstrations. Beyond material versatility, the quasi-floating-gate mechanism is independent of the specific AHA combination. By employing an alternative SiO_2_/HfO_2_/SiO_2_ (SHS) stack, we observed similarly consistent non-volatile dynamics and reliable polarity switching (Supplementary Fig. 14). These results indicate that the stack-engineered quasi-floating-gate architecture can support non-volatile polarity modulation in the tested 2D electronic devices.

Engineering the top CG also facilitates a steeper subthreshold swing (SS) and better suppression of ambipolarity compared to PG-only control, due to its segmented band modulation (Supplementary Fig. 15). Multiple analog states spanning four orders of magnitude are attained for both branches (Supplementary Fig. 16). To quantify the balanced electrical characteristics between N- and P-type modes, a radar chart is employed. As shown in Fig. 2f, critical metrics, SS, *V*_th_, *I*_on_, *I*_off_, *I*_on_/*I*_off_, endurance, and retention for both branches reveal nearly overlapping profiles, indicating well-balanced electrical capability enabled by dual-gate control (with details in Supplementary Fig. 17). For example, the on-state current mismatch between the two modes is as low as 1%, with a similarly small off-state current mismatch (4%), meeting the performance requirements for high-quality reconfigurable logic circuits (Supplementary Note 1).

Device reliability of the proposed AHA-RFETs, a critical metric for practical implementation, is further assessed through endurance and retention tests. As illustrated in Fig. 2g, the device can reproducibly achieve two complementary current levels for P- and N-type modes over 3 × 10^5^ switching cycles with negligible degradation. Programmed states also exhibit stable retention, maintaining on/off ratios exceeding 10^6^ for over 10^4^ s (Fig. 2h). More importantly, this AHA-enabled reconfigurable design demonstrates strong thermal stability, with consistent behavior from room temperature (RT, 300 K) to 358 K for 2000 s (Fig. 2i), which is a standard temperature for practical working environments of memory devices. This thermal resilience suggests stable charge trapping dynamics below the critical temperature threshold for practical working environments of memory devices.

### Physical mechanisms based on device simulation

Understanding the underlying physical mechanisms of RFETs is essential for their precise control and reliable application in in-memory computing. To this end, we perform a systematic investigation into the reconfigurable dynamics of the AHA-RFET using Technology Computer-Aided Design (TCAD) simulations. A device model incorporating the AHA stack is established to elucidate the origin of its nonvolatile polarity reconfigurability (Supplementary Fig. 18 for simulation details). The simulated transfer characteristics under PG sweeping initially exhibit ambipolar behavior with a clear memory window. By introducing a charge-trapping model in the AHA region, the curve can be reproducibly shifted between N-type and P-type states, showing good agreement with experimental measurements.

**Fig. 3:**
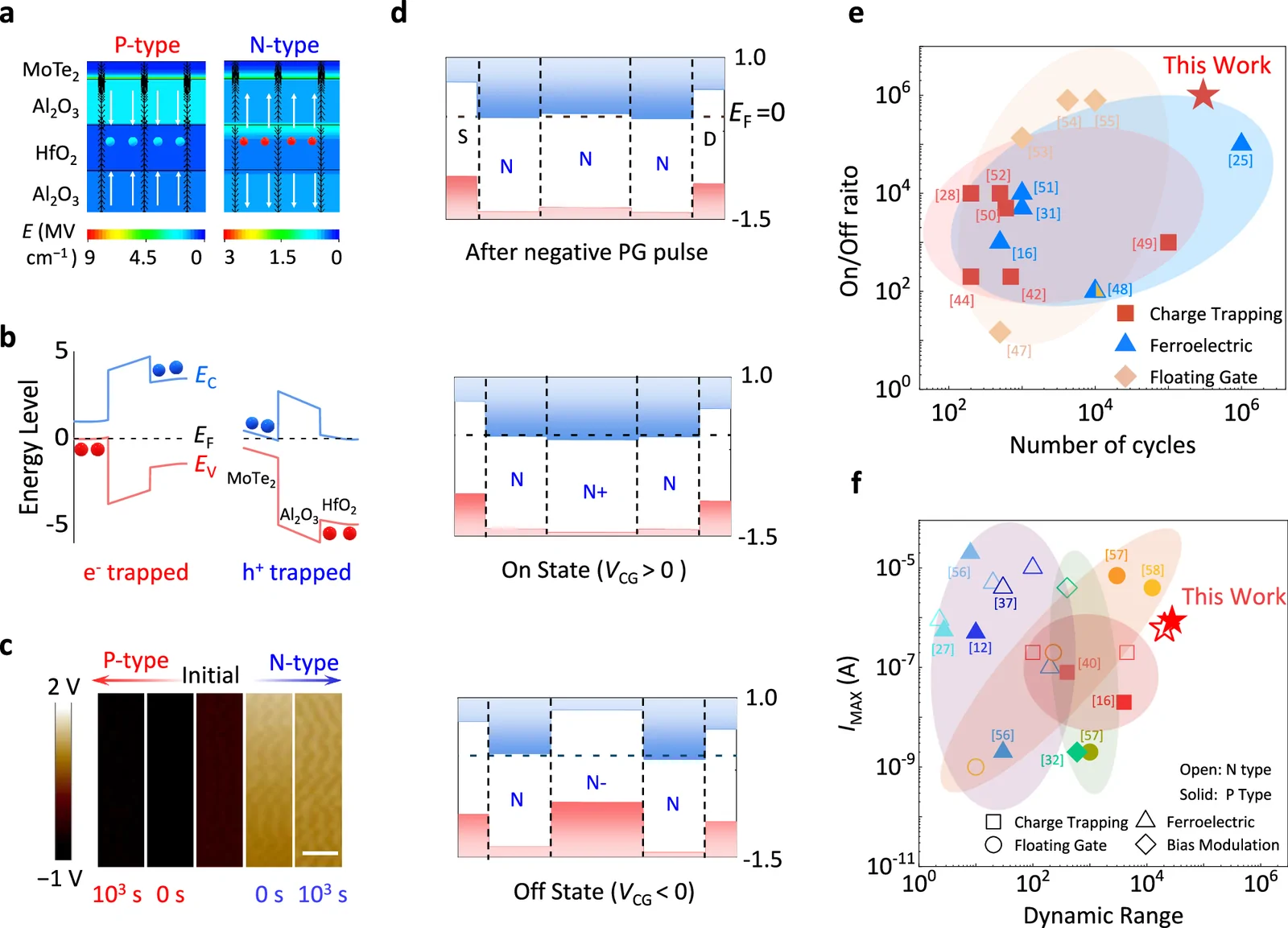
Mechanism Analysis of the AHA-RFET. Simulated electric field (*E*) contour plots (**a**) and energy band diagrams (**b**) of the MoTe_2_/Al_2_O_3_/HfO_2_/Al_2_O_3_ stack under electron-trapped (left panel) and hole-trapped (right panel), respectively. Note that *E*_F_, *E*_C_, and *E*_V_ are the abbreviations of Fermi level, conduction-band edge, and valence-band edge. **c** Time-resolved KPFM characterization of surface potential dynamics. Mapping of surface potential evolution over 10^3 ^s for the device in its initial, P-type (+10 V pulse), and N-type (−10 V pulse) states. Scale bar, 100 nm. **d** Simulated energy band diagrams at the drain, MoTe_2_, and source region under CG control. AHA-RFET is configured in N-type FET mode. **e** Performance benchmarking of the proposed AHA-RFET against previously reported reconfigurable and nonvolatile devices, comparing the endurance cycle number and the current on/off ratio after cycling. **f** Comparison of the single-polarity dynamic range and maximum on-current in the corresponding dynamic range (*I*_max_) with literature values. The single-polarity dynamic range is defined as the (*I*_max_/*I*_min_) ratio after unipolar reconfiguration and represents the effective polarity switching window.

Figure 3a displays the corresponding electric field contour, revealing two distinct built-in electric fields within the AHA region after opposite gate pulses. Considering that the existence of intrinsic oxygen defects in the HfO_2_ layer can disturb the trapping states and the transport carriers, this scenario can be understood as follows^46^. Electrons or holes driven by a positive or negative voltage pulse would tunnel through the Al_2_O_3_ layer and be trapped in the HfO_2_ layer. A positive PG pulse traps electrons, while a negative PG pulse traps holes, during which the generated electric field nonvolatility manipulates energy band bending behaviors, hence determining the channel carrier type and conductivity. As visualized in Fig. 3b, simulated band diagrams show that hole trapping lowers the conduction band minimum below the Fermi level (*E*_F_), enhancing electron conduction, while electron trapping raises the valence band maximum toward *E*_F_, favoring hole transport. Besides the energy band analysis, time-resolved KPFM further captures the non-volatile charge storage kinetics within the AHA stack. As shown in Fig. 3c, upon applying ±10 V pulses, the surface potential shifts to −950 mV/+1.8 V and remains stable at −700 mV/+1.2 V after 10^3 ^s, confirming robust retention and operational stability under ambient conditions. To probe the microscopic origin of nonvolatile reconfigurability and interface quality, low-frequency noise (LFN) characterizations are further performed (Supplementary Fig. 19). The measured noise power spectral density exhibits a distinct 1/*ƒ* dependence, and the normalized noise (*S*_I_/*I*_ds_^2^) remains nearly independent of the drain bias, confirming that the dynamic charge disturbances in the devices are governed by trapping/detrapping at the AHA stack rather than the source-drain contact or carrier mobility variations. This validates the high-quality, low-defect nature of the heterointerface and the precise electrostatic control afforded by the AHA stack.

Beyond programming gate configuration, the critical role of the top gate design in tailoring energy band profiles and unipolar transport is also investigated. As shown in Fig. 3d and Supplementary Fig. 20, the incorporation of the top-gate control enables local band reshaping under different operating states. Taking N-type FET mode as an example, initial programming with a negative PG pulse converts the channel from ambipolar to electron-dominated transport, accompanied by a 0.5 eV downward shift of the MoTe_2_ conduction band relative to *E*_F_, establishing a stable N-type configuration. Subsequent CG pulses demonstrate two distinct operational regimes: a negative CG pulse elevates the band by 0.7 eV, forming an N-N^−^-N potential barrier that pinches off the channel; a positive CG pulse further lowers the band by 0.1 eV, creating an N-N^+^-N structure that facilitates electron conduction. A similar evolution for the P-type mode is provided in Supplementary Fig. 20. As expected, the simulated fundamental transfer characteristics of the dual-gate RFET exhibit well-defined complementary conduction, aligning closely with experimental results (Supplementary Fig. 18).

Based on the achieved device characterization and clear physical modeling, we benchmark the proposed AHA-RFET against previously reported reconfigurable and memory devices across several key metrics. As summarized in Fig. 3e, our AHA-RFET shows higher cycling endurance than the compared reconfigurable strategies, such as those based on ferroelectric or floating-gate structures—a benefit attributed to the robust charge tunneling and trapping characteristics enabled by the AHA dielectric stack^16,25,28,31,42,44,47–55^. In terms of multi-state programmability, our device achieves 64 distinct conductance levels with stable analog retention (Supplementary Fig. 21). Moreover, by enabling flexible modulation of the energy band landscapes of narrow-bandgap MoTe_2_, the AHA-RFET device exhibits an enlarged dynamic range and well-balanced P/N reconfigurability, outperforming most reconfigurable devices (Fig. 3f)^12,16,27,32,37,40,56–58^. These combined merits underscore the potential of AHA-RFETs in enabling reliable and versatile in-memory computing systems.

### Reconfigurable logic circuits with scalable devices

Leveraging the nonvolatile polarity reconfiguration and multi-state modulation capabilities of the proposed AHA-RFET, we demonstrate its practical application in logic-in-memory computing. Logic-in-memory computing and TCAM capability represent two critical tasks for developing efficient computing technologies to overcome the bottlenecks of the von Neumann architecture^21,40^. While nonvolatile reconfigurable devices enhance circuit adaptability, their development has been hampered by current mismatch during asymmetric polarity switching—a particular concern for parallel search operations in TCAM. Furthermore, achieving uniform device characteristics in vdW-based RFETs, especially with top-gate integration for high-density circuits, remains challenging^38^.

**Fig. 4:**
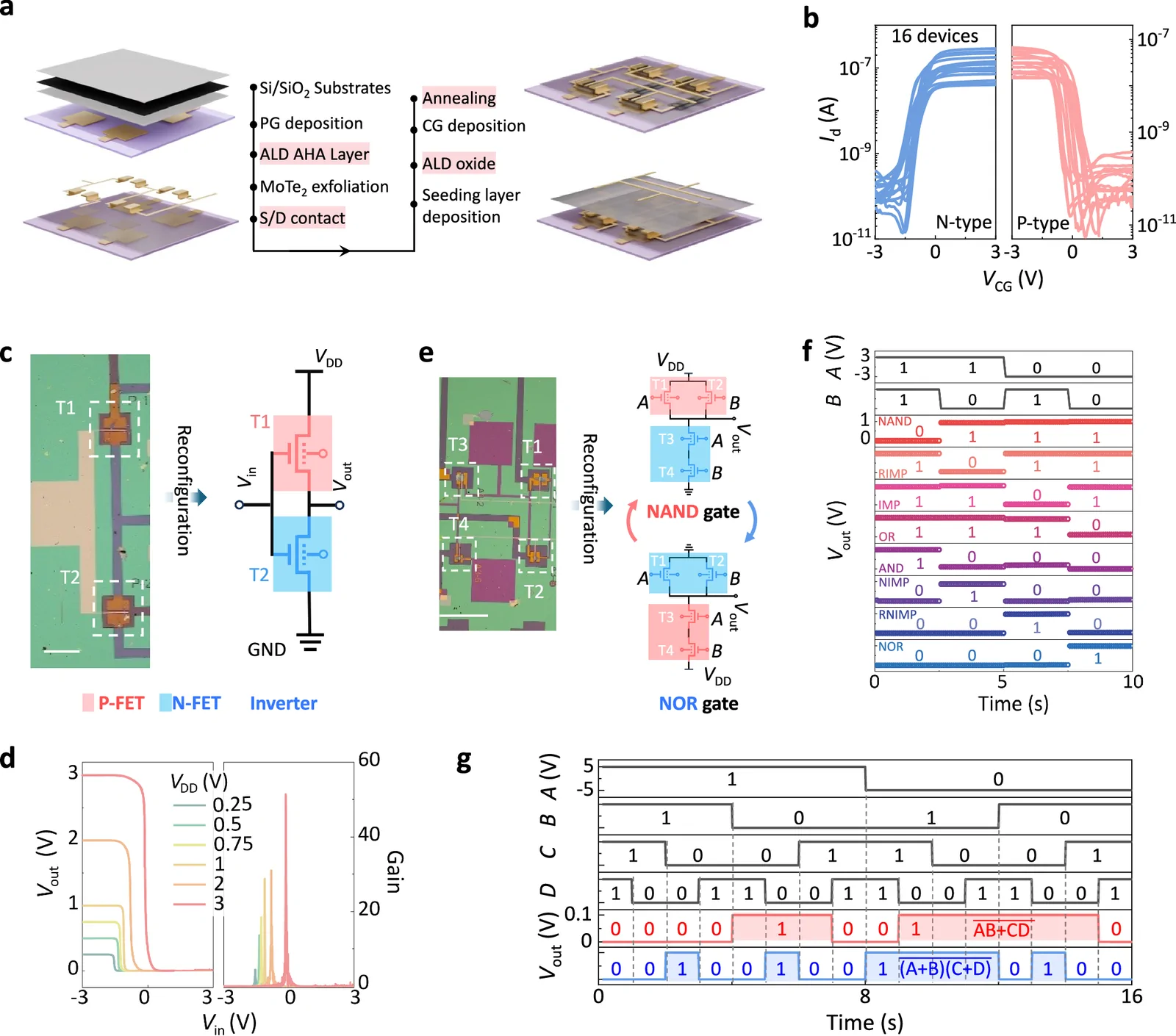
Reconfigurable logic-gate circuits implemented on the AHA-RFETs. **a** Fabrication process flow of AHA-RFET-based reconfigurable circuits. Note that ALD and S/D are the abbreviations of atomic layer deposition and source/drain electrodes. **b** Statistical results of reconfigurable transfer characteristics (*I*_d_-*V*_CG_) of 16 AHA-RFET devices. All devices are successfully programmed into N-type mode and P-type FET mode by applying a −15 V (100 ms) and +15 V (1 s) pulse on PG, respectively. All measurements are performed at *V*_ds_ = 0.1 V and *V*_PG_ = 0 V. **c** Schematic and optical image of a reconfigurable circuit containing two AHA-RFETs. Scale bar: 100 μm. Inverter mode is configured by programming the pull-down and pull-up transistors as N-type FET mode and P-type FET mode with PG voltage pulses of −15 V (100 ms) and +15 V (1 s), respectively. **d** Voltage transfer characteristics (left panel) and corresponding voltage gains (right panel) of inverter mode under *V*_DD_ varying from 0.25 to 3.0 V. **e** Schematic of the circuit and optical image of a 4T reconfigurable circuit. Scale bar: 200 μm. Programming with PG voltage pulse of −15 V, 100 ms for N-type FET mode and 15 V, 1 s for P-type FET mode. **f** Experimental output waveforms of the reconfigurable logic circuit demonstrating eight Boolean logic functions: NOR, RNIMP, NIMP, AND, NAND, IMP, RIMP, and OR. **g** Simulated waveforms of the 8T reconfigurable gate, verifying its function as both an AOI logic ($\overline{{\mbox{AB}}+{\mbox{CD}}}$) and an OAI (OR-AND-INVERT) logic ($\overline{({\mbox{A}}+{\mbox{B}})({\mbox{C}}+{\mbox{D}})}$), based on experimental device data.

To address these issues, we employ a low-temperature ALD process to fabricate top-gated AHA-RFETs, providing a reliable pathway for integrating reconfigurable functionality into logic-in-memory and TCAM circuits. The fabrication process for homogeneous MoTe₂ AHA-RFET logic circuits is illustrated in Fig. 4a. It begins with programming-gate patterning for independent polarity control, followed by deposition of the high-quality AHA dielectric stack. The use of low-temperature ALD for top-gate dielectric and interconnects preserves the reconfigurable properties of the vdW channel (Methods; Supplementary Fig. 22). Due to the surface dangling bondless feature, a critical component of this fabrication process is the deposition of a 3-nm SiO_2_ seeding layer (SL) prior to HfO_2_ growth to ensure a dense, continuous dielectric film (Supplementary Fig. 23). It is worth mentioning that, although the precise carrier modulation and synthetic approaches of ambipolar materials currently require further optimization, the AHA-stack structure establishes a robust and scalable manufacturing route for reconfigurable electronic devices (Supplementary Fig. 24). Thus, high-quality exfoliated MoTe_2_ was utilized to achieve optimal current matching and rigorously evaluate the intrinsic physics of the AHA-RFET device. As expected, the resulting RFETs exhibit polarity tunability and symmetric N-type/P-type behavior, essential for high-performance logic circuits (Fig. 4b). Using these devices, various reconfigurable logic functions are implemented through flexible deployment of RFET units. Figure 4c displays the built complementary inverter consisting of a couple of MoTe_2_ RFETs. Under opposite programming operations, where P-type FET mode unit serves as the pull-up transistor and the N-type FET mode unit serves as the pull-down transistor, the inverter cell exhibits a full-swing output from 0.25 to 3 V. Thanks to the matched electrical properties of two branches, a high voltage gain of 51 is obtained at a supply voltage (*V*_DD_) of 3 V (Fig. 4d), with power consumption at the nanowatt level enabled at *V*_DD_ of 1 V (Supplementary Fig. 25).

Beyond basic inverter functionality, the architectural versatility of AHA-RFETs enables more complex logic-in-memory implementations. As shown in Fig. 4e, a 4T logic-gate circuit comprising two parallel-connected and two series-connected AHA-RFETs is designed. This homogeneous circuit can be programmed into NAND or NOR gates by configuring ambipolar channels into unipolar transistors. Using 3 V and −3 V as logic “1” and “0”, the output is determined by complementary conduction paths. This functionality relies on precisely engineered asymmetric charge trapping to establish opposite polarities in paired RFETs. The same hardware can thus implement eight distinct Boolean logic operations (NAND, NOR, AND, OR, IMP, RIMP, NIMP, RNIMP) using identical input pairs (Fig. 4f and Supplementary Fig. 26). This all-active architecture, which uses only AHA-RFETs and eliminates passive components, reduces fabrication complexity and enhances integration density compared to hybrid designs such as the 2T1R (two-transistor-one-resistor) configurable logic gate.

While the ALD-based top-gate engineering provides a promising route for scalable RFET device fabrication, the current reliance on mechanically exfoliated ambipolar MoTe_2_ flakes limits circuit demonstrations. To explore more complex logic-in-memory circuits, we perform TCAD simulations of an 8T reconfigurable gate (8 transistors, Supplementary Fig. 27). This circuit integrates series/parallel subcircuits to process all 16 possible input combinations (0000-1111) and can be dynamically programmed to implement either AND-OR-Invert (AOI, ($\overline{{\mbox{AB}}+{\mbox{CD}}}$)) or OR-AND-Invert (OAI, ($\overline{({\mbox{A}}+{\mbox{B}})({\mbox{C}}+{\mbox{D}})}$)) logic via bias control (Fig. 4g). Extended functionalities, such as (A + B) ($\overline{{\mbox{CD}}}$) and ($\overline{{\mbox{A}}}\overline{{\mbox{B}}}$ + CD), are also verified through partial reconfiguration schemes (Supplementary Fig. 28). Unlike silicon circuits with fixed functionality dictated by physical layout, this reconfigurable architecture can adapt to diverse circuit requirements, enabling hardware resource allocation towards efficient in-memory computing architectures.

### Compact ternary content-addressable memory

**Fig. 5:**
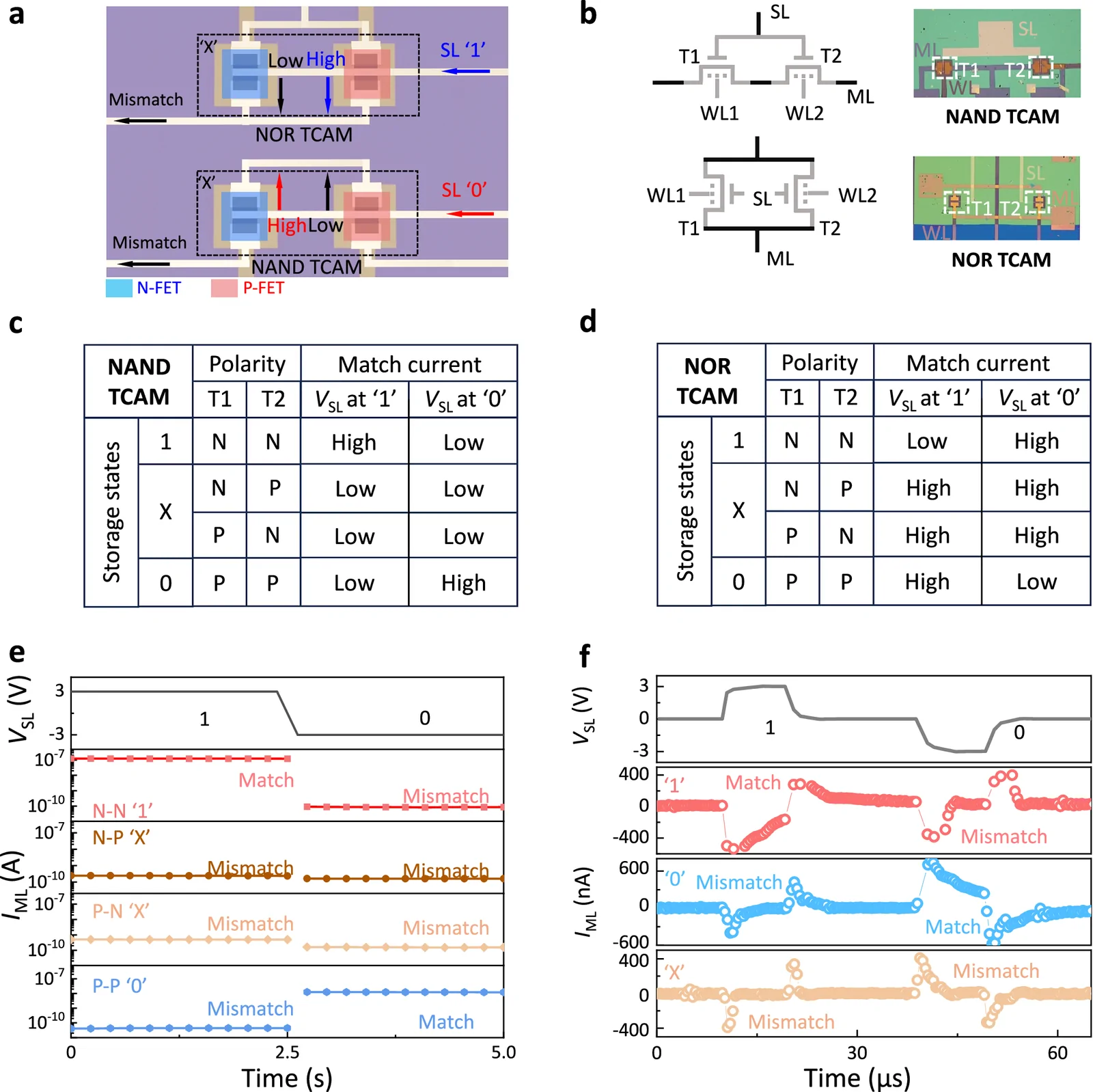
Implementations of ternary content-addressable memory based on AHA-RFET circuits. **a** Schematic illustration of the match line (ML) current under normal operating conditions, demonstrating how *V*_SL_ and the polarity state in the AHA-RFET affect conduction. High: high current; Low: low current; X: don’t care. **b** Schematic and optical images of NAND TCAM and NOR TCAM. State tables of the NAND TCAM (**c**) and NOR TCAM (**d**) for ternary storage and search operation in the TCAM cell. Transistor polarity serves as stored information for search and match operations. The output is always mismatched when the state is bit “X” (don’t care). **e** Experimental results of NAND TCAM cell with two AHA-RFETs, measured at *V*_ML_ = 0.1 V. Two AHA-RFETs are programmed in P-P, P-N, N-P, and N-N states, respectively. **f** Experimental demonstration of non-volatile TCAM searching operations. The timing diagrams illustrate the relationship between the *V*_SL_ and the resulting *I*_ML_ for various logic configurations, including N-N “1”, P-P “0”, and “X”.

TCAM as a hardware accelerator for parallel data search benefits the in-memory computing realization, especially for data-intensive applications. A conventional TCAM cell typically requires at least 16 transistor units, which severely compromises the area and energy efficiency benefits of in-memory computing^59^. Distinctly, the incorporation of reconfigurable devices simplifies TCAM design by leveraging programmable polarization states to generate the required matching response. Specifically, the polarity-reconfigurable nature of AHA-RFET directly governs the match line (ML) current response to search line (SL) voltage inputs. For example, a device in the N-type FET mode produces a high ML current for SL = 3 V (“1”). Conversely, a device in the P-type mode yields a high ML current for SL = −3 V (“0”). This intrinsic behavior allows a pair of AHA-RFETs to encode the don’t care (“X”) state through complementary configurations (Fig. 5a), thereby drastically reducing the transistor count compared to the conventional 16T TCAM cell.

Following this operating principle, we implement both NAND-type and NOR-type TCAM cells using different matching schemes (Fig. 5b). For example, a NAND-type TCAM cell can be constructed by two series-connected AHA-RFETs, where the corresponding CG and individual PGs serve as the SL and word lines (WL1 and WL2), respectively, with the series connection forming the ML. Both NAND and NOR cells can achieve ternary storage (“1”/“0”/“X”) and perform precise search/matching operations through dynamical polarity programming. Specifically, the search/matching operations of the NAND-type TCAM require SL input polarity to match stored data (“1” = both reconfigured as N-type FET mode: SL = “1” turn on FETs for high ML current (Match); SL = “0” cuts them off; “0” = both reconfigured as P-type FET mode: SL = “0” conducts (Match), SL = “1” cuts off), while “X” (mixed polarities) always cuts off. Conversely, the NOR-type inverts this logic: stored “1” (both P-type FET mode)/“0” (both N-type FET mode) induce FET cutoff with matched SL (low ML current (Match)), whereas mismatched SL or “X” maintains conduction (high ML current (Mismatch)) (Fig. 5c, d). As a result, through this operation, both NAND and NOR TCAM designs, built with two AHA-RFETs each, can independently perform the full set of functions required for search and matching operations. The measured results for two kinds of RFET-based TCAM cells are shown in Fig. 5e and Supplementary Fig. 29. Both designs exhibit reliable search-matching characteristics with a clear distinction between match and mismatch currents (∣*I*_match_/*I*_mismatch_∣ > 10^2^), ensuring operational reliability for in-memory computing acceleration. The searching speed of the AHA-RFET NAND TCAM is experimentally characterized (Fig. 5f). Although its microsecond-range search speed is currently limited by testing environment RC delays, as summarized in Supplementary Table 2, our device achieves a stable searching operation of 10 μs, which is highly competitive and comparable to recent state-of-the-art reports for TCAMs. Furthermore, the AHA-RFET TCAM provides a highly area-efficient solution, reducing the cell footprint to 2T from the conventional 16T requirement.

By achieving diversified logic reconfiguration and full TCAM functionality using a single type of transistor, our AHA-RFETs break the fixed-functionality constraint of traditional MOSFET, highlighting its immense potential in streamlining compact circuit design and enhancing architectural adaptability. Such comprehensive logic reconfigurability not only provides post-fabrication flexibility but also positions the device as a highly attractive candidate for next-generation adaptive logic-in-memory computing systems.

## Discussion

In summary, we demonstrate a scalable reconfigurable transistor based on an oxide–van der Waals quasi-floating-gate architecture, which enables nonvolatile multi-state programmability and polarity control through a silicon-compatible top-gate dielectric engineering approach. By leveraging engineered charge trapping in the Al₂O₃/HfO₂/Al₂O₃ stack, the device achieves high-performance logic-computing characteristics, including nonvolatile analog conductance updates (up to 6 bits for 1000 s), robust endurance (>3 × 10^5^ cycles), and well-balanced electron and hole transport (current mismatch ratio of 1%). TCAD simulations combined with electric field and energy band analysis reveal the underlying mechanism of multi-state storage and polarity reconfiguration. Exploiting the scalable ALD-based top-gate process and symmetric complementary behavior, we further realize a variety of reconfigurable logic circuits, such as inverters with a voltage gain of 51, multifunctional gates supporting eight Boolean operations, and complex AOI/OAI logic blocks. Moreover, the AHA-RFET enables a compact TCAM design, enabling efficient parallel search in memory-intensive applications. These results underscore the potential of our reconfigurable transistor technology to enhance computational flexibility and energy efficiency, providing a viable pathway toward future adaptive computing and multifunctional integrated systems.

## Methods

### Device fabrication

The AHA-RFET device is fabricated on a pre-cleaned Si/SiO_2_ substrate. First, the bottom gate region is patterned by electron beam lithography (EBL), followed by the deposition of 5/30 nm Ti/Au metal electrodes via electron beam evaporation. After lift-off to remove the photoresist and excess metal, the sample is annealed at 200 °C for 20 min in N_2_ ambient to eliminate residual photoresist. Next, a high-κ dielectric stack consisting of Al_2_O_3_/HfO_2_/Al_2_O_3_ (8/8/24 nm, from top to bottom) is deposited via ALD at temperatures below 200 °C. The MoTe_2_ active layer is prepared by mechanical exfoliation and transferred onto the substrate using a polydimethylsiloxane (PDMS)-assisted dry transfer method. Subsequently, source/drain (S/D) electrodes are defined by EBL, and 30 nm Au is deposited via electron beam evaporation. After lift-off, the device undergoes another 200 °C, 20 min N_2_ annealing to improve metal-semiconductor contact. To enhance gate insulation, a 3 nm seeding layer is deposited before ALD growth of 10 nm HfO_2_. Finally, interconnect gate electrodes are patterned by EBL, and 30 nm Au is deposited.

### Structural analysis and electrical characterization

The cross-section morphology of the fabricated device is prepared by a focused ion beam-scanning electron microscope (FIB–FESEM, Helios G4 UX) and characterized by a transmission electron microscope (TEM; JEOL–JRM300 operated at 300 kV), equipped with an Energy-dispersive X-ray spectroscopy (EDS) system. The AFM and KPFM images of the devices are measured by an AFM system (Bruker Icon). Electrical characterization of the AHA-RFET is performed in a probe station (TTPX, Lake Shore Cryotronics Inc.) equipped with a semiconductor parameter analyzer (Keysight B1500A). Source Measurement Units (SMUs) and Waveform Generator/Fast Measurement Units (Keysight B1530A) are built into the analyzer to meet experimental testing needs. To prevent unnecessary fluctuations caused by the measurement environment, all the electrical tests are performed in a vacuum (<10^−5^ Torr) in the dark.

### Device simulation and reconfigurable gate design

The simulations are performed using the TCAD platform of Sentaurus. MoTe_2_-based AHA-RFETs are constructed according to the structural dimensions illustrated in Fig. 1d–f, with key material parameters provided in Supplementary Fig. 18a. An 8T reconfigurable gate circuit is subsequently built based on the schematic in Supplementary Fig. 27. The intrinsic transfer characteristics of the MoTe_2_ RFETs (*V*_PG_ = 0 V) are calibrated using the thermionic-field emission model at the source and drain electrodes. Parameters, including carrier mobility, interface trap density, and Schottky barrier height, are adjusted to align the simulated behavior with experimental data. To model the bottom-gate modulation of the RFETs, the Poole–Frenkel tunneling mechanism is incorporated into the AHA stack, enabling the simulation of electron and hole trapping and release dynamics in the HfO_2_ layer under *V*_PG_ bias. Transient simulations are conducted with a drain-source voltage *V*_ds_ = 0.1 V, while the programming gate voltage *V*_PG_ is swept from −15 to +15 V to capture the bottom-gate transfer characteristics. The *I*_d_–*V*_PG_ curves are extracted by simulating program/read sequences followed by transient analysis. Finally, the 8T reconfigurable gate circuit is evaluated under different RFET configuration states. Input signals are applied to terminals A, B, C, and D, and the output voltage *V*_out_ is monitored to assess the circuit-level reconfigurable logic performance, as summarized in Supplementary Fig. 28.

## Supplementary information

Supplementary Information

Transparent Peer Review file

## Data Availability

The Source Data underlying the figures of this study are available at 10.6084/m9.figshare.32768607.

## Code availability

The simulation code used in this study is available at 10.6084/m9.figshare.32768607.
