# Supplementary material for: Dielectric stacking-engineered scalable reconfigurable transistor platform for adaptive logic circuits
Source: Nat Commun. 2026 Jul 24;17:9028. doi: 10.1038/s41467-026-75864-2 (PMC13507052; doi:10.1038/s41467-026-75864-2)
Supplement: Supplementary file 1 [file 41467_2026_75864_MOESM1_ESM.pdf]

Supplementary Information

Dielectric stacking-engineered scalable reconfigurable transistor platform for adaptive logic circuits

Pengfei Zhu[1#], Chi Zhang[1#], Jingbo Yang[1#], You-Wei Guo[2,3], Shida Huo[4], Enxiu Wu[4*], Fei Wang[1], Che-Yi Lin[5], Jyun-Hong Chen[6], Hongling Chu[1], Zhaorui Liu[1], Song Zhao[1], Jun Li[1], Mengjiao Li[1,7*], Yen-Fu Lin[2,3*], Jianhua Zhang[1]

[1]School of Microelectronics, Shanghai University, Jiading, Shanghai 201800, China;

[2]Department of Physics, National Chung Hsing University, Taichung, 40227, Taiwan;

[3]Graduate Program in Semiconductor and Green Technology, Academy of Circular Economy, National Chung Hsing University, Nantou City, Nantou County 540216, Taiwan;

[4]State Key Laboratory of Precision Measurement Technology and Instruments, School of Precision Instruments and Opto-electronics Engineering, Tianjin University, Tianjin 300072, China;

[5]Department of Applied Physics, Tunghai University, Taichung 407224, Taiwan;

[6]National Applied Research Laboratories, Taiwan Semiconductor Research Institute, Hsinchu, Taiwan;

[7]Shanghai Key Laboratory of Automotive Intelligent Connected Interaction Chips and Systems, Shanghai 201800, China

[#]These authors contributed equally: Pengfei Zhu, Chi Zhang, Jingbo Yang

[*] Corresponding Authors

E-mail: enxiuwu@tju.edu.cn (E.W.); mjli@shu.edu.cn (M.L.); yenfulin@nchu.edu.tw (Y.-F.L.)

This Supplementary Information file includes:

Supplementary Figures 1 to 29

Supplementary Tables 1, 2

Supplementary Note 1

Supplementary References 1 to 33

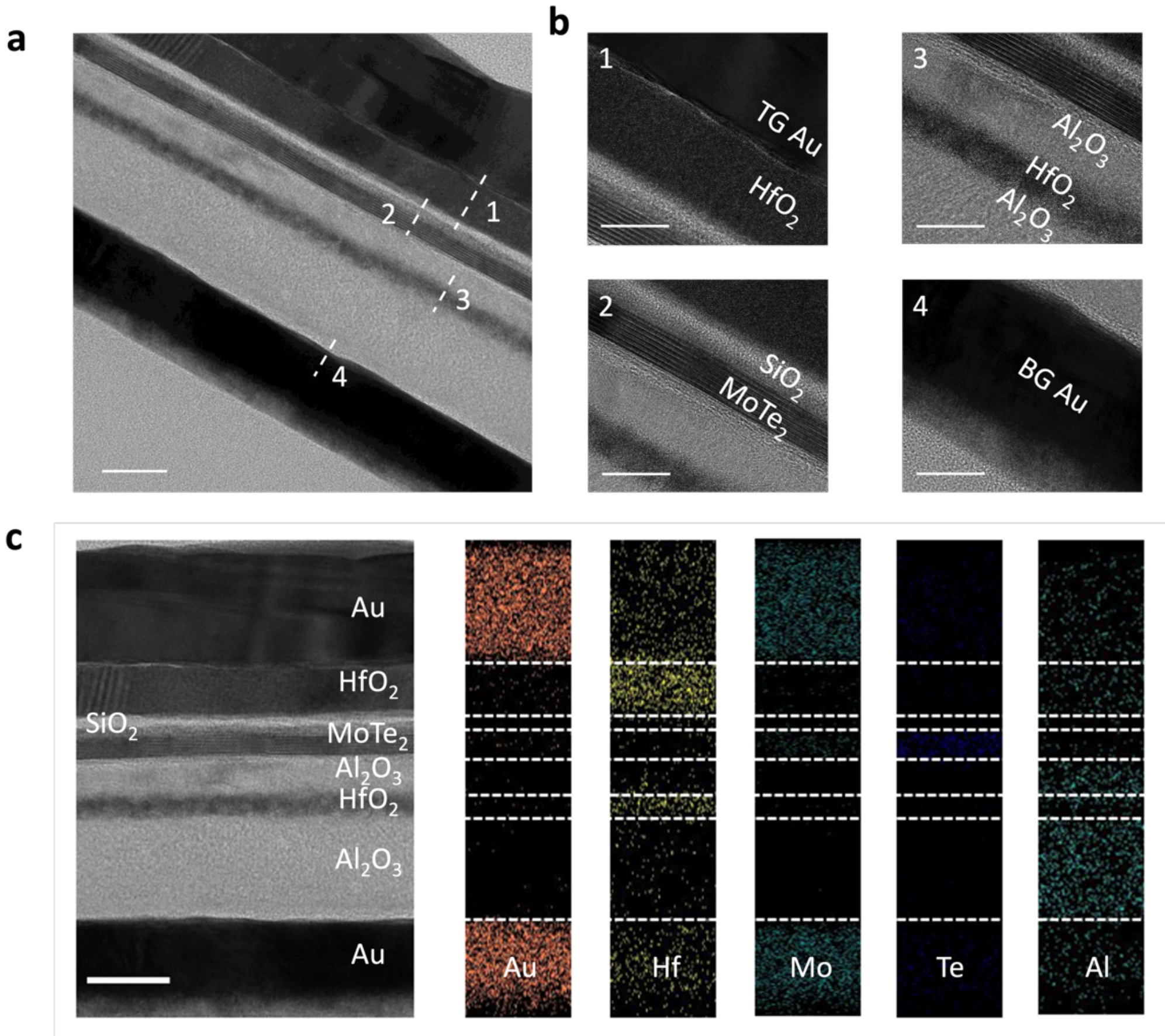

Supplementary Figure 1 Multilayer heterostructure of AHA-RFET between the top and bottom gates. a, b. Cross-sectional scanning transmission electron microscopy image **(a)** between the program and control gates, and corresponding energy-dispersive spectrometry mapping **(b)** considers the existing elements of Au, Hf, Mo, Te, and Al. Scale bars, 20 nm in a, 10 nm in b, and 20 nm in c.

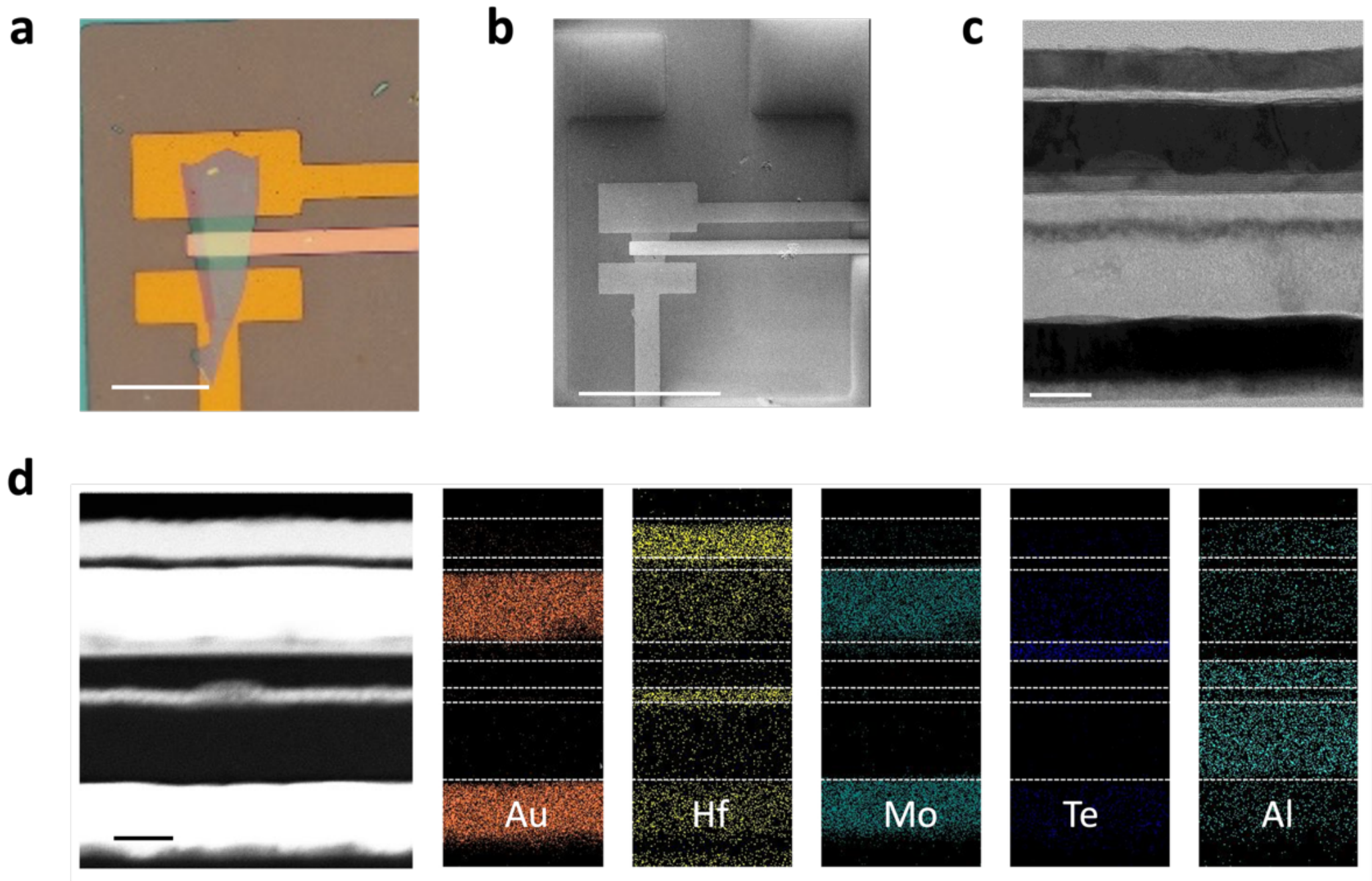

Supplementary Figure 2 Structural and material characterization of AHA-RFET. a, b. The optical microscopy (**a**) and scanning electron microscopy (**b**) images of an AHA-RFET device. **c, d.** Cross-sectional scanning transmission electron microscopy image **(c)** of source/drain electrode region, and corresponding energy-dispersive spectrometry mapping **(d)** considers the existing elements of Au, Hf, Mo, Te, and Al. Scale bars, 20 µm in a, 50 µm in b, 20 nm in c and d.

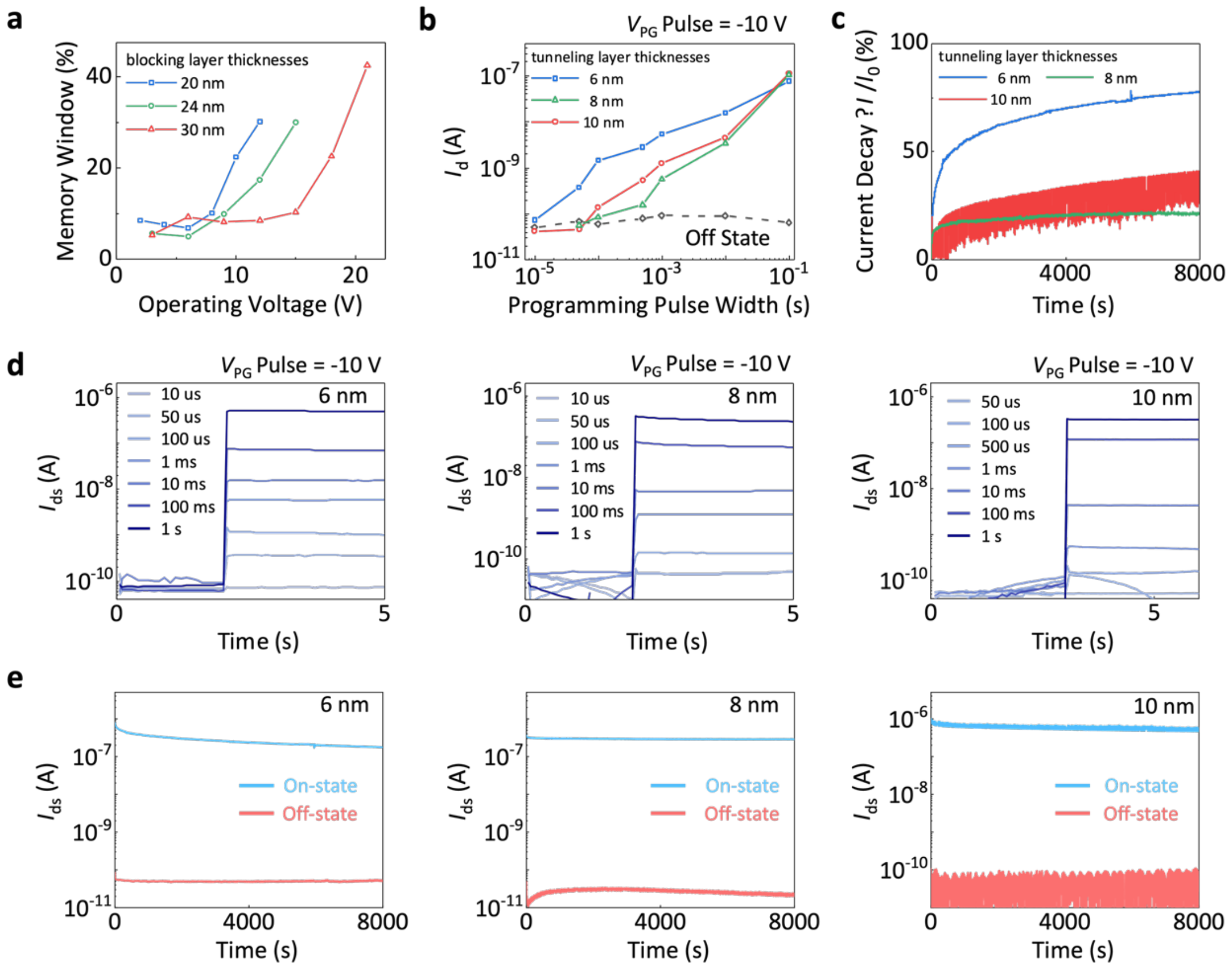

Supplementary Figure 3 Systematic optimization and justification of the AHA dielectric stack thicknesses. a. The variation of the memory window with operating voltage under different blocking layer thicknesses, showed that a thinner blocking layer requires a lower voltage to achieve a same proportional memory window. **b.** The variation of drain current response with programming pulse width under different tunneling layer thicknesses, demonstrated that a thinner tunneling layer significantly accelerates the programming response speed. **c.** The variation of current decay over time under different tunneling layer thicknesses, indicated that optimizing the tunneling layer thickness effectively suppresses current loss to enhance data retention. **d.** Transient I_d response to pulses of varying widths, reveals that the 6 nm tunneling layer enables fast switching within 50 µs, whereas the 10 nm version requires significantly longer pulses (up to 500 µs) to achieve a similar current response. **e.** Retention tests of the on-state and off-state over 8,000 s, demonstrate the 6 nm tunneling layer leads to noticeable current decay.

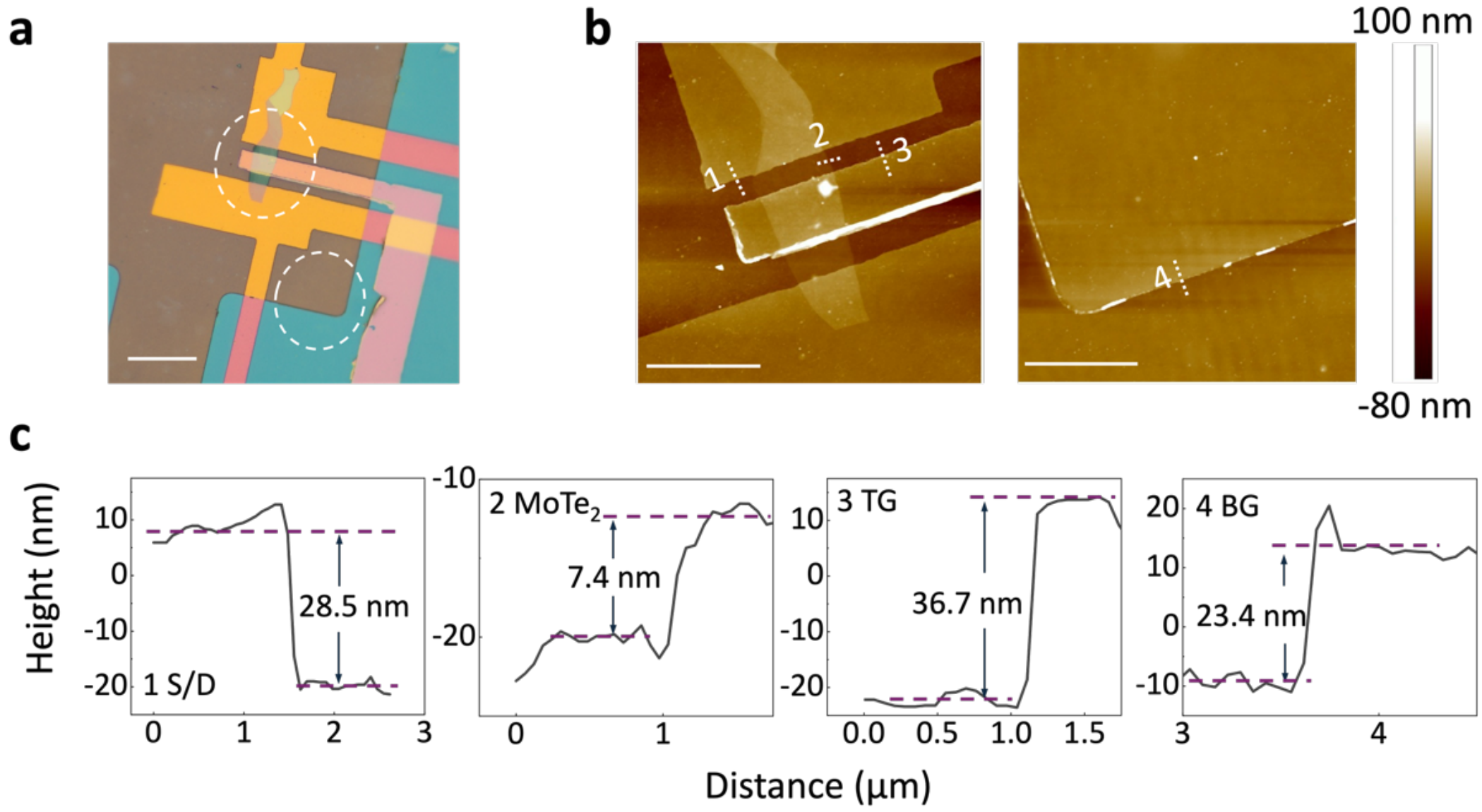

Supplementary Figure 4 AFM images of AHA-RFET. a, b. Optical microscope (**a**) and AFM (**b**) image of an AHA-RFET **c.** Height profiles of Source/Drain, $MoTe_2$, TG and BG. The thicknesses of the components are 28.5, 7.4, 36.7 and 23.4 nm, respectively. Scale bars, 20 µm in a, 10 µm in b.

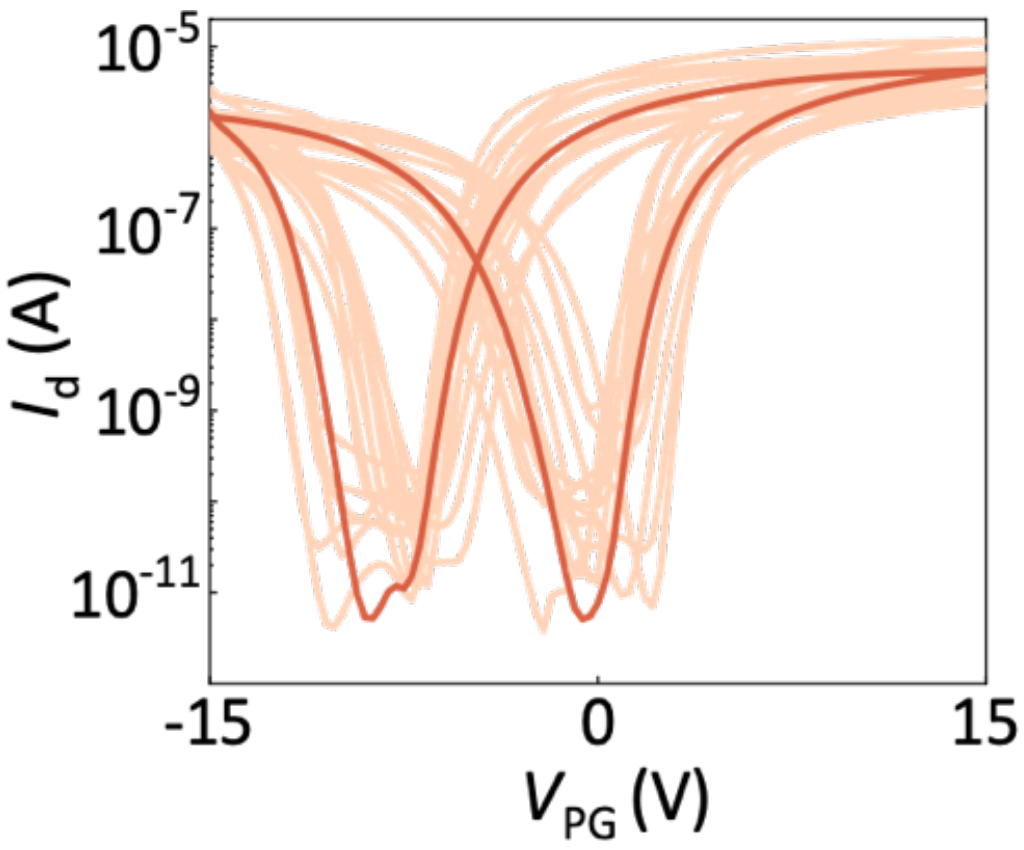

Supplementary Figure 5 Uniform transfer characteristics across multiple AHA-RFET devices. Statistical results of the transfer characteristics (I_d-V_{PG}) for 16 AHA-RFET devices, measured at V_{ds} = 0.1 V. All devices are fabricated under identical process conditions.

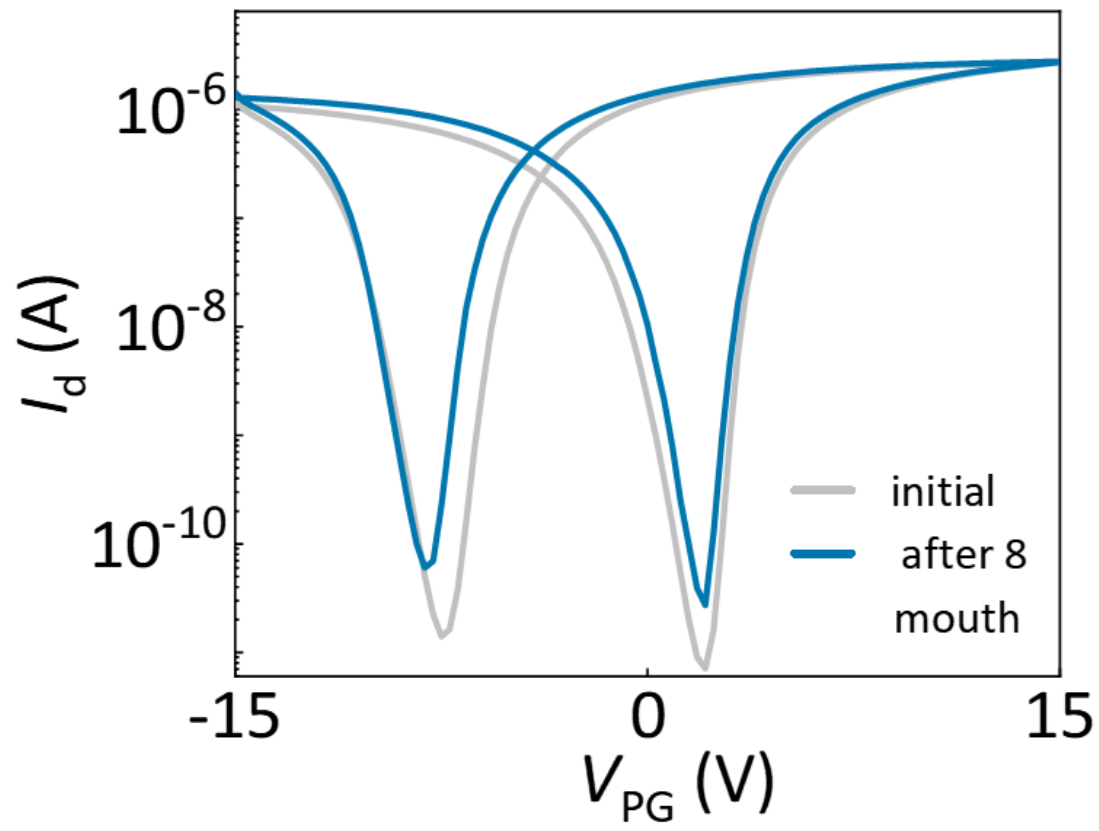

Supplementary Figure 6 Long-term stability and ambient reliability of the AHA-RFET. Comparison of transfer characteristics (I_d–V_{PG}) for a device measured initially and after 8 months of storage in ambient conditions.

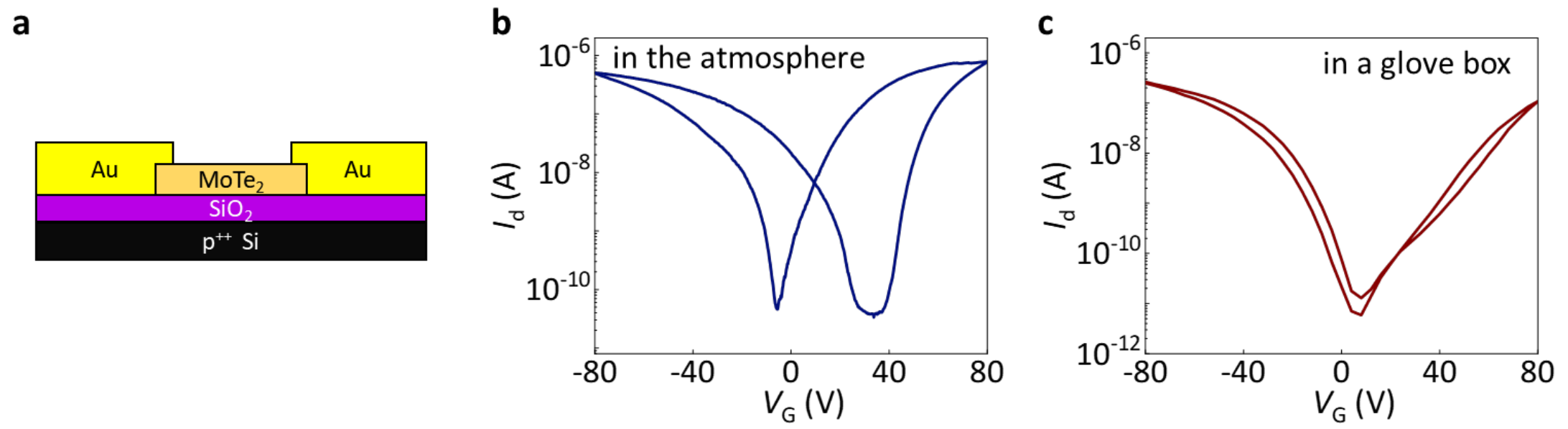

Supplementary Figure 7 Influence of exfoliation and fabrication environment on the hysteresis behavior of $MoTe_2$ FETs. a. Device structural schematics of $MoTe_2$ FET. **b.** The transfer characteristics (I_d–V_G) of a FET exfoliated and fabricated in ambient air, which exhibits significant hysteresis due to the adsorption of moisture and oxygen on the $MoTe_2$ surface. **c.** The transfer characteristics of a FET where both exfoliation and fabrication were completed within a nitrogen-filled glove box, demonstrating a high-quality and clean interface with virtually no hysteresis.

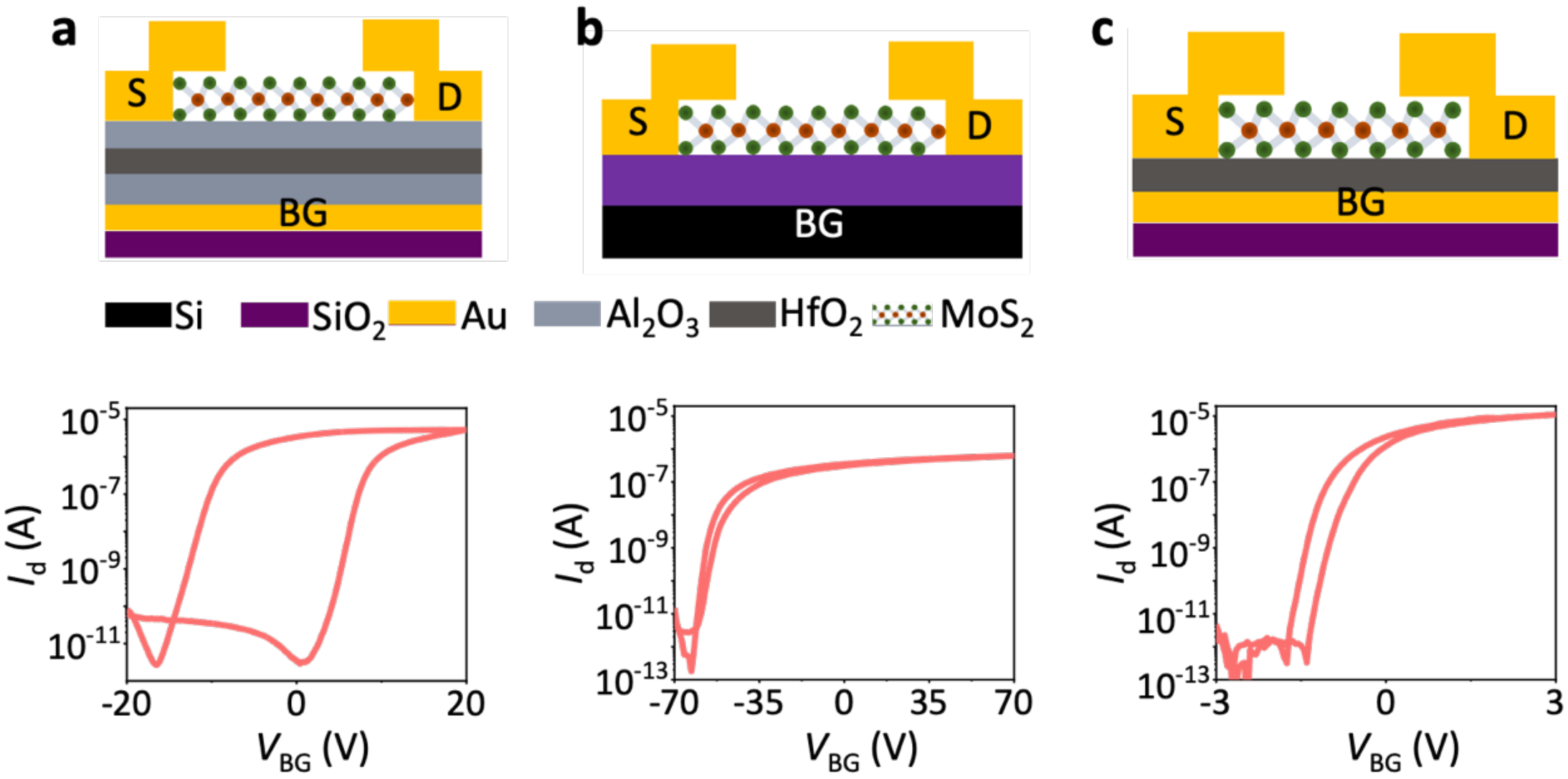

Supplementary Figure 8 Transfer characteristics of the fabricated device with different dielectric structures. The Schematic illustration and corresponding transfer characteristics (I_d-V_{BG}) of AHA (**a**), SiO_2 (**b**) or HfO_2 (**c**) based device, measured at V_{ds} = 0.1 V. No memory window is observed except in AHA-based devices.

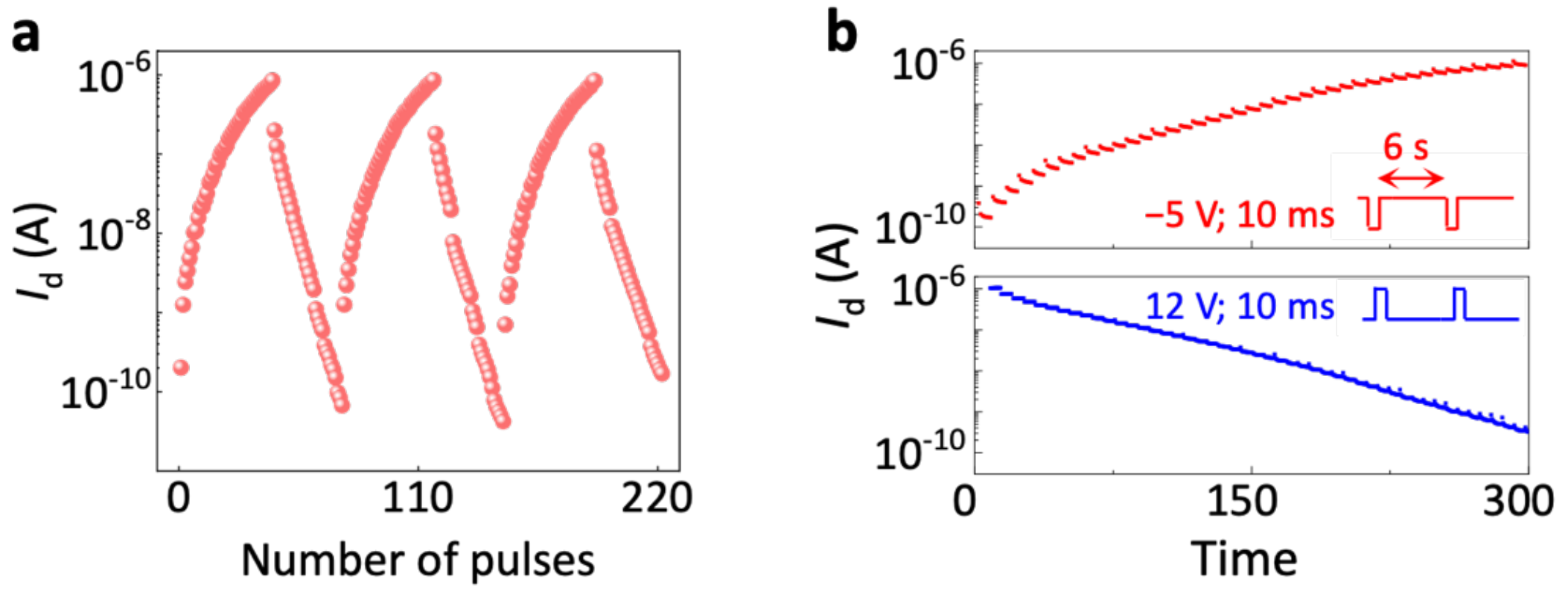

Supplementary Figure 9 Multilevel state programming of the AHA-RFET. a. Three cycles of multi-state switching under pulse voltage stimulation, measured at V_{ds} = 0.1 V. **b.** Multiple levels of cumulative currents with step increments. The pulse duration is 10 ms with a 6 s interval (inset).

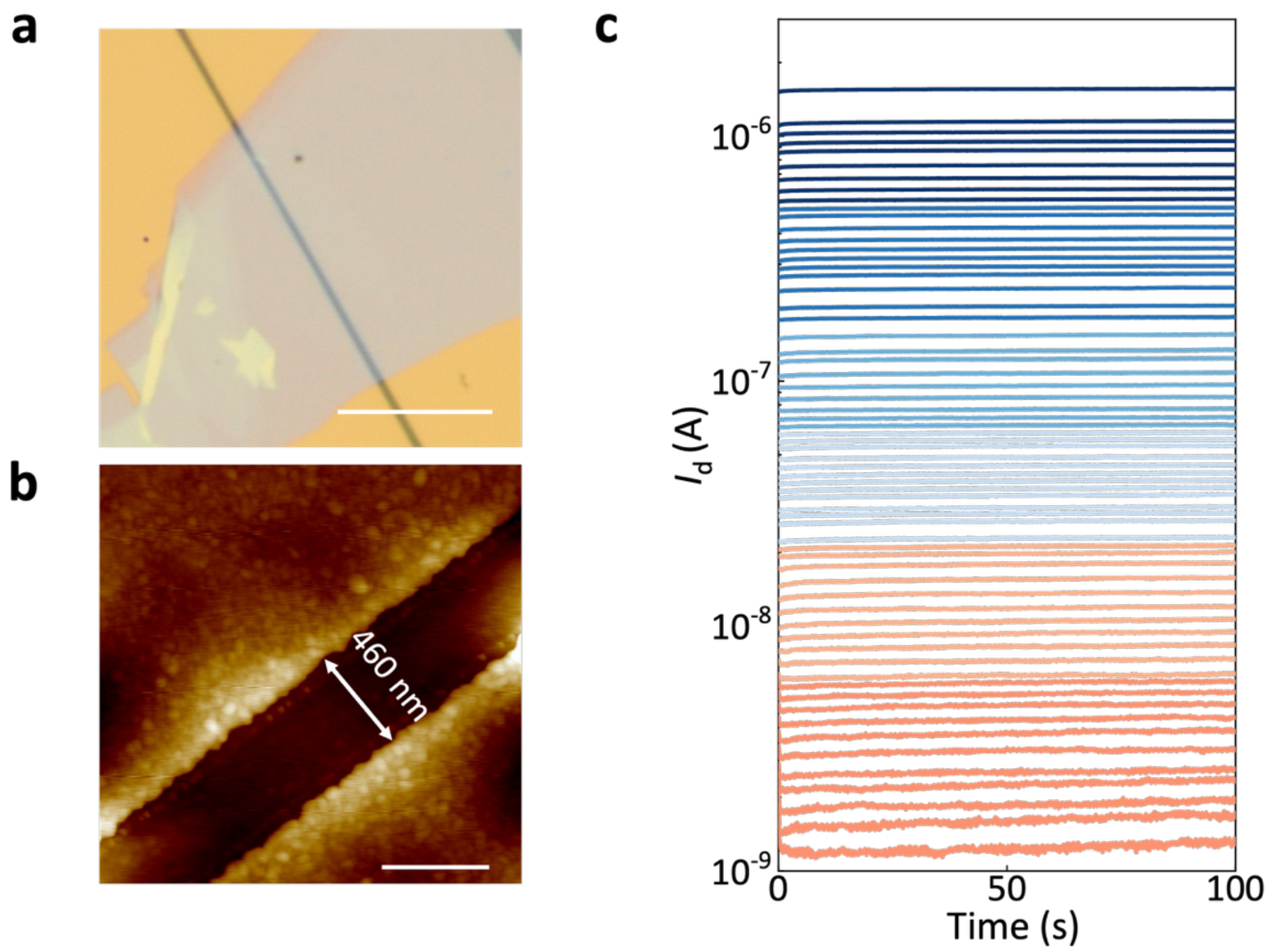

Supplementary Figure 10 Multi-level conductance characterization of a sub-micron AHA-RFET. a. Optical microscope image and **b.** AFM topography of the scaled device with a channel length of 460 nm. scale bar 10μm in a and 1 μm in b. **c.** Multi-state conductance data demonstrating stable 6-bit storage capability maintained over 100 s. The results confirm that the AHA stack facilitates high-precision charge control and trap uniformity even at the sub-micron scale, mitigating stochastic fluctuations typically encountered in reduced dimensions.

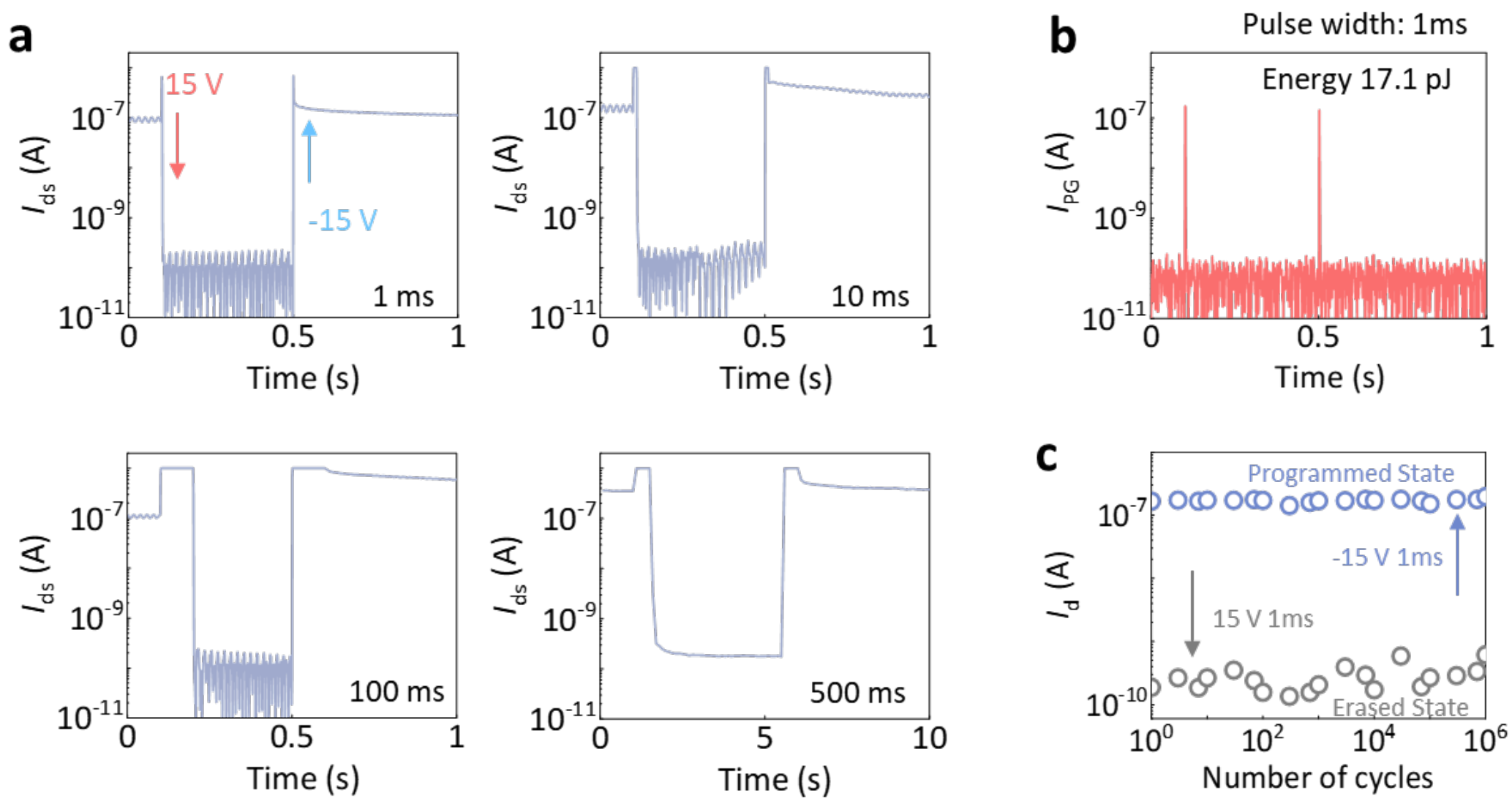

Supplementary Figure 11 Transient dynamic characterizations and reliability analysis. **a.** Transient drain current responses measured over time upon the application of alternating programming and erasing voltage, with widths varying from 1 ms to 500 ms. **b.** Transient programming gate current (I_{PG}) response during a 1 ms, +15 V pulse, with calculated operation energy consumption of 17.1 pJ. **c.** Cyclic endurance testing at the fastest accessible pulse configuration (1 ms, ± 15 V), maintaining a stable memory window over approximately 10^6 cycles before device failure.

Supplementary Table 1 The switching speed, operating voltage and endurance for various 2D-material-based charge trapping devices.

Functional layer	Voltages	Speed	Endurance	References
$Al_2O_3/HfO_2/hBN/WSe_2$	30 V	50 ns	10^7	[1]
$Al_2O_3/HfO_2/hBN/GR$	4.5 V	20 ns	5.5×10^6	[2]
$Al_2O_3/HfO_2/hBN/WSe_2$	14 V	1 ms	10^3	[3]
Al_2O_3/BP	20 V	100 ms	100	[4]
$Al_2O_3/HfO_2/Al_2O_3/BP$	18 V	20 ms	200	[5]
$Al_2O_3/HfO_2/Al_2O_3/MoS_2$	26 V	200 ms	120	[6]
$Al_2O_3/HfO_2/Al_2O_3/BP$	16 V	100 ms	120	[7]
$Al_2O_3/HfO_2/Al_2O_3/WSe_2$	40 V	100 ms	700	[8]
$Al_2O_3/HfO_2/Al_2O_3/MoS_2$	6 V	1 s		[9]
$Al_2O_3/HfO_2/Al_2O_3/MoS_2$	18 V	100 ms	200	[10]
$Al_2O_3/HfO_2/Al_2O_3/MoTe_2$	15 V	1 ms	4×10^4	This work

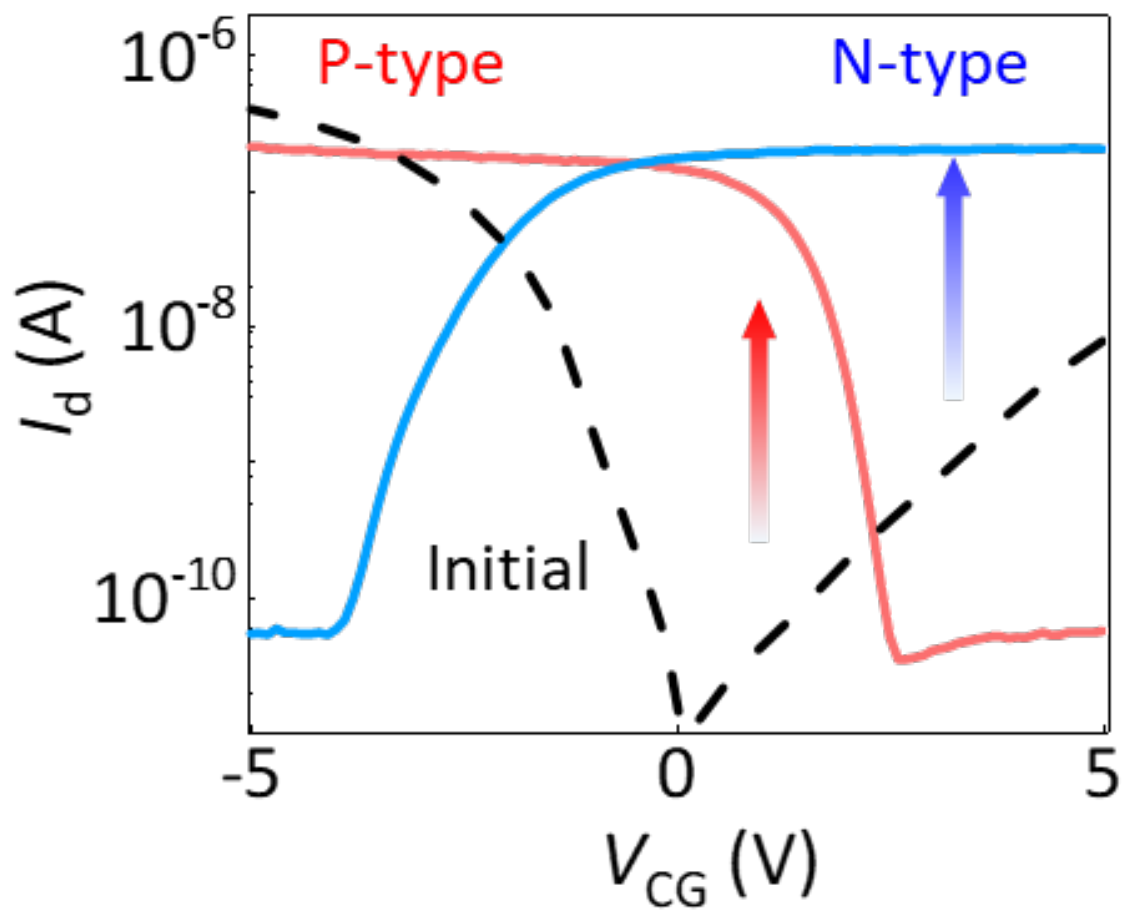

Supplementary Figure 12 The polarity switching of AHA-RFET. The transfer characteristics (I_d-V_{CG}) under CG controlled, the polarity transition shifts from bipolar to N-type FET mode or P-type FET mode, at V_{ds} = 0.1 V and V_{PG} = 0 V. Specifically, the N-type FET mode is achieved by applying a −15 V, 100 ms pulse on PG. The P-type FET mode is achieved by applying a 15 V, 1 s pulse on PG.

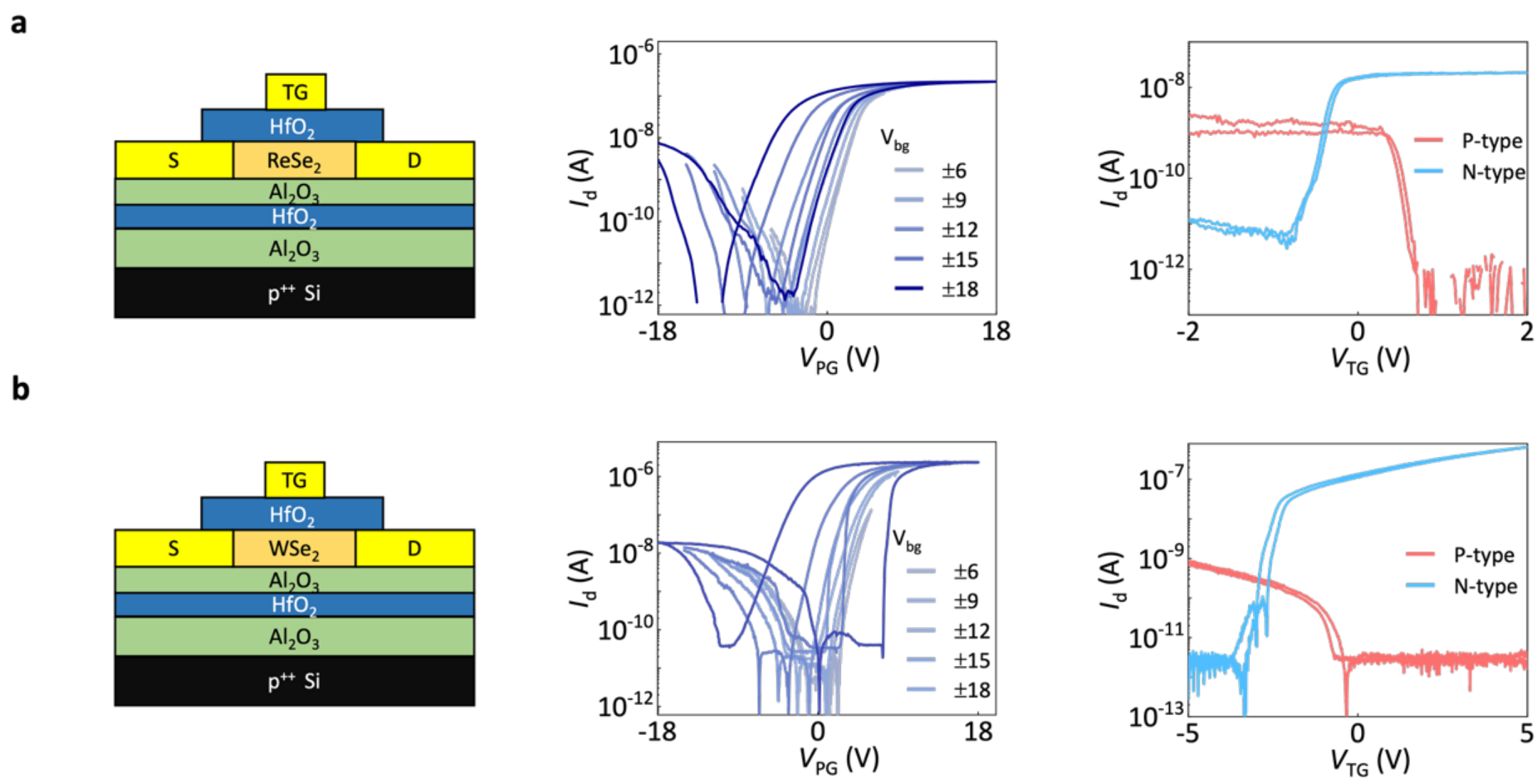

Supplementary Figure 13 Universal reconfigurability of the AHA-RFET across different 2D semiconductor channels. The characterization of AHA-FETs using $ReSe_2$ (**a**) and WSe_2 (**b**) as channel materials respectively. The device schematics show the top-gated architecture in which the AHA dielectric stack is integrated with different 2D semiconductors. Gate-tunable transfer characteristics (I_d–V_{PG}), measured at programming-gate voltages (V_{PG}) from 6 V to 18 V, show modulation of the memory window and carrier density in the tested materials. Polarity switching under dual-gate control shows p-type and n-type branches for both $ReSe_2$ and WSe_2.

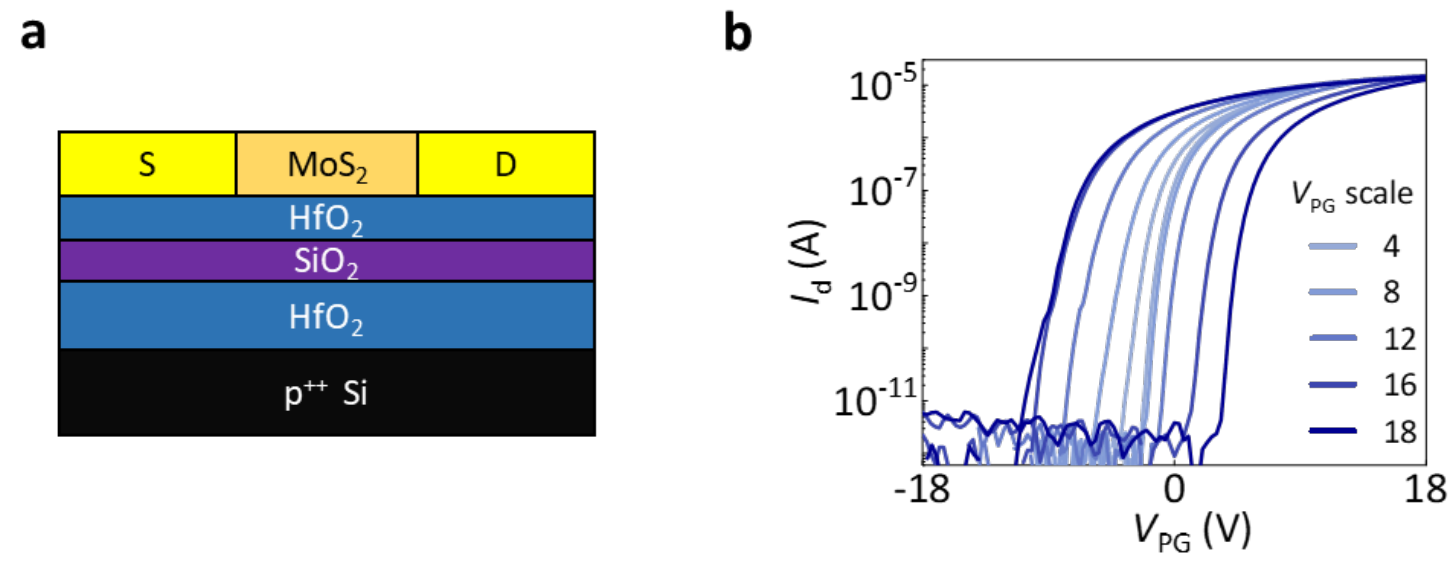

Supplementary Figure 14 Generality of the quasi-floating-gate mechanism with an alternative SHS dielectric configuration. a. Schematic illustration of the device architecture featuring a $SiO_2/HfO_2/SiO_2$ (SHS) dielectric stack integrated with the 2D channel. **b**. Transfer characteristics (I_d–V_{PG}) of $SiO_2/HfO_2/SiO_2$ (SHS) dielectric FET demonstrating a distinct and reliable non-volatile memory window.

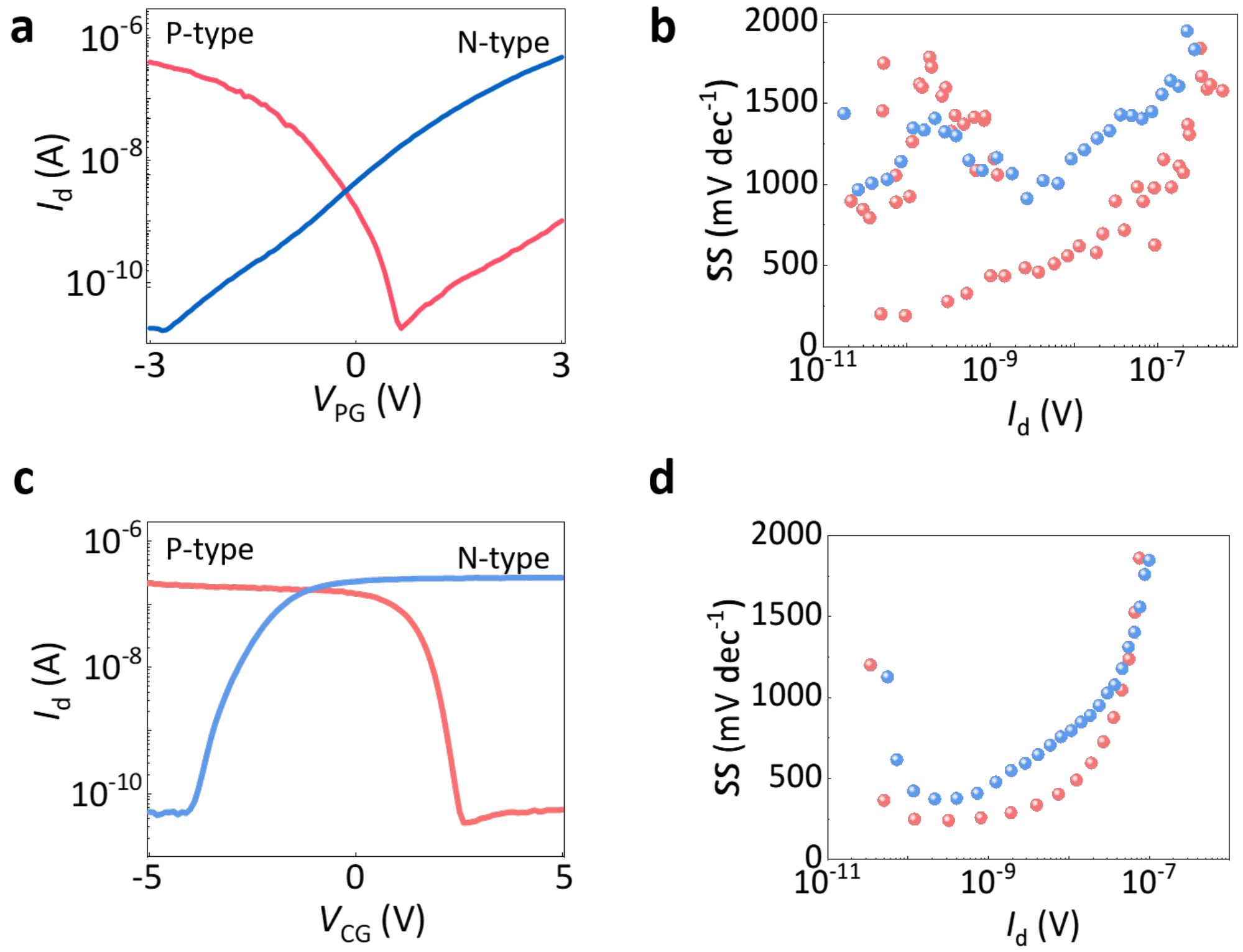

Supplementary Figure 15 Comparison of polarity switching modulated by PG and CG Control. a, c. The transfer characteristics under PG (**a**) and CG (**c**) controlled, the polarity transition shifts between N-type and P-type. Specifically, the N-type is achieved with a negative V_{PG} of −15 V, 100 ms. The P-type is achieved with a positive V_{PG} of 15 V, 1 s. **b, d.** Corresponding subthreshold swing under PG **(b)** and CG **(d)** controlled as a function of readout current. All data measured at V_{ds} = 0.1 V.

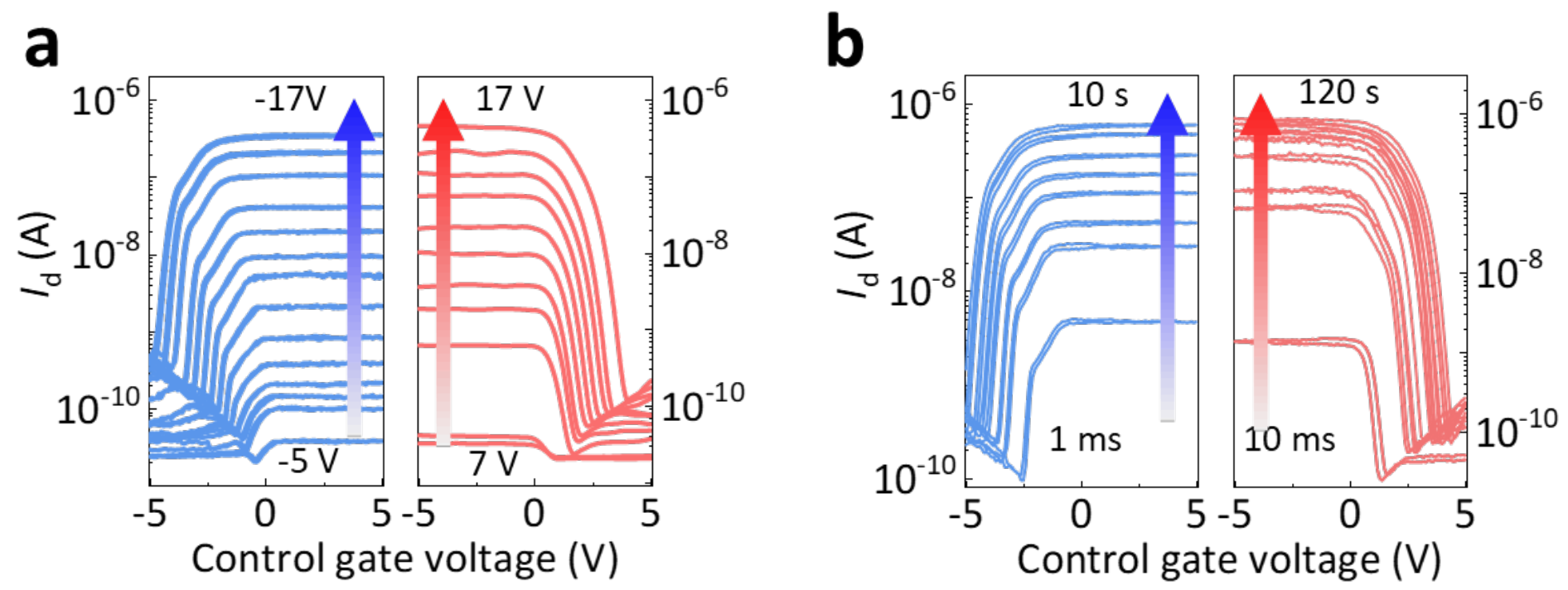

Supplementary Figure 16 Multiple-level modulation of the reconfigurability of AHA-RFET. a. b. Multiple-level modulation of the reconfigurability of the device enabled by (**a**) Pre-V_{bg}, varying from −17 to −5 V for N-type and 7 to 17 V for P-type, with a fixed pulse width (T_{PG}) of 100 ms for N-type and 1 s for P-type; (**b**) the pulse width of Pre-V_{bg}, varying from 1 ms to 10 s for N-type and 10 ms to 120 s for P-type, with a fixed V_{PG} amplitude of –15 V for N-type and 15 V for P-type. All data measured at V_{ds} = 0.1 V and V_{PG} = 0 V.

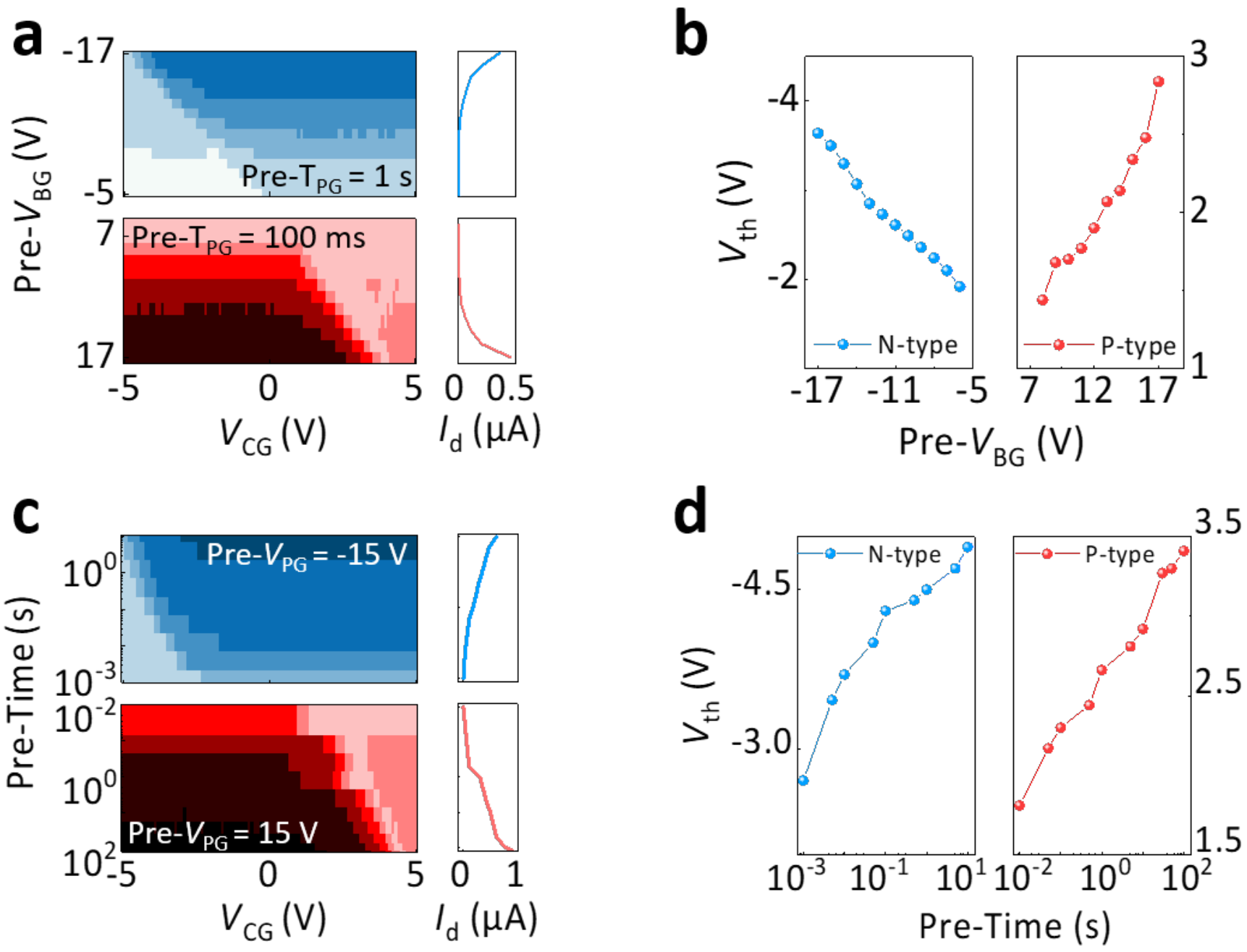

Supplementary Figure 17 Part of metrics evolution under different Pre-program conditions. a. I_d mapping as a function of V_{CG} under different V_{PG} amplitudes. **b.** Corresponding threshold voltage shift as a function of Pre-V_{PG} amplitude, with a fixed pulse width (Pre-T_{PG}) of 100 ms for N-type and 1 s for P-type. **c.** I_{ds} mapping as a function of V_{CG} under different Pre-T_{PG} values. **d.** Corresponding threshold voltage shift as a function of Pre-T_{PG} pulse width, with a fixed Pre-V_{PG} amplitude of –15 V for N-type and 15 V for P-type. All mappings are measured at V_{ds}= 0.1 V. On-state I_d line profiles are displayed on the right of **a** and **d**.

a

parameter	$MoTe_2$	HfO_2	Al_2O_3	SiO_2
T (nm)	6.5 (channel)	10 (Top)/8 (Bottom)	8(interlayer)/24 (base)	3 (seed)
Eg (eV)	1.0	4.9	7.7	9.0
χ	3.85[1]	2.5	1.0	0.9
ε	7[2]	22	9	3.9
m_e	0.58[3]			
m_h	0.78[3]			

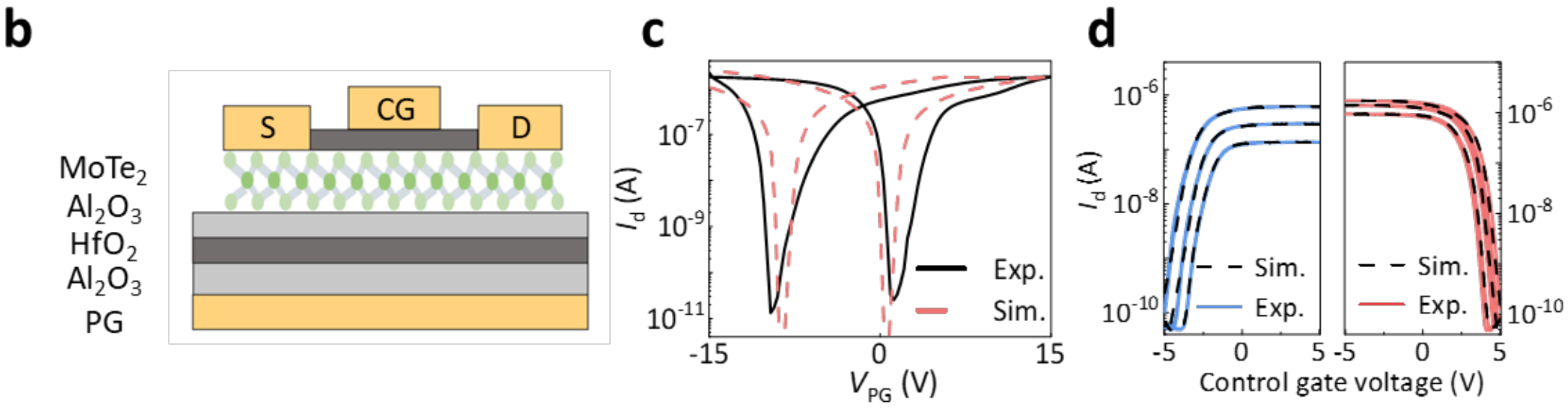

Supplementary Figure 18 TCAD simulations of AHA-RFET. a. The detailed parameters used for simulations. Here, T is the material thickness, E_g is the bandgap, ε is the relative dielectric constant, χ is the electron affinity and m_e/m_h is the electron/hole effective mass. **b**. The Schematic of AHA-RFET used in simulation. **c**. Experimental verification of simulation approach: transfer characteristics (I_d-V_{PG}) of AHA-RFET simulation and experimental results at V_{ds} = 0.1 V. **d**. Experimental verification of simulation approach: transfer characteristics (I_d-V_{CG}) of AHA-RFET simulation and experimental results at V_{ds} = 0.1 V and V_{PG} = 0 V.

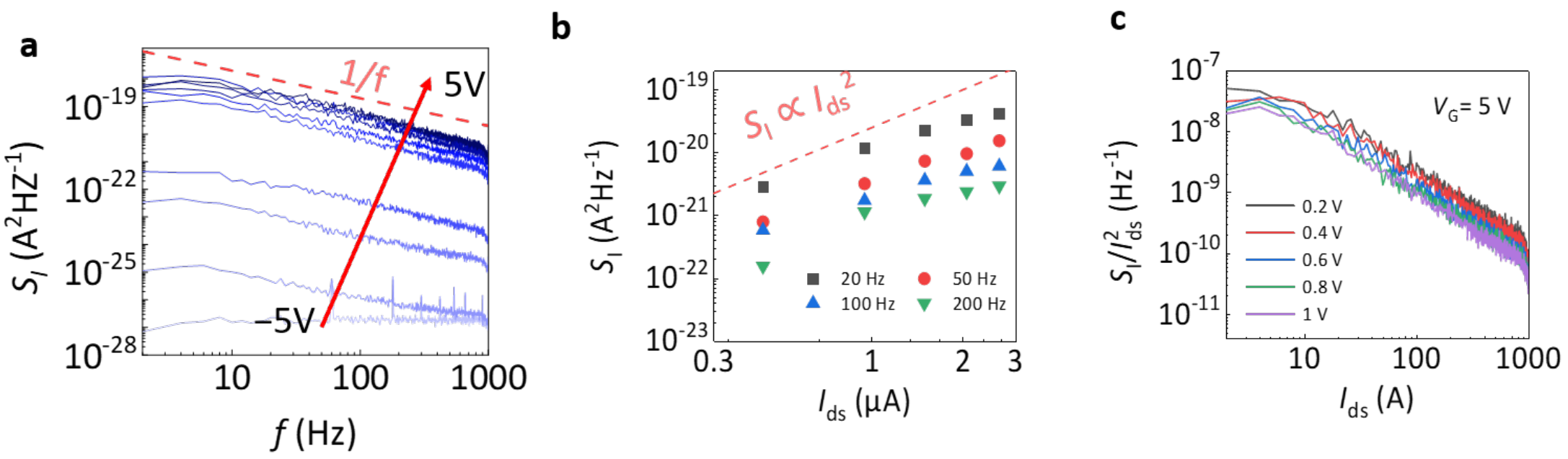

Supplementary Figure 19 Low-frequency noise analysis of the AHA-RFET. a. Noise power spectral density (S_I) as a function of frequency (f) under various gate voltages, exhibiting a clear $1/f$ dependence. **b.** S_I as a function of drain current (I_{ds}) at different frequencies; the dashed line represents the $S_I \propto I_{ds}^2$ relationship, characteristic of resistance fluctuations. **c.** Normalized noise (S_I/I_{ds}^2) versus I_{ds} at various V_{ds} values. The overlapping curves indicate that the LFN originates from the conductive channel rather than contact barriers, while the significant decrease with increasing current points to the dominance of surface-trapping events at the dielectric/channel interface.

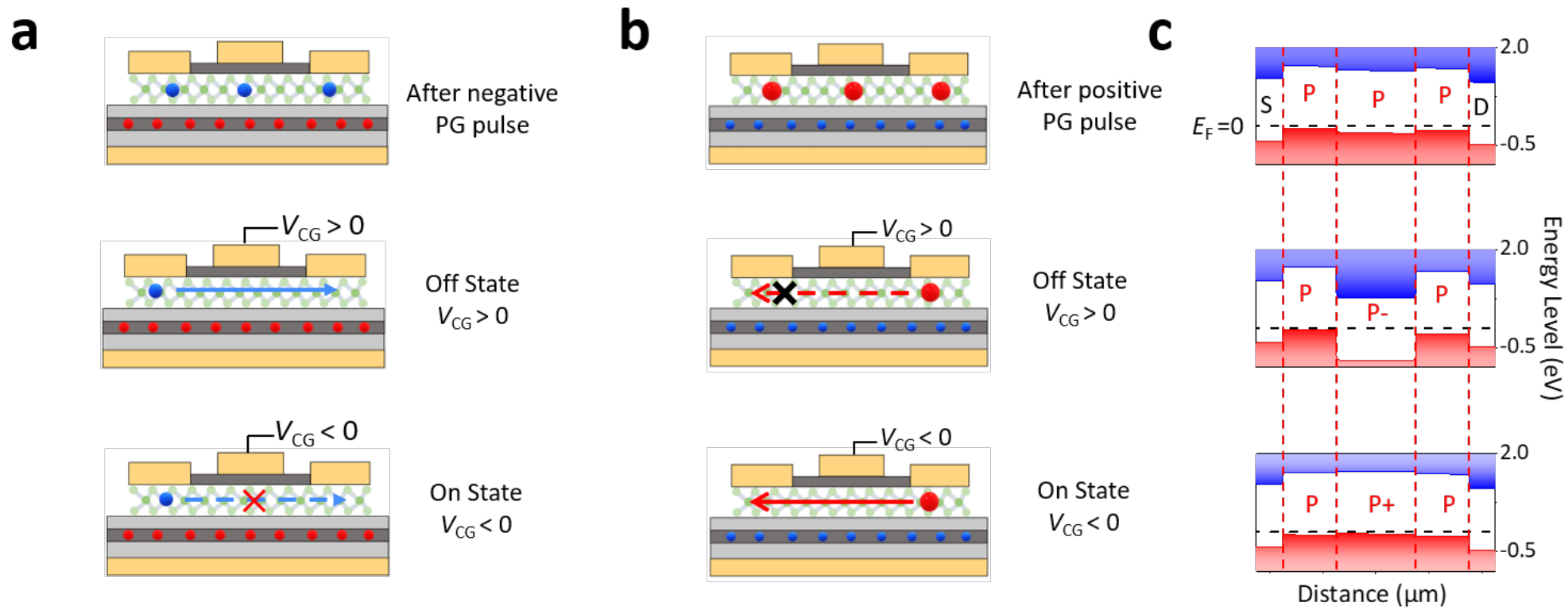

Supplementary Figure 20 Variation in channel under CG controlled in P-type and N-type FET mode. a. Schematic illustration of AHA-RFET under different CG voltage. AHA-RFET is configured as N-type FET mode. **b. c.** (b) Schematic illustration of AHA-RFET and (c) corresponding simulated energy band diagrams at drain, $MoTe_2$ and source region under CG voltage. AHA-RFET is configured as P-type FET mode.

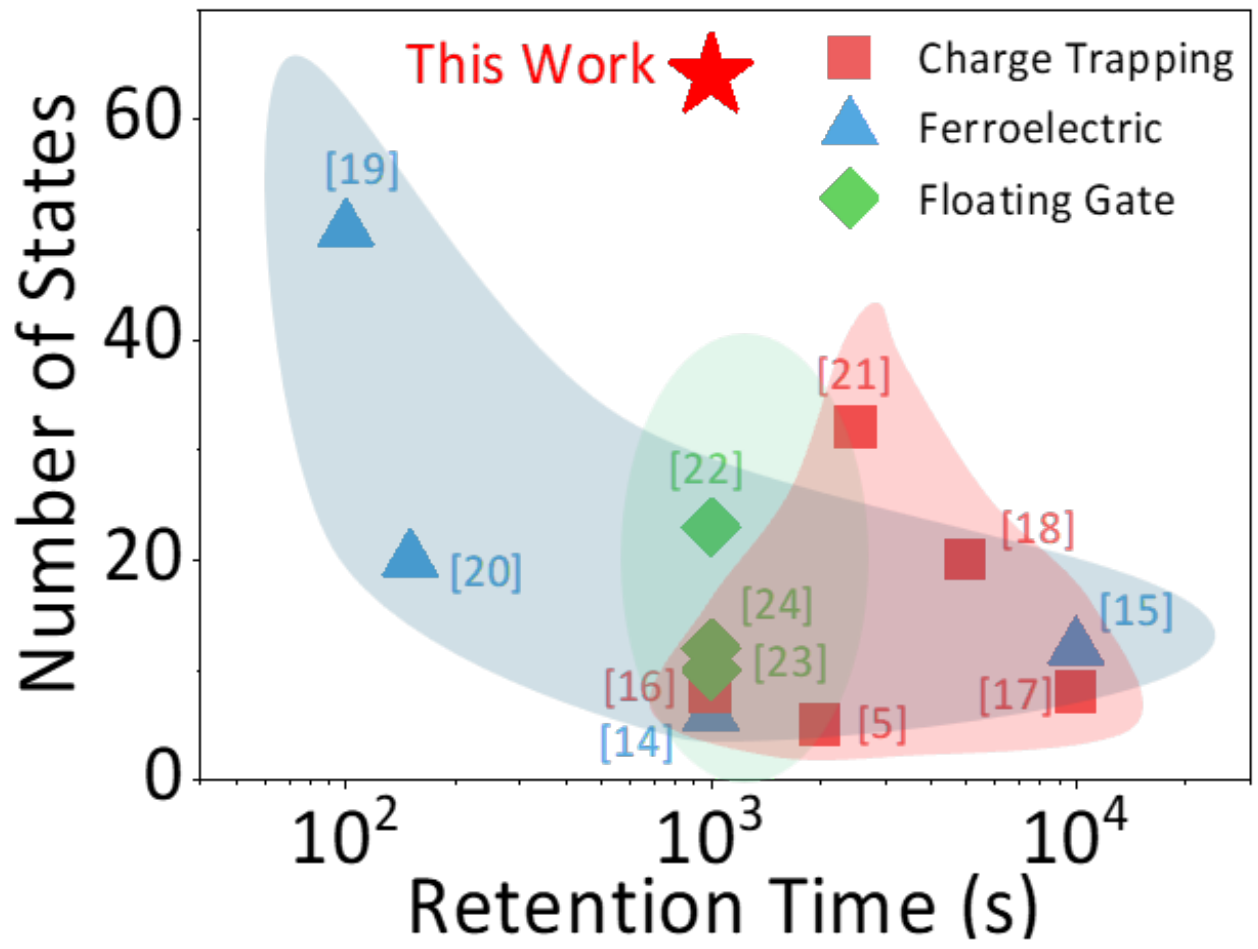

Supplementary Figure 21 Performance benchmark for reconfigurable and nonvolatile devices based on different mechanisms. Reconfigurable and nonvolatile devices include reconfigurable diodes, reconfigurable field-effect transistors, AHA-based memories, and ferroelectric transistors.

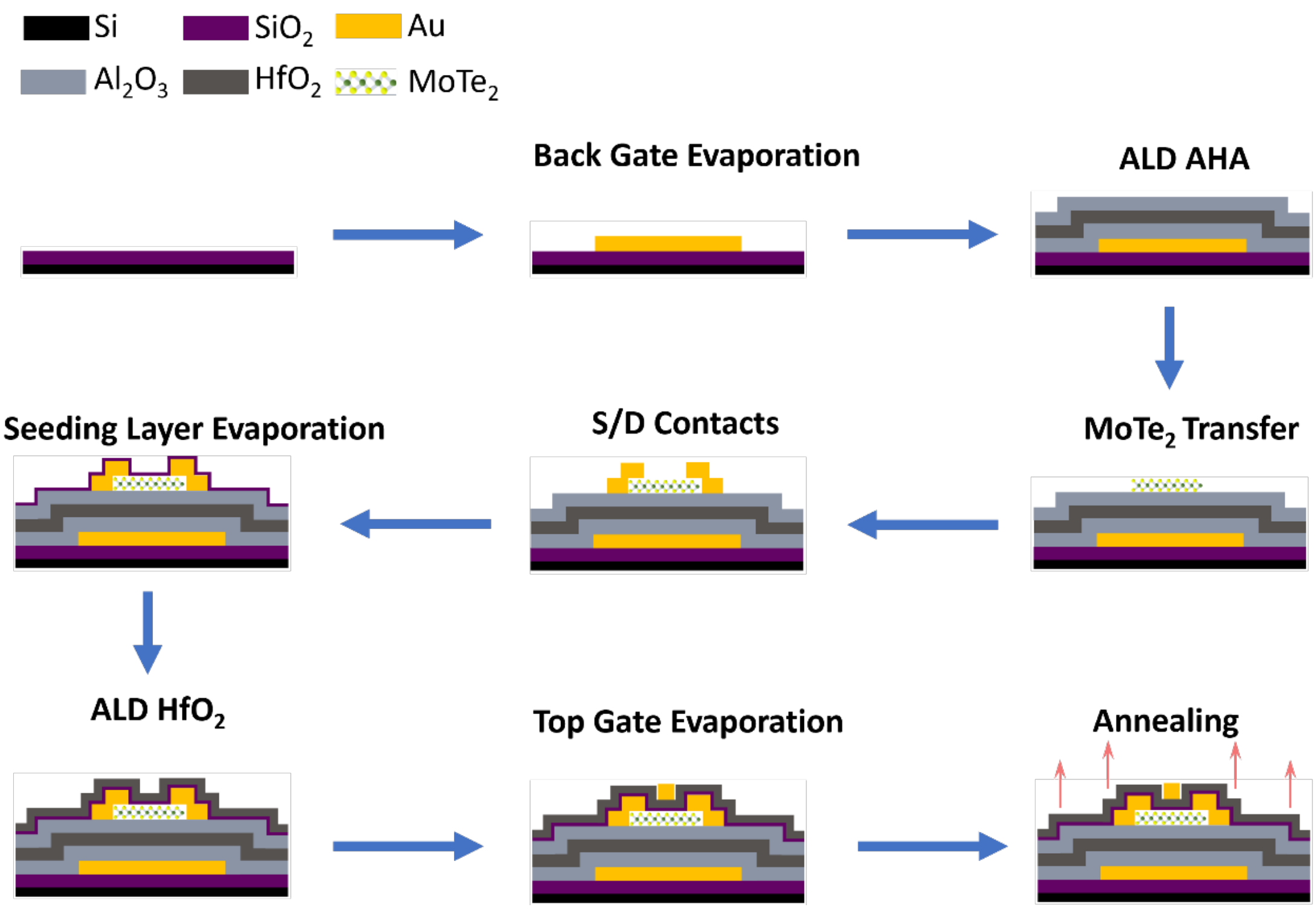

Supplementary Figure 22 Fabrication process flow of the AHA-RFET array. This all-active architecture relies on three key innovations—isolated back-gate arrays enabling individual polarity control; a low-temperature ALD-grown AHA stack and high-quality top-gate dielectric maximizing dual-gate modulation; and precisely aligned interconnects ensuring full utilization of reconfigurable characteristics.

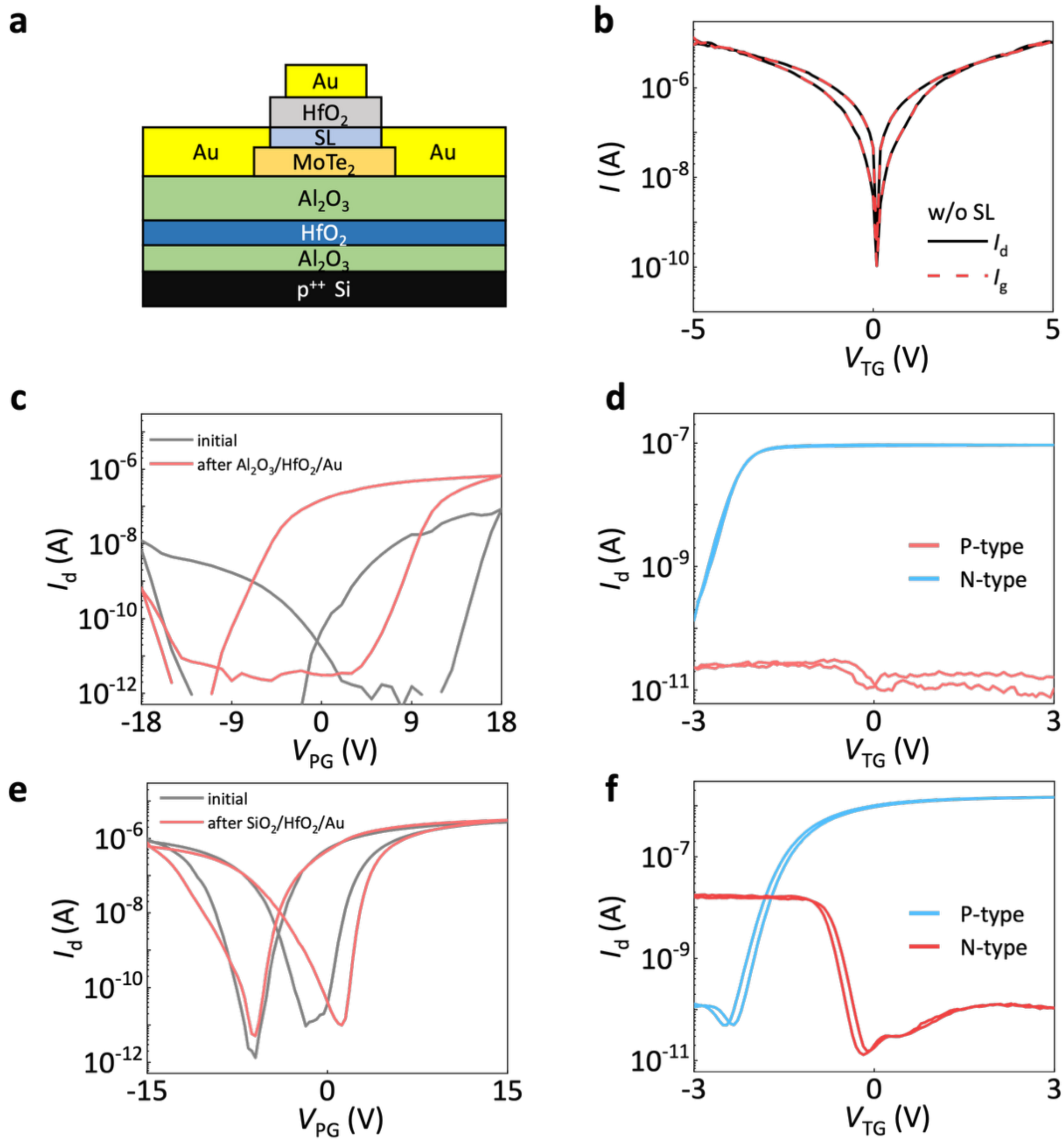

Supplementary Figure 23 Role of the seeding layer (SL) on top-gate dielectric growth and device performance. a. Device structural schematic illustrating the integration of the seeding layer between the channel and the top dielectric. **b.** Characterization of a top-gate AHA-RFET fabricated without SL. The overlap of I_d and I_g curves ($I_d = I_g$) demonstrates that the top-gate dielectric layer lacks essential dielectric isolation capability. **c, d.** Performance of a double-gate AHA-RFET with an Al_2O_3 SL. (c) Comparison of programming gate transfer characteristics before and after the seeding layer deposition. (d) The device exhibits standard top-gate transfer performance. Both suffer from severe n-type doping. **e, f.** Performance of a double-gate AHA-RFET with a SiO_2 SL. (e) Programming gate (PG) and (f) control gate transfer characteristics

show standard reconfigurable transistor performance, confirming that SiO_2 is an ideal SL for assisting HfO_2 growth while preserving the intrinsic bipolarity of the device.

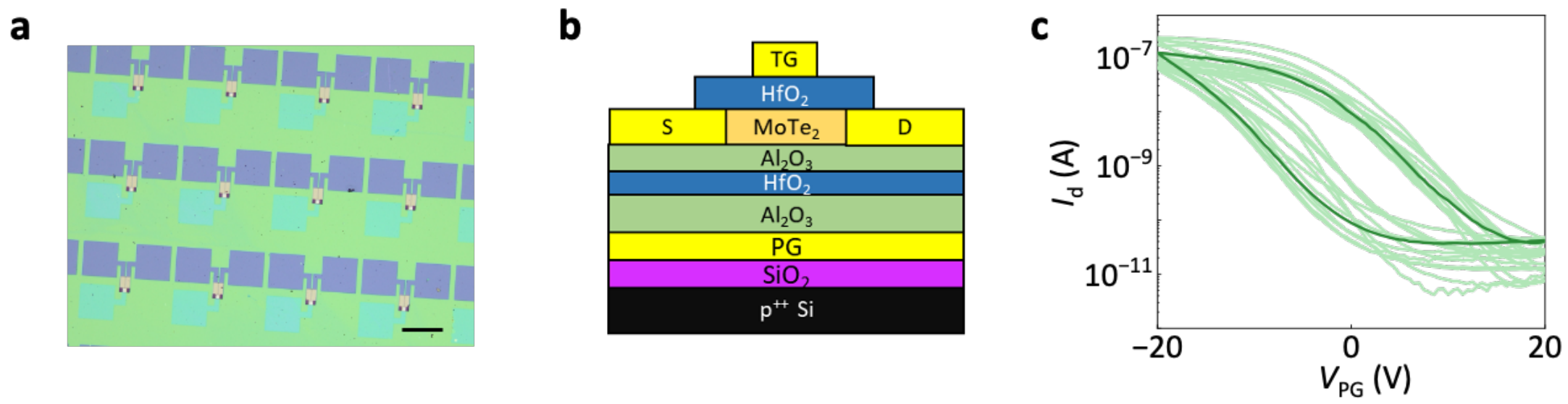

Supplementary Figure 24 Evaluation of integration potential and uniformity using large-area synthesized $MoTe_2$. a. Optical microscope image of an AHA-RFET array fabricated using CVD-grown $MoTe_2$, with a scale bar of 200 μm. **b.** Schematic illustration of the device architecture. **c.** Transfer characteristics (I_d–V_{PG}) of multiple devices within the array, demonstrating high fabrication yield and excellent consistency across the large-area synthesized 2D Material.

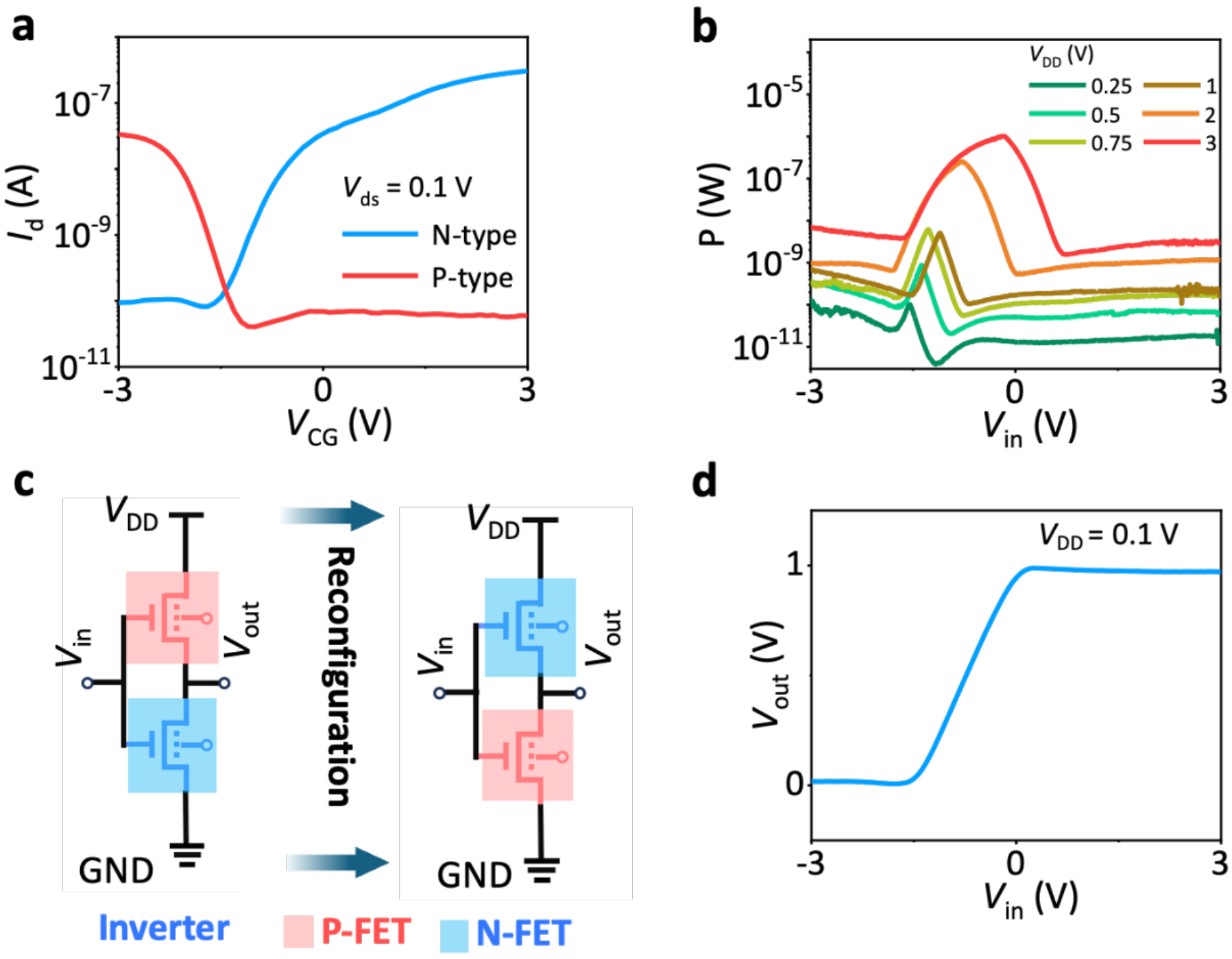

Supplementary Figure 25 Inverter circuit reconfiguration and performance. a. Transfer characteristics (I_d-V_{CG}) of AHA-RFETs in the inverter after reconfiguration. **b.** Power consumption of the inverter circuit as a function of the input voltage, V_{DD} varying from 0.25 to 3.0 V. **c, d.** Schematic illustration of circuit mode switching (**c**) by applying inverted programming polarities (−15 V, 100 ms for the pull-up transistor and 15 V, 1 s for the pull-down transistor) and corresponding voltage transfer characteristics (**d**).

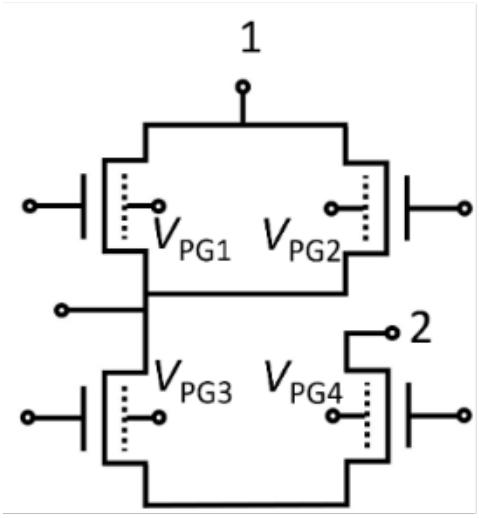

1	2	Pre-V_{PG1}	Pre-V_{PG2}	Pre-V_{PG3}	Pre-V_{PG4}	Function
V_{DD}	GND	15	15	−15	−15	NAND
V_{DD}	GND	−15	15	−15	15	RIMP
V_{DD}	GND	15	−15	15	−15	IMP
V_{DD}	GND	−15	−15	15	15	OR
GND	V_{DD}	15	15	−15	−15	AND
GND	V_{DD}	−15	15	−15	15	NIMP
GND	V_{DD}	15	−15	15	−15	RNIMP
GND	V_{DD}	−15	−15	15	15	NOR

Supplementary Figure 26 Multifunctional switching of 4T reconfigurable logic circuit. The schematic of 4T reconfigurable logic circuit and corresponding function via different configurable operation.

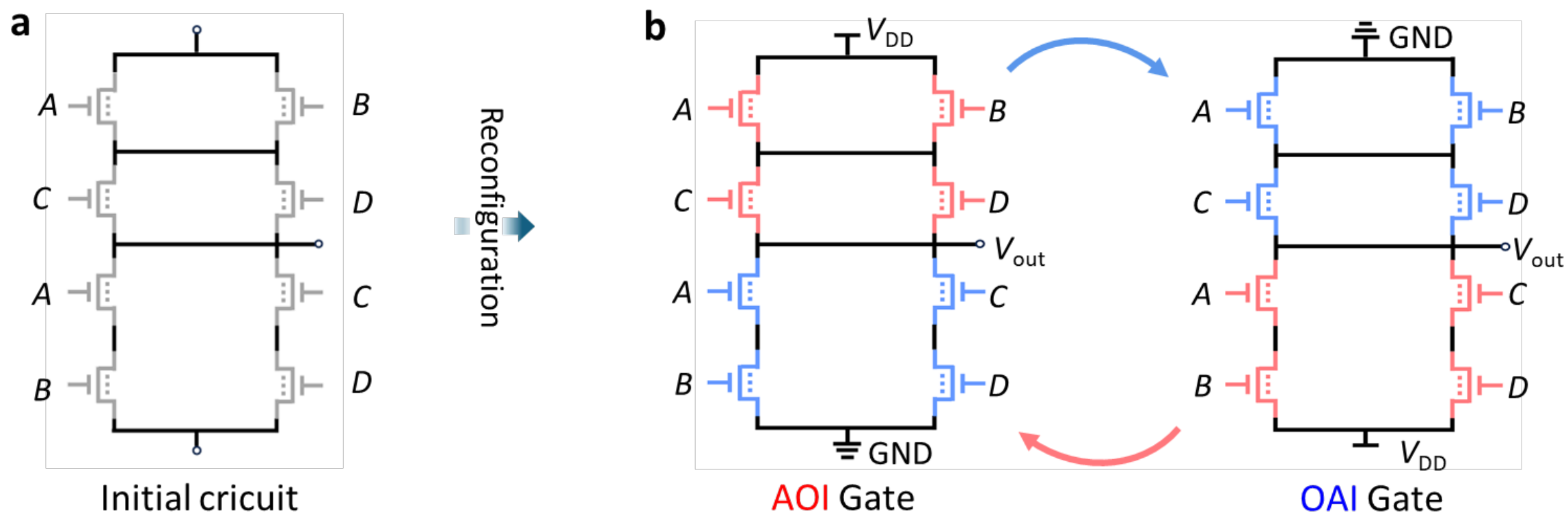

Supplementary Figure 27 Reconfiguration schematic of the 8T reconfigurable gate. The circuit reconfigures from its initial state to AOI Gate and OAI Gate. Programming with PG voltage pulse of −15 V, 100 ms for N-type FET mode and 15 V, 1 s for P-type FET mode.

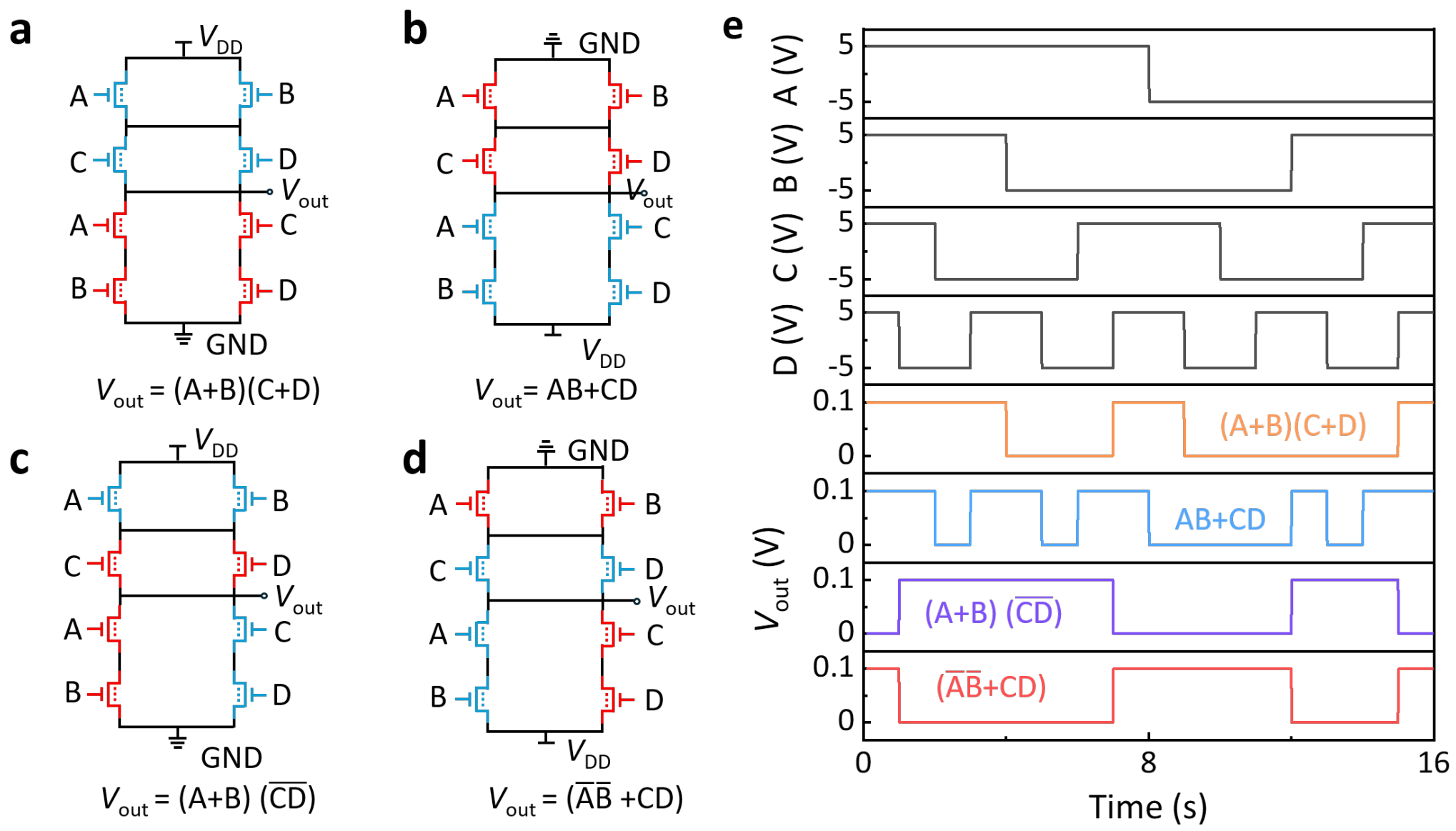

Supplementary Figure 28 Extended Logic Functions of the 8T reconfigurable gate. The Schematic illustration **(a)** of four extended logic functions of the 8T reconfigurable gate and corresponding simulated waveforms **(b)**.

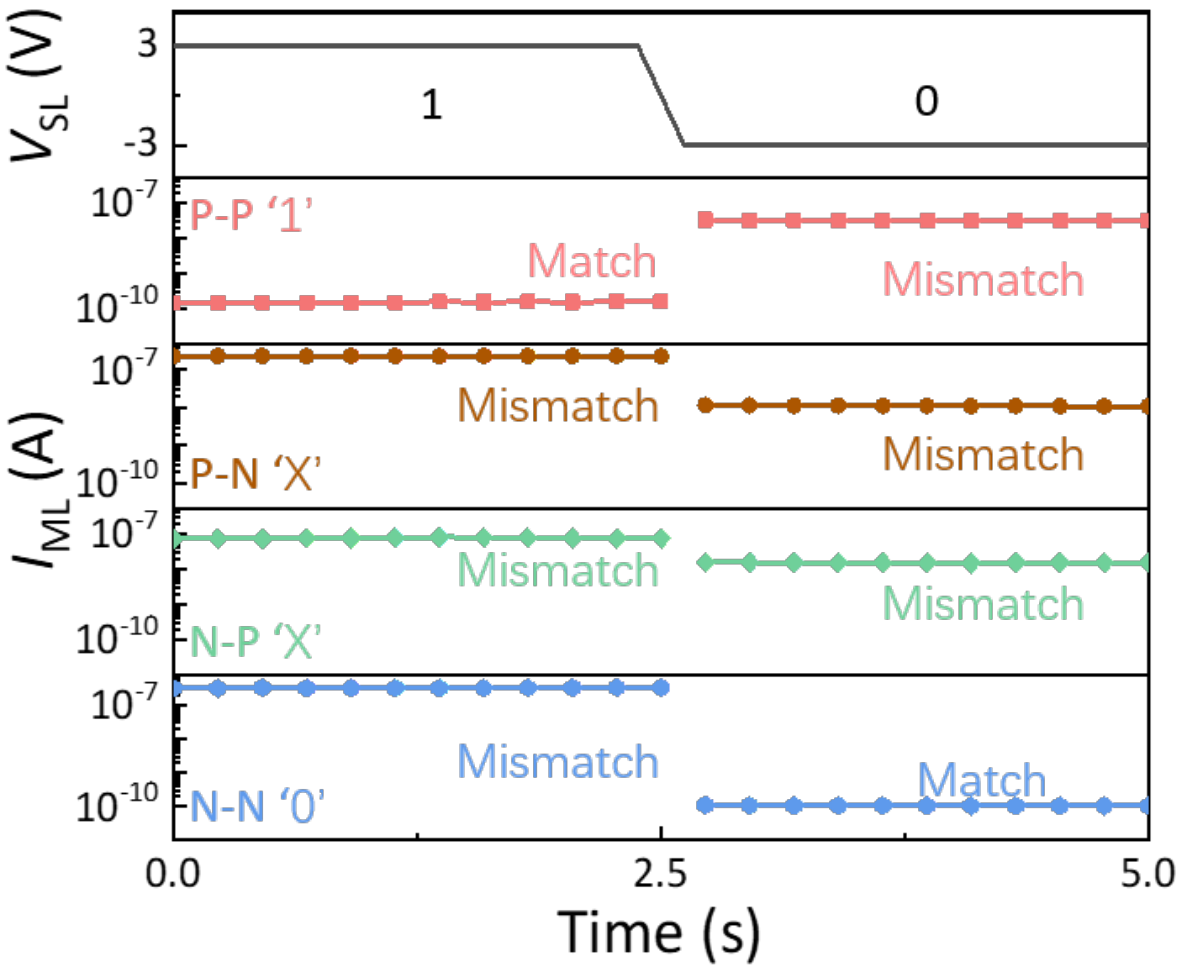

Supplementary Figure 29 Experimental results of NOR TCAM. NOR TCAM cell consisting of two AHA-RFETs, measured at V_{ML} = 0.1 V. Two AHA-RFETs are programmed in P-P, P-N, N-P, N-N states, respectively.

Supplementary Table 2 Performance comparison of search speed and cell composition among various reported CAM/TCAM architectures

Kind	Composition	Speed	References
CAM	2T	500 μs	[25]
CAM	2C	10 μs	[26]
TCAM	2T	100 μs	[27]
TCAM	2C	5 μs	[28]
TCAM	2T	5 μs	[29]
TCAM	2T1R	100 μs	[30]
TCAM	2T1R	500 ns	[31]
TCAM	1M	100 ms	[32]
TCAM	1C1N	17.5 μs	[33]
TCAM	2T	10 μs	This Work

Supplementary Notes

Supplementary Note 1. Details of merits in Figure 2f

The on-state current, off-state current, and on/off ratio—parameters exhibiting exponential variation—were evaluated after logarithmic normalization to enable a comparative analysis, using

$$\text{Normalized}_{\log} = \frac{\log_{10} x}{\log_{10} x_{\max}}.$$

The calculation procedure is as follows:

**merits**	**N-type**		**P-type**	
	**initial**	**normalization**	**initial**	**normalization**
SS (mV dec^{-1})	268	1	248	0.92
Retention (s)	10^4	1	10^4	1
I_{on} (A)	5.943×10^{-7}	0.998	6×10^{-7}	1
I_{off} (A)	5.9×10^{-10}	1	2.58×10^{-10}	0.966
I_{on}/I_{off}	1007	0.88	2557	1
$\lvert V_{th}\rvert$ (V)	4.5	1	3.27	0.72
Endurance	3×10^5	1	3×10^5	1

Supplementary References

1. Huang X, Liu C, Tang Z, et al. An ultrafast bipolar flash memory for self-activated in-memory computing. *Nat. Nanotechnol.* **18**, 486-492 (2023).
2. Xiang Y, Wang C, Liu C, et al. Subnanosecond flash memory enabled by 2D-enhanced hot-carrier injection. *Nature* **641**, 90-97 (2025).
3. Yang E, Ma J, Lee C, et al. Large-Scale Dual-Channel WSe_2 Reconfigurable Field-Effect Transistors with Charge-Trapping Layer for 2T TCAM and Reconfigurable Logic. *Nano Lett*. **26**, 2089-2097 (2026).
4. Tian H, Deng B, Chin M L, et al. A dynamically reconfigurable ambipolar black phosphorus memory device. *ACS Nano* **10**, 10428-10435 (2016).
5. Lee S, Peng R, Wu C, et al. Programmable black phosphorus image sensor for broadband optoelectronic edge computing. *Nat. Commun*. **13**, 1485 (2022).
6. Zhang E, Wang W, Zhang C, et al. Tunable charge-trap memory based on few-layer MoS_2. *ACS Nano* **9**, 612-619 (2015).
7. Feng Q, Yan F, Luo W, et al. Charge trap memory based on few-layer black phosphorus. *Nanoscale* **8**, 2686-2692 (2016).
8. Liu C, Yan X, Wang J, et al. Eliminating overerase behavior by designing energy band in high-speed charge-trap memory based on WSe_2. *Small* **13**, 1604128 (2017).
9. Li J, Zhang H, Ding Y, et al. A non-volatile AND gate based on Al_2O_3/HfO_2/Al_2O_3 charge-trap stack for in-situ storage applications. *Sci. Bull.* **64**, 1518-1524 (2019).
10. Hou X, Yan X, Liu C, et al. Operation mode switchable charge-trap memory based on few-layer MoS_2. *Sci. Technol.* **33**, 034001 (2018).
11. Park Y J, Katiyar A K, Hoang A T, et al. Controllable P- and N-Type Conversion of $MoTe_2$ via Oxide Interfacial Layer for Logic Circuits. *Small* **15**, 1901772 (2019).
12. Liu F, Wang J & Guo H. Atomistic Simulations of Device Physics in Monolayer Transition Metal Dichalcogenide Tunneling Transistors. *IEEE Trans. Electron Devices* **63**, 311-317 (2016).
13. Cheng L, Liu Y. What Limits the Intrinsic Mobility of Electrons and Holes in Two Dimensional Metal Dichalcogenides? *J. Am. Chem. Soc.* **140**, 17895-17900 (2018).
14. Zhou Y, Wang Y, Zhuge F, et al. A reconfigurable two-WSe_2-transistor synaptic cell for

reinforcement learning. *Adv. Mater*. **34**, 2107754 (2022).

15. Nazzari D, Wind L, Sistani M, et al. Nonvolatile Reconfigurable Transistor via Ferroelectrically Induced Current Modulation. *ACS Appl. Mater. Interfaces* **17**, 10784-10791 (2025).
16. Zhou Y, Fu J, Chen Z, et al. Computational event-driven vision sensors for in-sensor spiking neural networks. *Nat. Electron.* **6,** 870-878 (2023).
17. Lee M P, Gao C, Tsai M Y, et al. Silicon–van der Waals heterointegration for CMOS-compatible logic-in-memory design. *Sci. Adv.* **9**, eadk1597 (2023).
18. Tsai M Y, Huang C T, Lin C Y, et al. A reconfigurable transistor and memory based on a two-dimensional heterostructure and photoinduced trapping. *Nat. Electron.* **6**, 755-764 (2023).
19. Wu G, Zhang X, Feng G, et al. Ferroelectric-defined reconfigurable homojunctions for in-memory sensing and computing. *Nat. Mater.* **22**, 1499-1506 (2023).
20. Dang Z, Guo F, Wang Z, et al. Object motion detection enabled by reconfigurable neuromorphic vision sensor under ferroelectric modulation. *ACS Nano* **18**, 27727-27737 (2024).
21. Ying H, Xie K, Cai H, et al. Multifunctional Logic-in-Memory Circuits Based on Reconfigurable WSe_2 Transistors via Enhanced Ambipolarity. *ACS Nano* **19**, 19442-19453 (2025).
22. Jiang Y, Liu C, Cao Z, et al. A scalable integration process for ultrafast two-dimensional flash memory. *Nat. Electron.* **7**, 868-875 (2024).

23. Li W, Mu T, Sun Z, et al. Reconfigurable Multifunctional Semifloating Gate Transistors Based on the $ReSe_2$/h-BN/Graphene van der Waals Heterostructure. *ACS Appl. Mater. Interfaces* **17**, 18623-18635 (2025).

24. Zhu Y, Wang Y, Pang X, et al. Non-volatile 2D MoS_2/black phosphorus heterojunction photodiodes in the near-to mid-infrared region. *Nat. Commun.* **15**, 6015 (2024).

25. Gao G, Wen B, Yang N, et al. Sb-contacted MoS_2 flash memory for analogue in-memory searches. *Nat. Nanotechnol.* **21**, 198-206 (2026).

26. Zhou Z, Zhong H, Jiao L, et al. Charge-domain content addressable memory based on ferroelectric capacitive memory for reliable and energy-efficient one-shot learning. *Nat. Commun.* **16**, 8047 (2025).

27. Ni K, Yin X, Laguna A F, et al. Ferroelectric ternary content-addressable memory for one-shot learning. *Nat. Electron.* **2**, 521-529 (2019).

28. Hwang H, Yu J, Youn S, et al. 2C-Ternary Content Addressable Memory in Memcapacitor Crossbar Array with NAND Flash Structure. *Small* **21**, 2408618 (2025).

29. Phadke O, Hsu P K, Luo Y C, et al. Ternary content addressable memory with ferroelectric non-volatile capacitor. *IEEE Electron Device Lett.* **46**, 872-875 (2025).

30. Sun C, Han K, Samanta S, et al. Highly scaled InGaZnO ferroelectric field-effect transistors and ternary content-addressable memory. *IEEE Trans. Electron Devices* **69**, 5262-5269 (2022).

31. Verreck, D, Arreghini, A, Schanovsky F. et al. Understanding the ISPP slope in charge trap flash memory and its impact on 3-D NAND scaling. In *Proc. 2021 IEEE Int. Electron Devices Meet. (IEDM)* 1–4 (IEEE, 2021).

32. Zhang Z, Zhang F, Zhang Y, et al. Ultradense one-memristor ternary-content-addressable memory based on ferroelectric diodes. *IEEE Electron Device Lett*. **44**, 64-67 (2022).

33. Lee J W, Kim C, Park G T, et al. Ternary Content-Addressable Memory Using One Capacitor and One Nanoelectromechanical Memory Switch for Data-Intensive Applications. *Adv. Intell. Syst.* **8**, 2500586 (2026).
