# Supplementary material for: Dielectric stacking-engineered scalable reconfigurable transistor platform for adaptive logic circuits
Source: Nat Commun. 2026 Jul 24;17:9028. doi: 10.1038/s41467-026-75864-2 (PMC13507052; doi:10.1038/s41467-026-75864-2)
Supplement: Supplementary file 2 [file 41467_2026_75864_MOESM2_ESM.pdf]

nature portfolio

Peer Review File

Dielectric stacking-engineered Scalable Reconfigurable transistor platform for Adaptive Logic circuits

Corresponding Author: Professor Yen-Fu Lin

This file contains all reviewer reports in order by version, followed by all author rebuttals in order by version.

Version 0:

Reviewer comments:

Reviewer #1

(Remarks to the Author)
Dielectric stacking-engineered Scalable Reconfigurable transistor platform for Adaptive Logic circuits

This work provides a novel approach for scalable reconfigurable transistors and adaptive logic circuits via dielectric stacking engineering. This approach looks interesting. In particular, this approach seems like CMOS-compatible, simple, but the resulting functionality is unique. Although the device performances are well supported by TCAD simulation as well as experimental results, specific information regarding the materials is not extensively provided in the current manuscript. Therefore, I cannot recommend this manuscript to be accepted for publication with this current form. My detailed comments are as follows:
1. This study emphasizes the scalability of the AHA-RFET as a major advantage. However, the current description of the $MoTe_2$ channel fabrication process contradicts this claim. The only relevant details are introduced as mechanical exfoliation. However, how can this approach be highlighted scalability and homogeneity that are required for high-density circuits. Please clarify the synthesis method of the $MoTe_2$ channel material. Even in the Methods section, the authors mentioned WSe_2.
2. The AHA dielectric stack is crucial for nonvolatile polarity reconfiguration, enabling robust charge trapping dynamics. The introduced layer thicknesses are specified as 8/8/24 nm. Please provide justification for the specific thicknesses chosen for each layer of the AHA stack. Specifically, how were the thicknesses of the Al_2O_3 tunneling layer (8 nm) and the HfO_2 trapping layer (8 nm) optimized? Was there a systematic study investigating the trade-off between the tunneling layer thickness (affecting program/erase speed/voltage) and the trapping layer thickness (affecting capacity/retention time)? Demonstrating this optimization is essential to support the high performance reported.
3. What is the role of thin SiO_2 layer on $MoTe_2$? Does it mean the sentence in the Methods “the 3 nm seeding layer deposited before ALD growth of 10 nm HfO_2”? If so, please justify and describe it in detail. Also, what about the $MoTe_2$ degradation? Have the authors seen any oxidation evidence of $MoTe_2$?
4. As shown in Figure S5, why is such hysteresis absent in controlled vdW FETs employing either HfO_2 or SiO_2 dielectric layers?
5. In Figure 3c, the KPFM result should have a scale bar. Also, I recommend recording the KPFM mapping with time to secure operational/ambient stability.
6. In Figure 3b, the hole trapping scheme needs to be modified. Red symbols should go below the line.
7. In Figure 2h, could you describe why this particular temperature (i.e., 358 K) was chosen?

Reviewer #2

(Remarks to the Author)
The demonstration is thorough but the idea is simple and known for more than 8 years now and has been demonstrated at individual to few device level by several other groups. Further, it is unclear to me why trap charge as a mechanism for storing information is appealing with the application is more for compute in memory or using memory elements to do some kind of compute rather than pure data storage. Flash (or trap charge mechanism) is fundamentally slow, energy intensive and not reliable for operation more than several thousand cycles. Why then try to use for compute as opposed to ferroelectric (Nature Communications 2025, 16, 7655; ACS Nano 2024, 18, 4180–4188) or ferromagnetic mechanisms ? Further $MoTe_2$ while an excellent semiconductor, is not very stable under ambient conditions and therefore presents huge challenges for scalable

technology.

Therefore, in its current form I do not recommend publication in Nature Communications.

Reviewer #3

(Remarks to the Author)
I co-reviewed this manuscript with one of the reviewers who provided the listed reports. This is part of the Nature Communications initiative to facilitate training in peer review and to provide appropriate recognition for Early Career Researchers who co-review manuscripts.

Reviewer #4

(Remarks to the Author)
The manuscript presents a scalable reconfigurable field-effect transistor (RFET) platform based on an oxide–van der Waals quasi-floating-gate architecture and top-gate engineering, which enable nonvolatile polarity switching and multi-state programmability. The integration of an $Al_2O_3/HfO_2/Al_2O_3$ (AHA) dielectric stack with a dual-gate design demonstrates reliable non-volatile electrical manipulation, including high endurance (>10^6 cycles), long retention (>10^4 s), and well-balanced N/P operation. The work further showcases solid experimental demonstration on the reconfigurable logic gates and compact ternary content-addressable memory (TCAM), highlighting its potential for logic-in-memory computing. The study is well-structured, experimentally thorough, and supported by TCAD simulations and surface potential analysis. Overall, this manuscript presents an outstanding advancement in reconfigurable logic device design, integrating novel material engineering, robust device physics, and system-level prototyping with a clear path to integration. It addresses multiple key issues that have hampered prior efforts, namely, symmetric polarity switching, retention, endurance, and CMOS compatibility. I support publication in Nature Communications, pending minor revisions to clarify and complete the following points.
1.Regarding the device structure, the manuscript successfully demonstrated the functionality of the AHA stack with a specific thickness. To better establish the design's generality, could the authors comment on whether such a multiple dielectric stacking configuration is a universal design? Are there more material choices for this triple-layer dielectric stack?
2.For physical mechanisms, the authors attributed the nonvolatile polarity switching to charge trapping at oxygen vacancies within the HfO_2 layer, as determined through device structural simulation. Is it possible to characterize this phenomenon experimentally? A more direct investigation of the carrier trapping dynamics would strengthen the discussion.
3.Considering that carrier trapping and tunneling are critical for constructing the non-volatile polarity switching behavior, the material thickness effect of such an AHA stack on the electrical characteristics should be clarified. Does this non-volatile feature strongly rely on a particular thickness? A systematic benchmark in terms of the device parameters, such as dielectric layer, thickness, retention behaviors, and multiple-state features, should be provided.
The manuscript acknowledges the use of mechanically exfoliated $MoTe_2$ as a limitation. Have the authors evaluated the feasibility of CVD-grown or wafer-scale vdW materials for integration? Scalable channels would help contextualize the near-term applicability.

Version 1:

Reviewer comments:

Reviewer #1

(Remarks to the Author)
Based on my careful review on the revised version of the manuscript, it seems like the authors have thoroughly/significantly updated the most part of the reviewers' concerns and comments. However, my biggest concern is still remaining about the scalability. There are several similar conceptual works reported based on $MoTe_2$ with additional functionalities in their individual device level. Although it looks good, largescale synthesis of high-quality $MoTe_2$ is still extremely challenging as the authors show the CVD-grown $MoTe_2$ does not show the similar behavior. Additional issue for $MoTe_2$ is ambient instability. Based on the both critical issues in material synthesis without proper solutions, I am still not sure if this work meets the high standard to be published in Nature Communications.

Reviewer #2

(Remarks to the Author)
The authors have done a thorough job of responding to my comments but I am not convinced that they recognize the central issue of using trap charge mechanism as a mode for computing. I do not think they make the relevant comparison with ferroelectrics where is physics is faster and switching energy is much lower and phenomenon is more robust. None the less, it is a more presentable paper now.

Reviewer #3

(Remarks to the Author)
I co-reviewed this manuscript with one of the reviewers who provided the listed reports. This is part of the Nature Communications initiative to facilitate training in peer review and to provide appropriate recognition for Early Career Researchers who co-review manuscripts.

Reviewer #4

(Remarks to the Author)
I have carefully reviewed the revised manuscript and the accompanying response letter. The authors have clearly made a meticulous and substantial effort to thoroughly address all the concerns raised during the initial review cycle. I particularly commend the newly added experimental data demonstrating the platform's scalable potential, notably the successful extension of the fabrication protocol to large-area $MoTe_2$ arrays and the validation of robust 6-bit multi-level storage in scaled-down (460 nm) devices. Furthermore, the underlying device mechanisms have been convincingly clarified; the inclusion of LFN measurements and time-resolved KPFM decisively validates the interfacial charge-trapping dynamics and the high-quality, low-defect nature of the dielectric stack. Finally, the authors have significantly bolstered the practical value of this work by rigorously evaluating its TCAM application prospects, proving a highly competitive microsecond-range search speed alongside a remarkable 87.5% reduction in hardware footprint compared to traditional SRAM-based cells. These comprehensive revisions have immensely enhanced the technical rigor, clarity, and overall impact of the study. Therefore, I fully support the authors' commendable efforts in elevating this manuscript and recommend it for acceptance and publication.

Open Access This Peer Review File is licensed under a Creative Commons Attribution 4.0 International License, which permits use, sharing, adaptation, distribution and reproduction in any medium or format, as long as you give appropriate credit to the original author(s) and the source, provide a link to the Creative Commons license, and indicate if changes were made.
In cases where reviewers are anonymous, credit should be given to 'Anonymous Referee' and the source.
The images or other third party material in this Peer Review File are included in the article's Creative Commons license, unless indicated otherwise in a credit line to the material. If material is not included in the article's Creative Commons license and your intended use is not permitted by statutory regulation or exceeds the permitted use, you will need to obtain permission directly from the copyright holder.
To view a copy of this license, visit https://creativecommons.org/licenses/by/4.0/

Response to Reviewer #1:

Reviewer #1 (Remarks to the Author):

Dielectric stacking-engineered Scalable Reconfigurable transistor platform for Adaptive Logic circuits

This work provides a novel approach for scalable reconfigurable transistors and adaptive logic circuits via dielectric stacking engineering. This approach looks interesting. In particular, this approach seems like CMOS-compatible, simple, but the resulting functionality is unique. Although the device performances are well supported by TCAD simulation as well as experimental results, specific information regarding the materials is not extensively provided in the current manuscript. Therefore, I cannot recommend this manuscript to be accepted for publication with this current form. My detailed comments are as follows:

We express our sincere gratitude to Reviewer #1 for the meticulous evaluation and constructive feedback, which have been instrumental in enhancing the technical depth and clarity of our manuscript. In response to the reviewer's insightful comments, we have performed extensive additional experiments and provided more rigorous characterizations to solidify our claims regarding the scalability, reliability, and physics of the AHA-RFET, including: **(1) Verification of the fabrication scalability and device uniformity**, where we successfully extended our protocol to large-area synthesized $MoTe_2$ arrays, demonstrating excellent device-to-device uniformity and establishing a robust integration path for wafer-scale van der Waals materials (**Figure R1**); **(2) Systematic optimization of the AHA dielectric stack** (**Figure R3**), where we fabricated and characterized AHA-RFETs with various thickness to verify the specific roles of the tunneling and blocking layers in determining memory performance. This comprehensive study validates that the 8/8/24 nm configuration balances the trade-off between programming/erase speed and non-volatile charge retention effectively; **(3) Clarification of the role of SiO_2 seeding layer**, justifying its necessity for the uniform ALD growth of HfO_2 and its impact on the high-quality interface (**Figure R4**); **(4) Assessment of long-term environmental robustness**, highlighted by an 8-month aging test that confirms near-zero performance degradation and the effective prevention of $MoTe_2$ oxidation through HfO_2 encapsulation (**Figure R5**); and **(5) Enhanced spatial-temporal characterization**, incorporating time-lapse KPFM mapping with precise scale bars to visually confirm the stability of the surface potential and charge-trapping dynamics(**Figure R7**).

All revisions have been carefully incorporated into the manuscript, with detailed point-by-point responses to Reviewer #1's comments provided in the revised version. These efforts, together with the corresponding modifications clearly highlighted in the text, directly address the concerns raised and significantly strengthen the evidence for our proposed AHA-RFET.

1. This study emphasizes the scalability of the AHA-RFET as a major advantage. However, the current description of the $MoTe_2$ channel fabrication process contradicts

this claim. The only relevant details are introduced as mechanical exfoliation. However, how can this approach be highlighted scalability and homogeneity that are required for high density circuits. Please clarify the synthesis method of the $MoTe_2$ channel material. Even in the Methods section, the authors mentioned WSe_2.

We sincerely thank the reviewer for pointing out these critical issues regarding the scalability of the $MoTe_2$ devices and the synthesis method of the channel materials in our manuscript. We have addressed these concerns through additional experimental data and a thorough revision of the experimental methods.

(1) Verification of Scalable Integration Compatibility and Rationale for Channel Selection

We completely agree with the reviewer that mechanical exfoliation is not a scalable approach for high-density circuits. To address this concern, we have verified the fabrication scalability and device uniformity of our AHA FET devices by successfully extending the protocol to large-area synthesized $MoTe_2$ (provided by Six Carbon Tech) field-effect transistor (FET) arrays on AHA substrates (see **Figure R1**). Although the ambipolar conduction cannot be observed, this p-type arrays demonstrate excellent device-to-device uniformity in terms of fabrication yield and threshold voltage distribution, thereby confirming a reliable and scalable integration path for wafer-scale AHA-based van der Waals (vdW) devices.

On the other hand, it should be clarified that our initial claim of "scalability" was primarily based on the process compatibility of the AHA dielectric stack and the device architecture with silicon processing, rather than the synthesis of the 2D channel itself. This is because, currently, the synthesis of high-quality, large-area $MoTe_2$ remains a significant challenge in the field. While our fabricated AHA-FET arrays in Figure R1 exhibit high structural uniformity, the synthesized material currently fails to exhibit the ideal ambipolar performance required for high-performance AHA-RFET fabrication. Therefore, in this work, to demonstrate the proposed AHA-RFET design concept with clarified device mechanisms and tunable N/P-conduction characteristics, high-quality exfoliated ambipolar $MoTe_2$ flakes were used for the main-text investigation, which compromises the device scalability. Importantly, as verified by the uniform FET arrays on $MoTe_2$ films in **Figure R1**, the fabrication protocol itself is fully decoupled from the channel-exfoliation step. Thus, we believe that as large-area synthesis of ambipolar $MoTe_2$ progresses in the community, the same process flow can be directly transferred to wafer-scale integration without architectural modification.

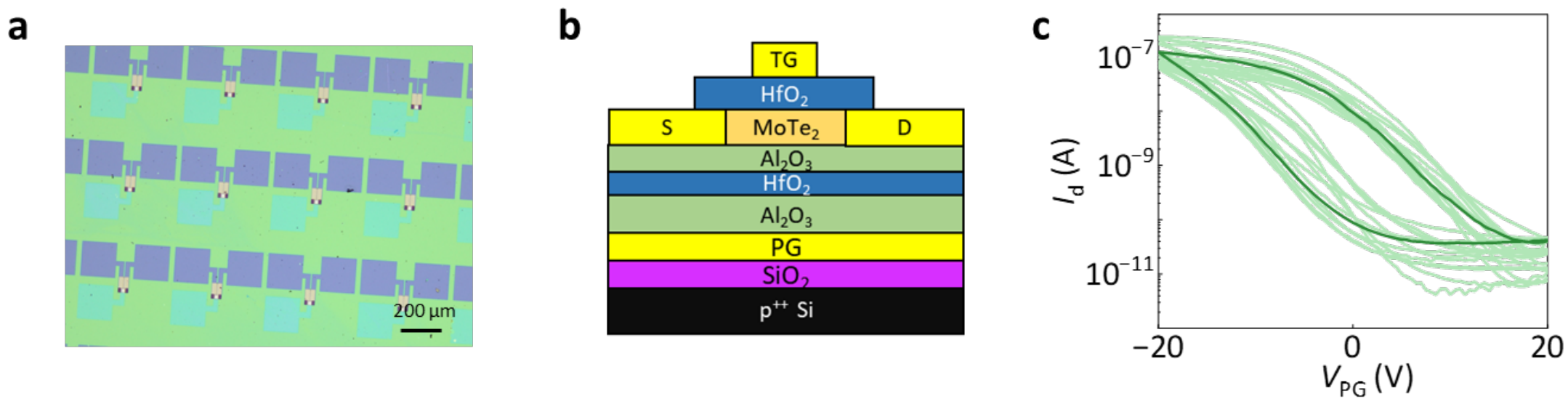

Figure R1. Evaluation of integration potential and uniformity using large-area synthesized $MoTe_2$. a. Optical microscope image of an AHA-RFET array fabricated using CVD-grown $MoTe_2$, with a scale bar of 200 um. **b.** Schematic illustration of the device architecture. **c.** Transfer characteristics (I_d–V_{PG}) of multiple devices within the array, demonstrating high fabrication yield and excellent consistency across the large-area synthesized 2D Material.

(2) Clarification on ambipolar channels in Methods

We are grateful to the reviewer for pointing out the inconsistency in our material descriptions, which has allowed us to clarify our experimental strategy. Actually, we would like to emphasize that the structural design of AHA-RFET is inherently universal and compatible with various 2D semiconductors beyond $MoTe_2$. Throughout our study, we extensively characterized several materials, including WSe_2-based AHA-FETs, to verify this versatility (**Figure R2**). As expected, the AHA stack successfully facilitates reconfigurable behavior in WSe_2 channels. On the other hand, we observed that $MoTe_2$ provides a more balanced performance profile than WSe_2. Such the superior current matching behavior between the P- and N-branches is essential for the logic gate demonstrations presented in the main text. Consequently, the $MoTe_2$-based AHA-RFET results are employed in the main text while the comprehensive data for WSe_2 have been allocated to the Supplementary Information file to support our conclusion of the universal role of the AHA stack.

Regarding the type of WSe_2 in the original Methods section, we have corrected this clerical oversight to $MoTe_2$ to ensure strict consistency with our primary experimental demonstrations. We also added WSe_2-based results in the Supporting Information, and we sincerely apologize for the misunderstanding of the technical route caused by these clerical errors.

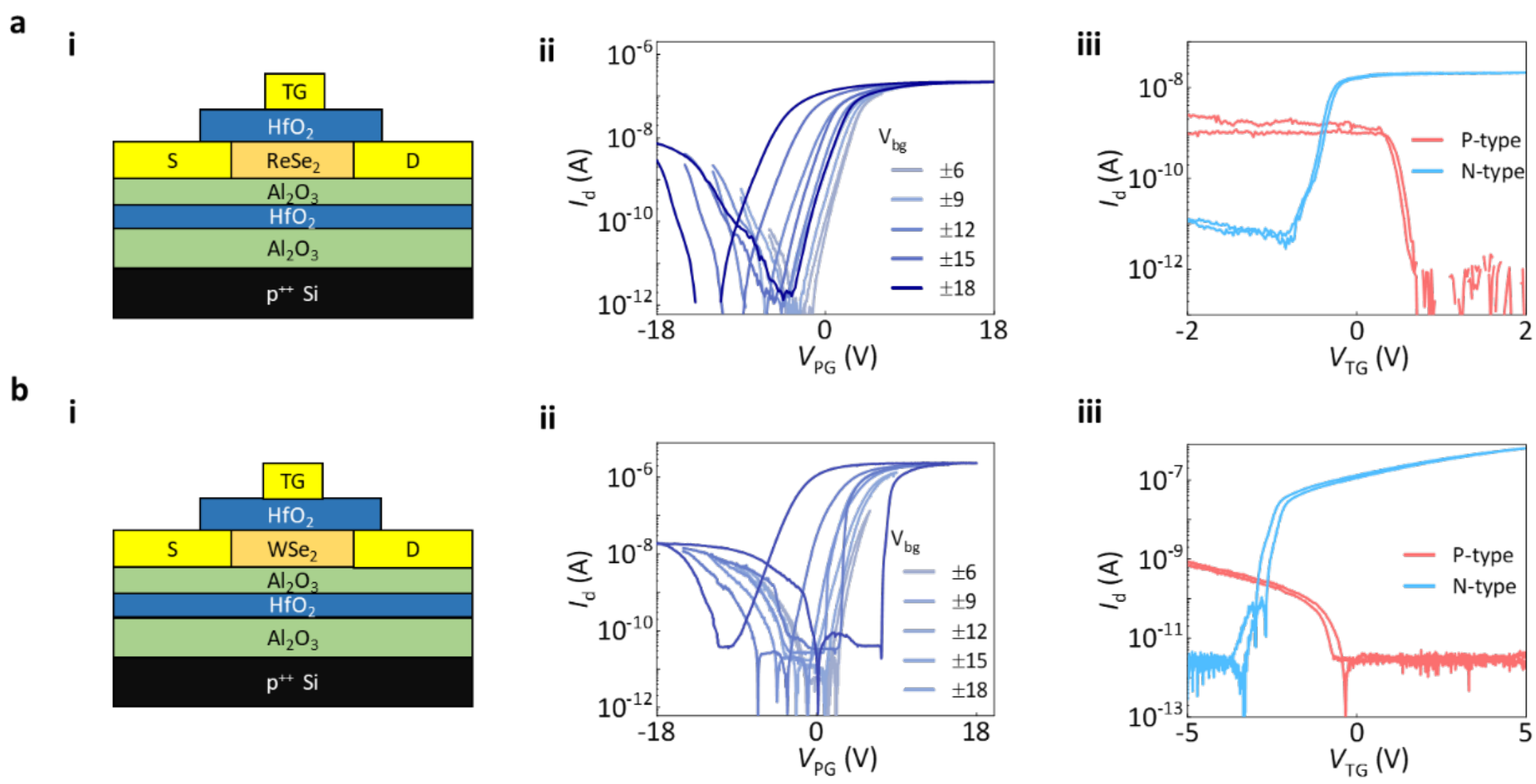

Figure R2. Universal reconfigurability of the AHA-RFET across different 2D semiconductor channels. a, **b** Represents the characterization of AHA-FETs using $ReSe_2$ (**a**) and WSe_2 (**b**) as channel materials, respectively. **i.** Device structural schematics illustrating the top-gated architecture where the AHA dielectric stack is integrated with different 2D semiconductors to verify versatility. **ii**. Gate-tunable transfer characteristics (I_d–V_{PG}) measured at various back-gate voltages (V_{PG}) ranging from 6 V to 18 V, demonstrating the effective modulation of the memory window and carrier density across different materials. **iii**. Ambipolar transport demonstrations showing well-defined P-type and N-type branches for both $ReSe_2$ and WSe_2, verifying that the AHA quasi-floating-gate architecture is inherently universal and compatible with various 2D semiconductors beyond $MoTe_2$.

Corresponding changes made in the manuscript:

In response to the reviewer's insightful comments, we have carefully re-evaluated the manuscript content and the Supplementary Information. Specifically, we have refined the discussion regarding device scalability, clarifying that it primarily refers to the AHA dielectric stack and top-gate integration process rather than the mechanically exfoliated $MoTe_2$ channel. We have also corrected the clerical error regarding the channel material in the Methods section. The corresponding experimental evaluations of large-area synthesized 2D arrays and the universal reconfigurability of the AHA-RFET across different semiconductors, along with relevant discussions, have been added to the revised manuscript as **Figure S13** and **Figure S24**. We hope these changes clarify our technical route and improve the overall accuracy of the work, and we thank the reviewer again for the valuable recommendation.

"Notably, this synergistic polarity modulation extends beyond $MoTe_2$ to WSe_2 and $ReSe_2$, establishing the AHA architecture as a universal strategy for reconfigurable devices (**Figure S13**). Given the better current matching behavior of P- and N-branches,

$MoTe_2$ devices are employed for the following logic-circuit demonstrations." on Page 6.

"It is worth mentioning that, although the precise carrier modulation and synthetic approaches of ambipolar materials currently require further optimization, the AHA-stack structure establishes a robust and scalable manufacturing route for reconfigurable electronic devices (**Figure S24**). Thus, high-quality exfoliated $MoTe_2$ was utilized to achieve optimal current matching and rigorously evaluate the intrinsic physics of the AHA-RFET device." on Page 9.

"The AHA-RFET device is fabricated on a pre-cleaned Si/SiO_2 substrate. First, the bottom gate region is patterned by electron beam lithography (EBL), followed by the deposition of 5/30 nm Ti/Au metal electrodes via electron beam evaporation. After lift-off to remove the photoresist and excess metal, the sample is annealed at 200°C for 20 min in N_2 ambient to eliminate residual photoresist. Next, a high-κ dielectric stack consisting of $Al_2O_3/HfO_2/Al_2O_3$ (8/8/24 nm, from top to bottom) is deposited via ALD at temperatures below 200°C. The $MoTe_2$ active layer is prepared by mechanical exfoliation and transferred onto the substrate using a polydimethylsiloxane (PDMS)-assisted dry transfer method. Subsequently, source/drain (S/D) electrodes are defined by EBL, and 30 nm Au is deposited via electron beam evaporation. After lift-off, the device undergoes another 200°C, 20 min N_2 annealing to improve metal-semiconductor contact. To enhance gate insulation, a 3 nm seeding layer is deposited before ALD growth of 10 nm HfO_2. Finally, interconnect gate electrodes are patterned by EBL, and 30 nm Au is deposited." on Page 13.

2. The AHA dielectric stack is crucial for nonvolatile polarity reconfiguration, enabling robust charge trapping dynamics. The introduced layer thicknesses are specified as 8/8/24 nm. Please provide justification for the specific thicknesses chosen for each layer of the AHA stack. Specifically, how were the thicknesses of the Al_2O_3 tunneling layer (8 nm) and the HfO_2 trapping layer (8 nm) optimized? Was there a systematic study investigating the trade-off between the tunneling layer thickness (affecting program/erase speed/voltage) and the trapping layer thickness (affecting capacity/retention time)? Demonstrating this optimization is essential to support the high performance reported.

The authors thank the reviewer for this insightful question regarding the thickness optimization of the AHA dielectric stack. In response, we have conducted a systematic study by fabricating and characterizing several batches of AHA-RFETs with varying layer thicknesses (tunneling layers of 6, 8, and 10 nm; blocking layers of 20, 24, and 30 nm) to evaluate their operating voltage, response speed, and retention capabilities (Figure R3). Our findings are summarized as follows:

(1) **Operating Voltage Dependence on Blocking Layer Thickness**: As shown in **Figure R3a**, we first evaluated the operating voltage based on the programming gate voltage required to open a distinct memory window for devices with different blocking layer thicknesses (20, 24, and 30 nm). The memory window is quantified as the ratio of the threshold voltage shift to the sweeping range, defined by the formula:

$$\text{Memory Window (\%)}=\frac{\Delta V_{th}}{V_{PG,max}-V_{PG,min}}\times 100\%$$

The variation of the memory window with operating voltage shows that a thinner blocking layer requires a lower voltage to achieve the same proportional memory window. Our systematic characterization confirms that both the required operating voltage and the breakdown threshold increase in correlation with the thickness of the blocking layer. Specifically, for the 20 nm blocking layer, the memory window opens at ±8 V, and the device approaches its breakdown limit at ±12 V. For the 24 nm blocking layer, the window opens at ±12 V, with the breakdown limit near ±15 V. Finally, for the 30 nm blocking layer, the memory window opens at ±15 V, and it nears the breakdown limit at ±21 V.

(2) **Optimization of Response Speed via Tunneling Layer Thickness**: To evaluate the programming speed, we applied a −10 V programming pulse with varying durations (from 10 μs to 1 s) and monitored the transient I_{ds} response. As presented in **Figure R3b**, the variation of drain current response with programming pulse width demonstrates that a thinner tunneling layer significantly accelerates the programming response speed. This is further detailed in the transient I_{ds} responses (**Figure R3d**), which reveal that the 6 nm tunneling layer enables fast switching within 50 μs (**Figure R3d, i**), whereas the 8 nm version requires 100 μs (**Figure R3d, ii**). For the 10 nm version, the response remains limited, requiring significantly longer pulses (up to 500 μs) to achieve a similar current response (**Figure R3d, iii**).

It should be noted that the choice of a 10 V programming voltage is primarily dictated by the hardware constraints of our Keysight B1500A system. Specifically, to capture these microsecond-scale dynamics, we utilized the Fast Measurement Unit (B1530A), which enables ultra-fast pulses down to 50 ns but is strictly restricted to a maximum output of ±10 V. While the High-Resolution Source/Monitor Unit (B1517A) can provide higher voltages, its minimum pulse width is limited to 1 ms, which is insufficient for characterizing the switching behavior in the microsecond regime.

(3) **Retention Capability Enhancement through Tunneling Layer Engineering**: To assess non-volatile reliability, we continuously monitored the on-state and off-state currents over a prolonged period (8000 s) after removing the programming voltage. As shown in **Figure R3c**, the variation of current decay over time indicates that optimizing the tunneling layer thickness effectively suppresses current loss to enhance data retention. The current decay is defined as the ratio of the current change to the initial current, calculated by the formula:

$$\text{Current Decay (\%)}=\frac{I_0-I(t)}{I_0}\times 100\%=\frac{\Delta I}{I_0}\times 100\%$$

The detailed retention tests (**Figure R3e**) demonstrate that for the 6 nm tunneling layer, the high-state current drops noticeably (by nearly an order of magnitude) within 8,000 s (**Figure R3e, i**). In contrast, for both the 8 nm and 10 nm layers (**Figure R3e, ii and**

iii), the high-state current remains nearly constant over the same duration, demonstrating robust non-volatile characteristics.

Based on these systematic comparisons, the 8/8/24 nm configuration was selected as it provides the optimal balance between high-speed operation, a manageable programming voltage, and excellent long-term retention. To support our discussion, we have incorporated these experimental justifications and corresponding data into the revised manuscript as **Figure S3**.

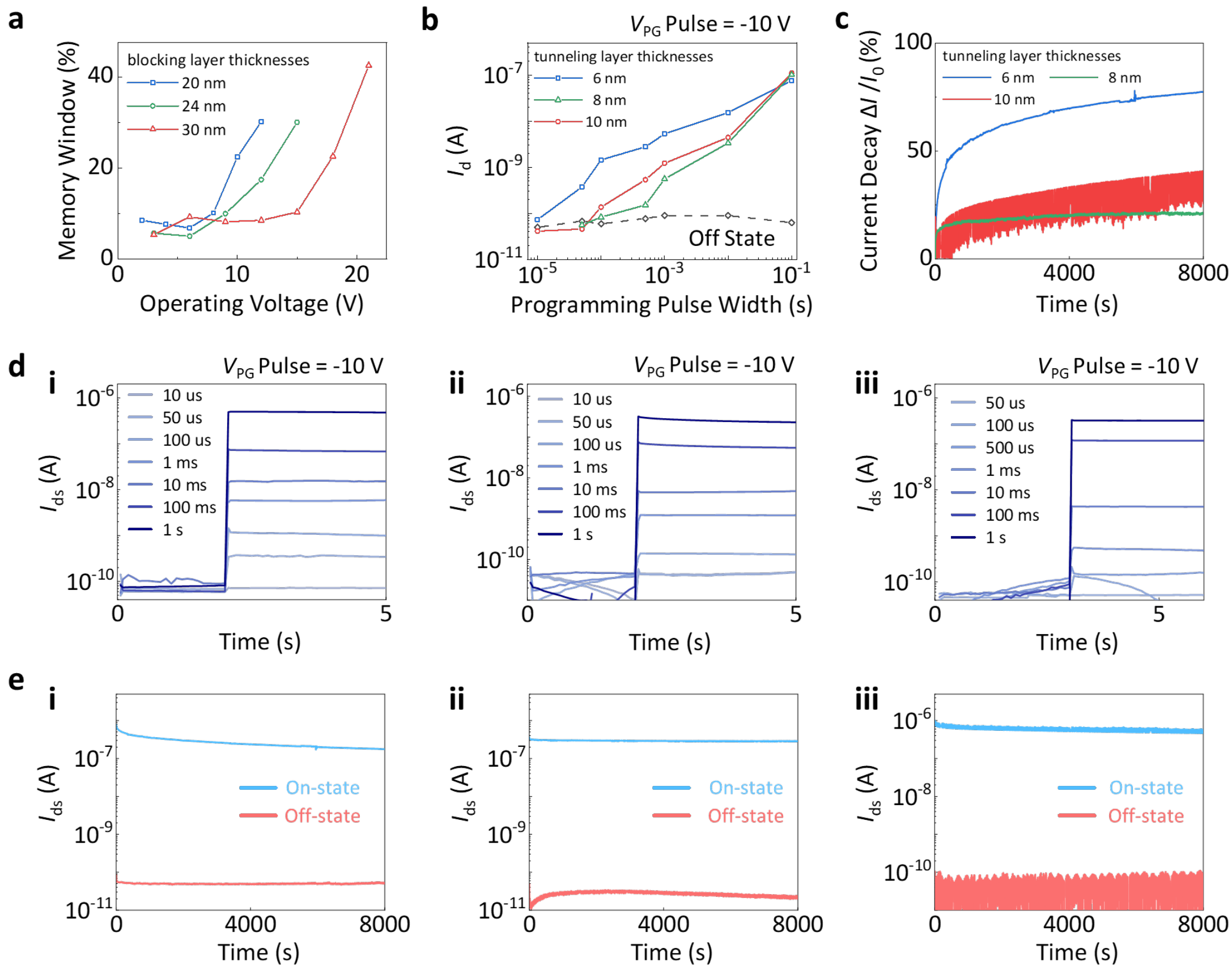

Figure R3. Systematic optimization and justification of the AHA dielectric stack thicknesses. a. The variation of the memory window with operating voltage under different blocking layer thicknesses, showed that a thinner blocking layer requires a lower voltage to achieve a same proportional memory window. **b.** The variation of drain current response with programming pulse width under different tunneling layer thicknesses, demonstrated that a thinner tunneling layer significantly accelerates the programming response speed. **c.** The variation of current decay over time under different tunneling layer thicknesses, indicated that optimizing the tunneling layer thickness effectively suppresses current loss to enhance data retention. **d.** Transient I_d response to pulses of varying widths, reveals that the 6 nm tunneling layer enables fast switching within 50 μs, whereas the 10 nm version requires significantly longer pulses (up to 500 μs) to achieve a similar current response. **e.** Retention tests of the on-state and off-state over 8,000 s, demonstrate the 6 nm tunneling layer leads to noticeable

current decay. **i-iii.** Multi-configuration comparisons across three specific tunneling layer thickness recipes: 6 nm (**i**), 8 nm (**ii**), and 10 nm (**iii**).

Corresponding changes made in the manuscript:

In response to the reviewer's insightful comments, we have carefully re-evaluated the manuscript content and the Supplementary Information. Specifically, we have incorporated a systematic optimization study of the AHA dielectric stack by characterizing various thickness configurations. We have described the comprehensive consideration of the relationship between thickness and various performance metrics to justify our design selection. These experimental justifications and the comprehensive performance benchmarks have been added to the revised manuscript as **Figure S3**. We hope these efforts can clarify our technical route and improve the overall accuracy of the work, and we thank the reviewer again for the valuable recommendation.

"This specific 8/8/24 nm AHA configuration is optimized among various thickness design to precisely balance the trade-off among several critical parameters, such as operating voltage, programming speed, and non-volatile charge retention (**Figure S3**). Such an optimized dielectric engineering ensures both robust reconfiguration and long-term stability for the designed structure." on Page 4.

3. What is the role of thin SiO_2 layer on $MoTe_2$? Does it mean the sentence in the Methods "the 3 nm seeding layer deposited before ALD growth of 10 nm HfO_2"? If so, please justify and describe it in detail. Also, what about the $MoTe_2$ degradation? Have the authors seen any oxidation evidence of $MoTe_2$?

The reviewer's insight is highly professional, and the inference regarding the seeding layer is entirely correct. We apologize for the insufficient description of this component in the original manuscript and have now provided a detailed justification.

(1) Role of the SiO_2 Seeding Layer:

As correctly identified by the reviewer, the 3 nm SiO_2 layer serves as a seeding layer to facilitate the subsequent ALD growth of the 10 nm HfO_2 dielectric. It is known that 2D materials like $MoTe_2$ lack dangling bonds on their surfaces, making direct high-quality deposition of high-k dielectrics extremely difficult. Therefore, the introduction of the SiO_2 seeding layer can improvs surface uniformity and regulate interface charges, traps, and dipoles, to ensure that the subsequently grown HfO_2 film is dense, continuous, and of high dielectric quality (please refer to Adv. Electron. Mater. 7(7): 2000395 (2021).).

To verify this, we performed comparative experiments using devices fabricated without a seeding layer (**Figure R4a**), which resulted in the top-gate dielectric layer that completely failed to exhibit dielectric properties. As shown in **Figure R4b**, the overlapping curves of the drain current (I_d) and gate leakage current (I_g) demonstrate that the top dielectric fails to provide any electrical isolation. This confirms that without the assistance of a seeding layer to facilitate uniform ALD growth, the resulting

dielectric layer lacks the necessary insulating properties and is therefore unfeasible for the fabrication of functional top-gated device architectures.

Furthermore, we discovered that the selection of the seeding layer significantly influences the intrinsic electrical properties of the $MoTe_2$ channel. Specifically, we fabricated and compared double-gate AHA-RFETs utilizing Al_2O_3 and SiO_2 as seeding layers, as shown in **Figure R4c** and **R4d**. We found that while both materials successfully facilitated top-gate dielectric growth and operation, but their impacts on channel doping were markedly different. We surmise that this discrepancy fundamentally arises from the distinct work functions of SiO_2 and Al_2O_3, which induce different degrees of energy band bending at the dielectric-channel interface. Specifically, the integration of the Al_2O_3 seeding layer introduced severe n-type doping to the $MoTe_2$ channel. Consequently, the p-state current dropped by approximately an order of magnitude, leading to the loss of top-gate p-type FET performance (**Figure R4c**). In contrast, the SiO_2 seeding layer exhibited no observable doping effect, effectively preserving the intrinsic ambipolar characteristics of the $MoTe_2$ channel (**Figure R4d**). Thus, in this work, SiO_2 was employed as seeding layer; it not only assists the uniform ALD growth of high-quality HfO_2 but also strictly ensures the balanced bipolarity essential for achieving high-performance.

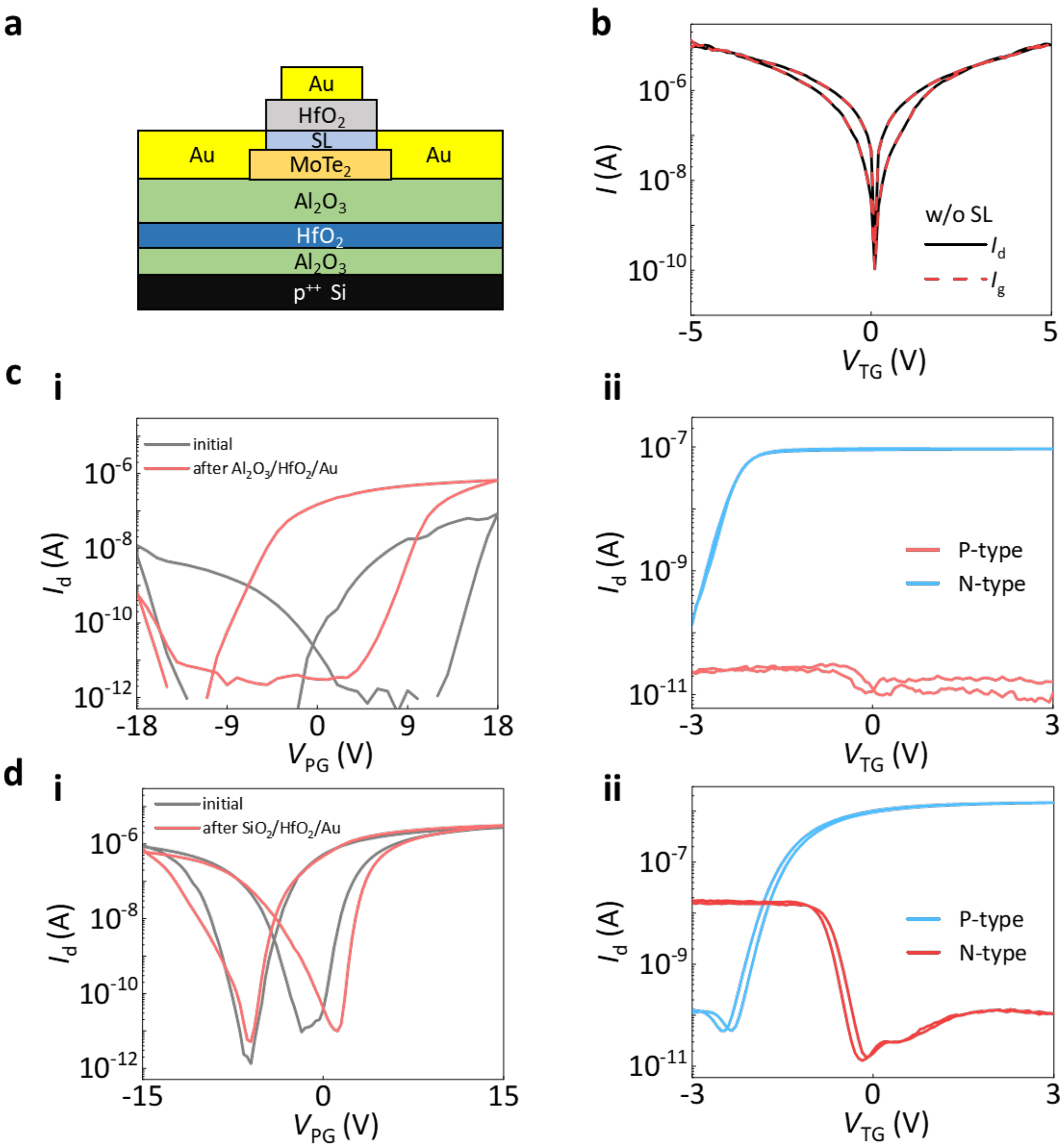

Figure R4. Role of the seeding layer (SL) on top-gate dielectric growth and device performance. a. Device structural schematic illustrating the integration of the seeding layer between the channel and the top dielectric. **b.** Characterization of a top-gate AHA-RFET fabricated without SL. The overlap of I_d and I_g curves ($I_d = I_g$) demonstrates that the top-gate dielectric layer lacks essential dielectric isolation capability. **c.** Performance of a double-gate AHA-RFET with an Al_2O_3 SL. **(i)** Comparison of programming gate transfer characteristics before and after the seeding layer deposition. **(ii)** The device exhibits standard top-gate transfer performance. Both suffers from severe n-type doping. **d.** Performance of a double-gate AHA-RFET with a SiO_2 SL. **(i)** Programming gate (PG) and **(ii)** control gate transfer characteristics show standard reconfigurable transistor performance, confirming that SiO_2 is an ideal SL for assisting HfO_2 growth while preserving the intrinsic bipolarity of the device.

(2) $MoTe_2$ Degradation and Stability:

Regarding the concerns about oxidation, we have re-characterized several devices fabricated over 8 months ago (**Figure R5**). Thanks to the encapsulation provided by the top HfO_2 dielectric, the device performance remained almost identical to the initial measurements, with no evidence of $MoTe_2$ oxidation or degradation. This long-term consistency further proves the stability and reliability of our AHA-RFET.

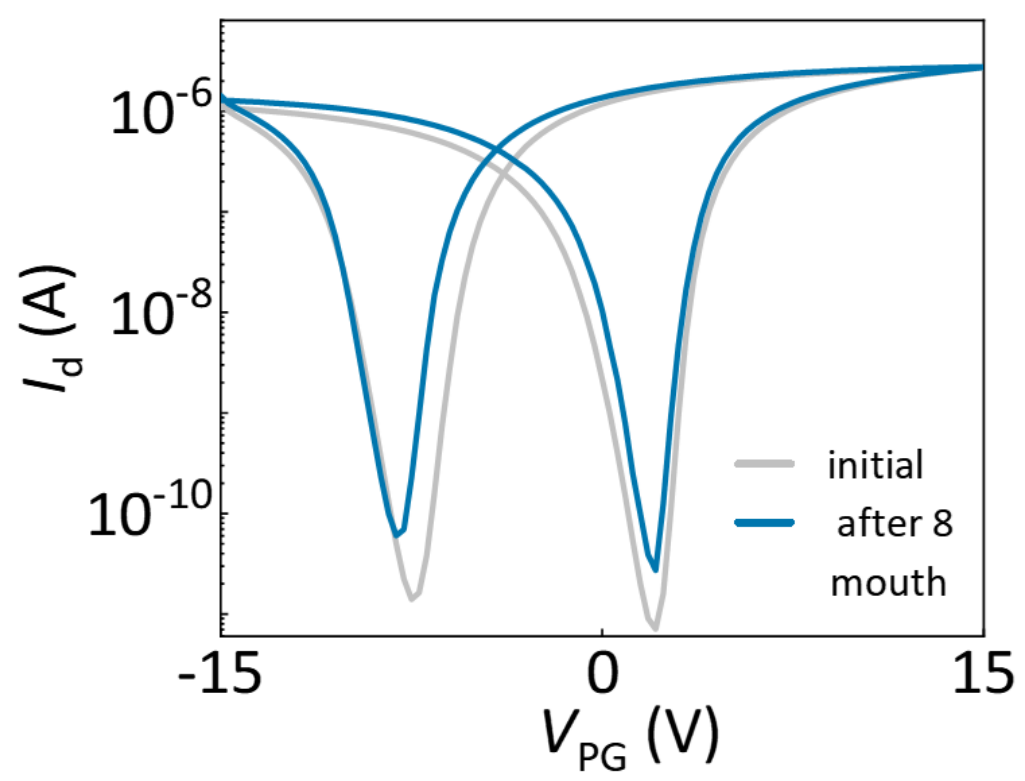

Figure R5. Long-term stability and ambient reliability of the AHA-RFET. Comparison of transfer characteristics (I_d–V_{PG}) for a device measured initially and after 8 months of storage in ambient conditions.

Corresponding changes made in the manuscript:

In response to the reviewer's comments, we have refined the manuscript and Supplementary Information. Specifically, we detailed the necessity of the 3 nm SiO_2 seeding layer for uniform ALD growth on $MoTe_2$ and justified its selection over Al_2O_3 to preserve intrinsic ambipolarity. Furthermore, we have demonstrated that HfO_2 encapsulation effectively prevents $MoTe_2$ oxidation and ensures the devices' long-term environmental stability. These characterizations have been incorporated into the revised manuscript as **Figure S6** and **Figure S23**. We hope these changes clarify our technical route and improve the accuracy of the work, and we thank the reviewer again for the valuable recommendation.

"This hysteresis is highly reproducible across devices with top-gate configuration, during which the HfO_2 layer provides robust encapsulation against moisture and oxygen (**Figure S5**). This scenario is validated by an 8-month ambient aging test, confirming negligible performance degradation and effective protection of $MoTe_2$ (**Figure S6**). Note that related comparison experiments are conducted within a nitrogen-filled glovebox to exclude the effect of ambient artifacts (**Figures S7** and **S8**)" on Page 4.

"Due to the surface dangling bondless feature, a critical component of this fabrication process is the deposition of a 3-nm SiO_2 seeding layer (SL) prior to HfO_2 growth to ensure a dense, continuous dielectric film (**Figure S23**)." on Page 9.

4. As shown in Figure S5, why is such hysteresis absent in controlled vdW FETs employing either HfO_2 or SiO_2 dielectric layers?

We appreciate this insightful question. The absence of hysteresis in our vdW FETs can be fundamentally attributed to the strict isolation from ambient environments during fabrication.

As we know, vdW materials are air sensitive. When exposed to the atmosphere, the bare $MoTe_2$ surface is highly prone to the adsorption of moisture and oxygen, which can further lead to mild surface oxidation. These extrinsic adsorbates and oxidation states act as active charge-trapping centers. During gate voltage sweeps, the dynamic trapping and de-trapping of carriers by these interface states directly cause a noticeable hysteresis in the device's electrical characteristics. Therefore, the hysteresis commonly observed in standard vdW FETs is often an artifact of extrinsic environmental factors rather than the intrinsic properties of the dielectric.

To verify this, we conducted a comparative study between $MoTe_2$ FETs exfoliated and fabricated in ambient air and those prepared within a nitrogen-filled glove box (**Figure R6**). Our results clearly show that the $MoTe_2$ FETs fabricated in air exhibit significant hysteresis, whereas those prepared in the glove box show virtually none. This confirms that the absence of hysteresis in our controlled devices is due to the high-quality, clean interface maintained by our inert fabrication environment, which effectively prevents extrinsic adsorption-induced effects. It is important to emphasize that all exfoliated 2D materials presented in this manuscript were prepared within a nitrogen glove box to ensure optimal device performance and consistency.

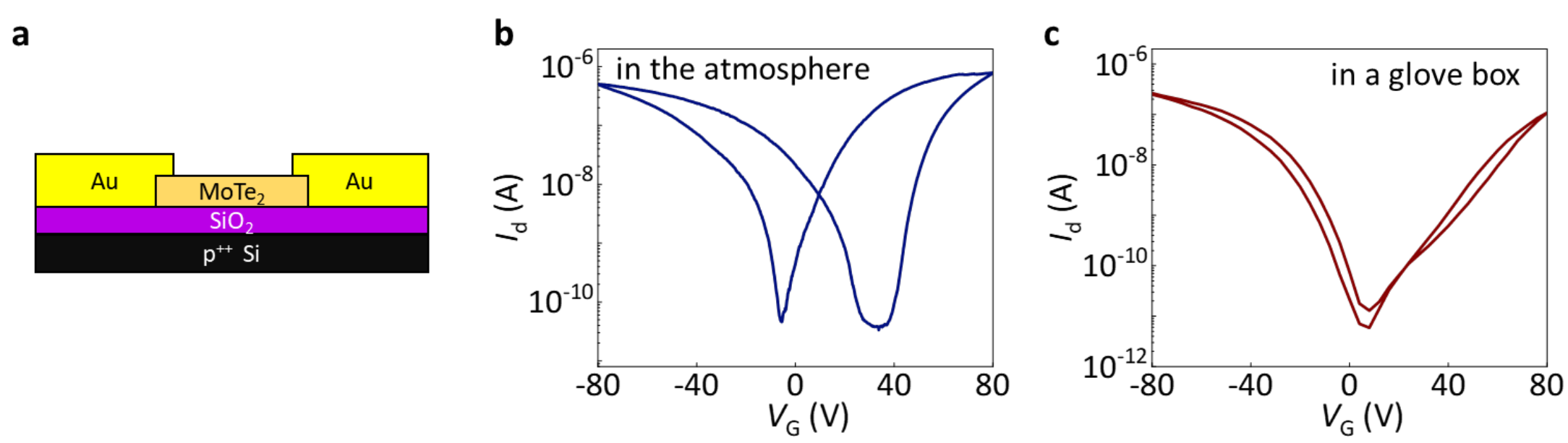

Figure R6. Influence of exfoliation and fabrication environment on the hysteresis behavior of $MoTe_2$ FETs. a. Device structural schematics of $MoTe_2$ FET. **b.** The transfer characteristics (I_d–V_G) of a FET exfoliated and fabricated in ambient air, which exhibits significant hysteresis due to the adsorption of moisture and oxygen on the $MoTe_2$ surface. **c.** The transfer characteristics of a FET where both exfoliation and fabrication were completed within a nitrogen-filled glove box, demonstrating a high-quality and clean interface with virtually no hysteresis.

Corresponding changes made in the manuscript:

In response to the reviewer's inquiry, we have revised the manuscript to emphasize that all 2D materials were fabricated within a nitrogen-filled glovebox to prevent environmental oxidation. Furthermore, we added **Figure S7**, demonstrating that standard dielectrics exhibit no hysteresis under these pristine conditions. These additions definitively confirm that our AHA-RFET's memory window is an intrinsic property rather than an environmental artifact.

"Note that related comparison experiments, which conform memory window originates intrinsically from the AHA stack, are conducted within a nitrogen-filled glovebox to exclude the effect of ambient artifacts (**Figures S7** and **S8**)." on Page 5.

5. In Figure 3c, the KPFM result should have a scale bar. Also, I recommend recording the KPFM mapping with time to secure operational/ambient stability.

We agree with the reviewer that recording the KPFM mapping over time provides a more intuitive understanding of the surface potential dynamics on the dielectric and channel surfaces. Based on Reviewer's suggestions, we have added scale bars to all KPFM images as requested and performed supplementary time-resolved KPFM characterizations to verify the operational and ambient stability (**Figure R7**).

As we can see, in the initial state, the device surface potential is approximately −200 mV. After applying a 10 V reconstruction pulse to switch the device to P-type, the surface potential shifts significantly to −950 mV (0 s) and maintains a value of −700 mV after 10^3 s. Conversely, when a −10 V programming pulse is applied to switch the device to N-type, the surface potential reaches approximately 1.8 V (0 s) and settles at 1.2 V after 10^3 s.

On the other hand, we are grateful for the reviewer's professional reminder regarding the initial omission of the scale bars in the KPFM images. The inclusion of these scale bars provides a necessary spatial reference for interpreting the potential distribution relative to the device dimensions. Accordingly, we have now incorporated the missing scale bars into all relevant KPFM mappings. These time-lapse potential profiles, featuring a 100 nm scale bar, visually confirm the robust charge-trapping/detrapping mechanisms and the excellent operational stability of our AHA-RFET under ambient conditions.

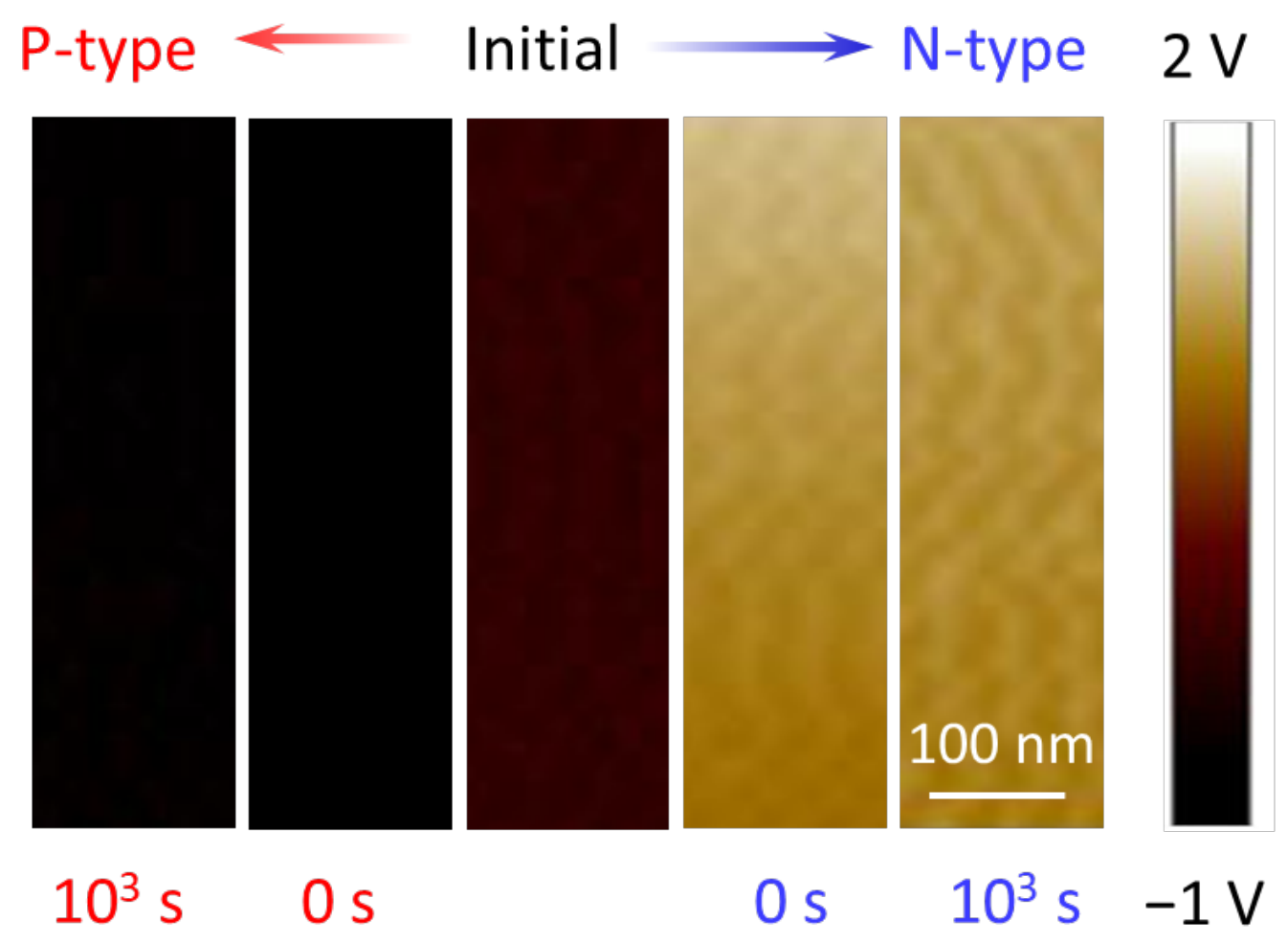

Figure R7. Time-resolved KPFM characterization of surface potential dynamics. Mapping of surface potential evolution over 10^3 s for the device in its initial, P-type (+10 V pulse), and N-type (−10 V pulse) states. The initial surface potential is approximately −200 mV. Following P-type reconstruction, the potential shifts from −950 mV (0 s) to −700 m (10^3 s), while N-type reconstruction shows a shift from 1.8 V (0 s) to 1.2 V (10^3 s). Scale bar is 100 nm.

Corresponding changes made in the manuscript:

In response to the reviewer's insightful comments, we have carefully re-evaluated the manuscript content and the Supplementary Information. Specifically, we have added 100 nm scale bars to all KPFM images to provide a necessary spatial reference for accurately interpreting the surface potential distribution relative to the device dimensions. Furthermore, we have incorporated time-resolved KPFM characterizations to verify the operational and ambient stability of the AHA-RFET, visually confirming the robust charge-trapping and de-trapping dynamics over time. These supplementary characterizations have been added to the revised manuscript as **Figure 3c**. We hope these changes clarify our technical route and improve the overall accuracy of the work, and we thank the reviewer again for the valuable recommendation.

"Besides the energy band analysis, time-resolved KPFM further captures the non-volatile charge storage kinetics within the AHA stack. As shown in Figure 3c, upon applying ±10 V pulses, the surface potential shifts significantly to −950 mV/+1.8 V and remains stable at −700 mV/+1.2 V after 10^3 s, confirming robust retention and operational stability under ambient conditions." on Page 7.

6. In Figure 3b, the hole trapping scheme needs to be modified. Red symbols should go below the line.

We thank the reviewer for the careful review regarding the figure details of the manuscript. As suggested, we have modified **Figure 3b** in the revised manuscript to accurately reflect the physical process of hole trapping. In the updated energy level

diagram (**Figure R8**), the red symbols representing trapped holes have been repositioned below the valence band edge (E_V) within the HfO_2 layer. This correction ensures that the hole trapping scheme is physically consistent with the charge trapping dynamics described in our study.

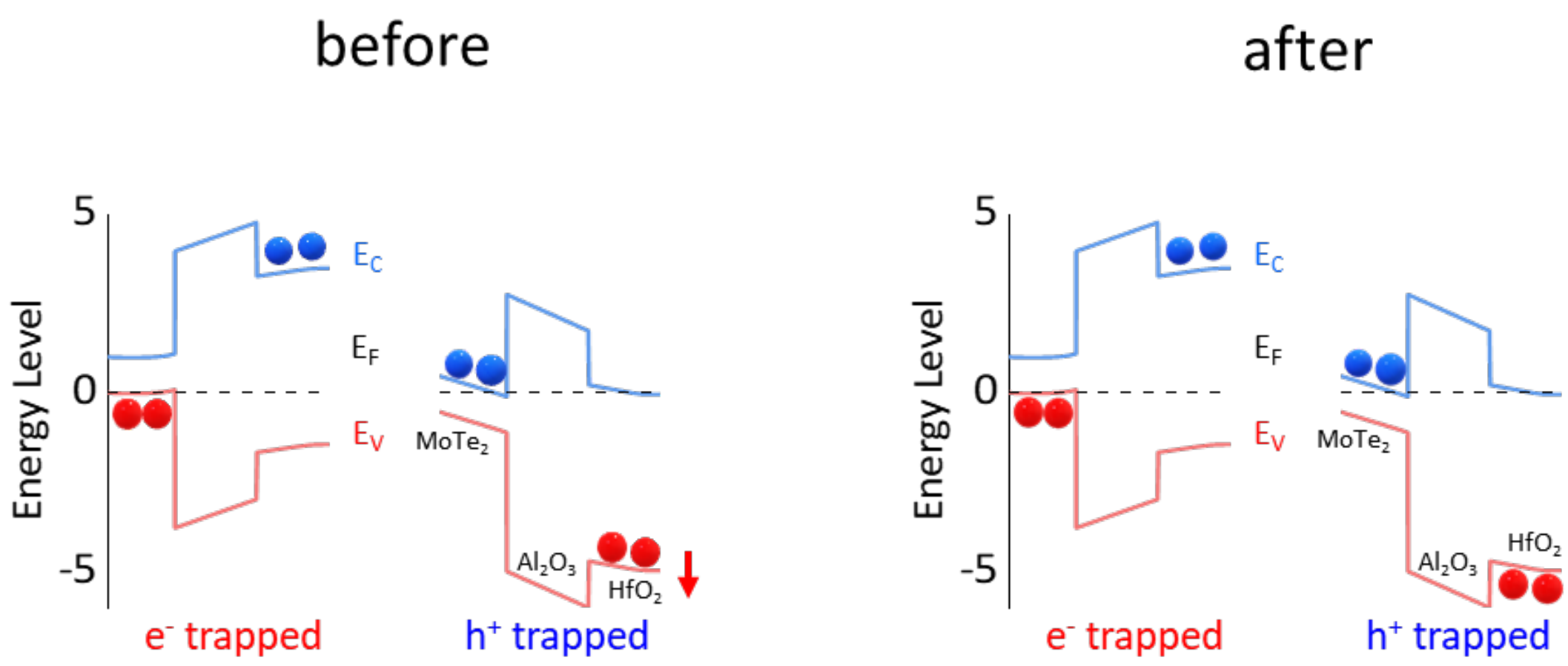

Figure R8. Refinement of the hole trapping mechanism schematic. Energy level diagrams comparing the hole trapping scheme before and after the modification requested by the reviewer. In the "after" schematic, the red symbols representing trapped holes are repositioned below the valence band edge (E_V) within the HfO_2 layer to accurately reflect the physical charge trapping dynamics of the AHA-RFET.

7. In Figure 2h, could you describe why this particular temperature (i.e., 358 K) was chosen?

We appreciate the reviewer's inquiry regarding the selection of 358 K for our thermal stability characterization. This temperature was chosen primarily to ensure the long-term reliability and practical viability of the AHA-RFET.

Since the typical operating temperature range for consumer-grade devices is between 0 °C and 70 °C, achieving a data retention life of more than 10 years at 85 °C (358 K) is considered a key benchmark for a qualified, reliable product. As demonstrated in recent studies on high-density memory and ferroelectric FETs (FeFETs), testing at 85 °C is essential for simulating accelerated aging and verifying that charge-trapping dynamics remain stable under rigorous thermal stress (please refer to Nat. Electron. 1(2): 130-136 (2018); IEEE Journal of the Electron Devices Society, 7: 551-556 (2019); IEEE Electron Device Letters, 42(12): 1786-1789 (2021).). Furthermore, this choice is consistent with common practices in high-impact memory research; studies on various memory architectures, including memristors and thin-film transistors, routinely adopt 85 °C as the standard high-temperature testing point to validate long-term reliability. By demonstrating that our device maintains robust performance at 358 K, we provide strong evidence that the AHA-RFET architecture meets the stringent reliability requirements for future large-scale integrated circuits.

Corresponding changes made in the manuscript:

In response to the reviewer's valuable inquiry regarding our testing conditions, we have updated the manuscript to explicitly clarify the rationale behind selecting 358 K (85 °C) for our thermal stability characterization. We believe this addition provides necessary context for our testing parameters and further strengthens the practical relevance of the device's robust thermal resilience.

"More importantly, this AHA-enabled reconfigurable design demonstrates strong thermal stability, with consistent behavior from room temperature (RT, 300 K) to 358 K for 2000 s (Figure 2i), which is a standard temperature for practical working environments of memory devices." on Page 6.

Response to Reviewer #2:

Reviewer #2 (Remarks to the Author):

The authors report a charge-trap–layer–based $MoTe_2$ non-volatile reconfigurable device and demonstrate its application to logic-in-memory and TCAM circuits. While the manuscript presents interesting experimental data, the overall novelty and technical advancement are not at a level that justifies publication in Nature Communications. Moreover, several claims are overstated or insufficiently supported by fundamental data, and critical device metrics are incomplete or show inherent limitations. For these reasons, I cannot recommend the manuscript for publication. The demonstration is thorough but the idea is simple and known for more than 8 years now and has been demonstrated at individual to few device level by several other groups. Further, it is unclear to me why trap charge as a mechanism for storing information is appealing with the application is more for compute in memory or using memory elements to do some kind of compute rather than pure data storage. Flash (or trap charge mechanism) is fundamentally slow, energy intensive and not reliable for operation more than several thousand cycles. Why then try to use for compute as opposed to ferroelectric (Nature Communications 2025, 16, 7655; ACS Nano 2024, 18, 4180–4188) or ferromagnetic mechanisms? Further $MoTe_2$ while an excellent semiconductor, is not very stable under ambient conditions and therefore presents huge challenges for scalable technology.

We sincerely appreciate Reviewer #2's rigorous evaluation and professional feedback on our manuscript. The reviewer's perspective regarding the inherent speed trade-offs of charge-trap mechanisms compared to ferroelectric or ferromagnetic alternatives is well-taken and has been invaluable in refining the scope of our work. We apologize for any initial lack of clarity regarding the technical metrics and the integration path of our AHA-RFET. Our primary objective is to leverage the unique oxide–vdW quasi-floating-gate architecture, enabling ambipolar 2D materials to achieve balanced, non-volatile reconfigurability. Below, we address the reviewer's concerns by providing substantial experimental evidence to strengthen our demonstrations in the following areas.

(1) Scalability and Material Feasibility

We greatly appreciate the reviewer's attention to the limitations of mechanical exfoliation and the ambient stability of $MoTe_2$. We fully agree that large-area device array fabrication is essential to demonstrating the potential of our AHA-RFET in scalable technologies. In the revised manuscript, we have evaluated the integration feasibility of large-area synthesized (CVD) 2D $MoTe_2$ materials within our fabrication flow (**Figure R10**). The successful demonstration of these FET arrays confirms that our whole integration protocol, including lithography, contact engineering, and top-gate dielectric deposition, is fully compatible with wafer-scale synthesized materials. Furthermore, we have thoroughly revised the manuscript and figure captions to explicitly state that exfoliated $MoTe_2$ flakes were used for fundamental physics

demonstrations, while the CVD-based results serve as a proof-of-concept for industrial scalability.

(2) Dynamic Performance: Pulse-Based Switching Speed, Energy Efficiency, and Endurance

In response to the reviewer's suggestion, we systematically evaluated the dynamic performance of the AHA-RFET through comprehensive pulse-based measurements (**Figure R12 and R15)**. The device achieves fully saturated memory states within a 1 ms pulse—a duration representing the hardware limit of our testing system—while consuming an exceptionally low dynamic energy of approximately 17.1 pJ per operation. Furthermore, cyclic endurance testing under this rapid 1 ms configuration confirms robust reliability, maintaining a stable P/E switching for over 10^6 cycles without significant degradation. These metrics demonstrate that the AHA architecture provides a superior balance between energy efficiency and operational speed, significantly enhancing the technical completeness of our AHA-RFET.

(3) Logic-in-Memory Practicality and TCAM Performance

To address why a charge-trap mechanism is appealing for compute-in-memory, we further characterized the response speed of AHA-RFET-based ternary content-addressable memory (TCAM) circuits (**Figure R13**). While charge-trap devices may not match the raw nanosecond speed of SRAM-based TCAMs, our AHA-RFET offers high density and non-volatile data retention without continuous power consumption. The demonstrated TCAM response justifies its practicality for specific logic-in-memory tasks where balanced N/P operation and multi-state programmability are prioritized over pure switching speed.

(4) Device Micro-scaling and Multi-level Functionality

In response to the reviewer's concern regarding device dimensions and the significance of multi-level states, we have demonstrated the scalability of our AHA-RFET by micro-scaling the device channel length (L_{ch}) to 460 nm (**Figure R16**). Even at this reduced dimension, the AHA-RFET successfully maintains robust multi-level conductance states, confirming that the multi-state programmability is not an artifact of large channel dimensions but a stable feature of the oxide–vdW interface.

We believe these substantial experimental additions and clarifications directly address the reviewer's concerns regarding the scalability, speed, and novelty of our proposed AHA-RFET.

1. The manuscript presents $MoTe_2$-based non-volatile devices, yet the material description is inconsistent. While all main results rely on $MoTe_2$, the Methods section states that WSe_2 was exfoliated and transferred. The authors must clearly state, across the manuscript and figure captions, that exfoliated $MoTe_2$ flakes were used. As written, the manuscript lacks basic technical clarity.

We sincerely thank the reviewer for their meticulous reading and for highlighting this discrepancy in our material description. To address your concerns and improve the technical clarity of the manuscript, we have organized our response into the following two points:

(1) Universality of the AHA Stack Across Multiple Materials

We would like to emphasize that the AHA-RFET is inherently universal and compatible with various 2D semiconductors beyond $MoTe_2$. To specifically validate this broad applicability, our research initially explored a variety of 2D semiconductors, including WSe_2 and $ReSe_2$. As shown in the supplementary data (**Figure R9**), the AHA stack successfully facilitates reconfigurable behavior in both WSe_2 and $ReSe_2$ channels. However, $MoTe_2$ was selected for the main-text investigations because it demonstrated a uniquely balanced ambipolar transport, providing the symmetric P- and N-branch currents essential for the logic circuit demonstrations. Consequently, data for other materials were allocated to the Supplementary Information.

(2) Correction of Clerical Error in the Methods Section

The reference to WSe_2 in the Methods section was an unintended clerical error leftover from our broader material testing phase. We have now corrected this to $MoTe_2$. Furthermore, in strict accordance with the reviewer's advice, we have systematically reviewed the entire manuscript and updated all relevant figure captions to explicitly specify the use of "exfoliated $MoTe_2$ flakes" where applicable. We apologize for the confusion this oversight caused and appreciate the opportunity to improve the technical clarity of our work.

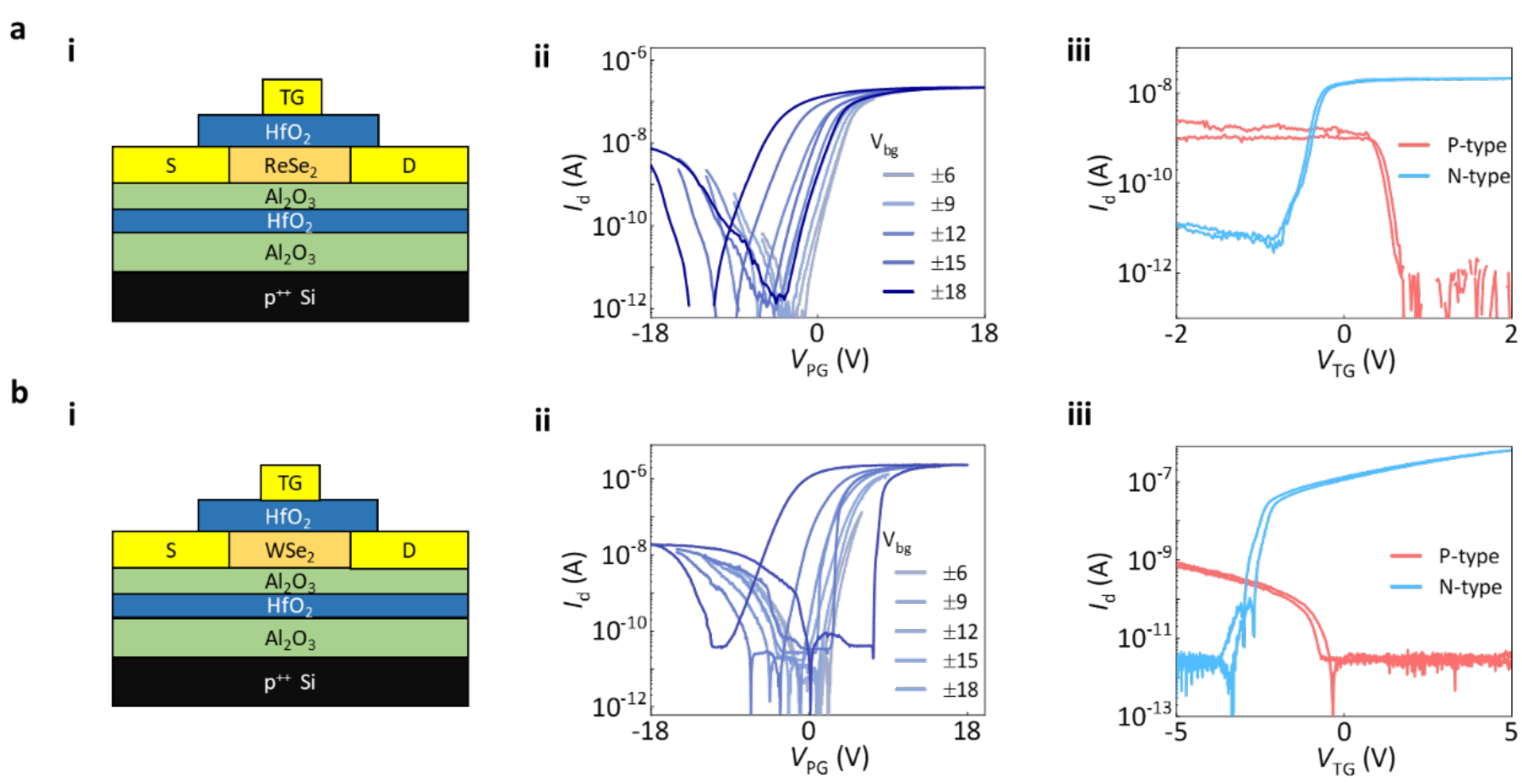

Figure R9. Universal reconfigurability of the AHA stack across different 2D semiconductor channels. The characterization of AHA-FETs using $ReSe_2$ (**a**) and WSe_2 (**b**) as channel materials respectively. **i.** Device structural schematics illustrating

the top-gated architecture where the AHA dielectric stack is integrated with different 2D semiconductors to verify the versatility. **ii.** Gate-tunable transfer characteristics (I_d–V_{PG}) measured at various back-gate voltages (V_{PG}) ranging from 6 V to 18 V, demonstrating the effective modulation of the memory window and carrier density across different materials. **iii.** Polarity Switching under dual-gate control, showing well-defined p-type and n-type branches for both $ReSe_2$ and WSe_2.

Corresponding changes made in the manuscript:

In response to the reviewer's critical feedback, we have thoroughly revised the manuscript to eliminate any ambiguity regarding the channel material. We have corrected the clerical error in the Methods section from 'WSe_2' to '$MoTe_2$' and explicitly specified the use of 'exfoliated $MoTe_2$ flakes' throughout the main text and relevant figure captions. Additionally, to demonstrate the material-agnostic nature of the AHA stack, comprehensive experimental results for WSe_2 and $ReSe_2$ channels have been organized into the Supplementary Information as **Figure S14**. We appreciate the reviewer's attention to detail, which has greatly improved the technical clarity of our work.

"Notably, this synergistic polarity modulation extends beyond $MoTe_2$ to WSe_2 and $ReSe_2$, establishing the AHA architecture as a universal strategy for reconfigurable devices (**Figure S13**). Given the better current matching behavior of P- and N-branches, $MoTe_2$ devices are employed for the following logic-circuit demonstrations. Beyond material versatility, the quasi-floating-gate mechanism is independent of the specific AHA combination. By employing an alternative $SiO_2/HfO_2/SiO_2$ (SHS) stack, we observed similarly consistent non-volatile dynamics and reliable polarity switching (**Figure S14**). These results establish the stack-engineered quasi-floating-gate architecture as a universal and robust strategy for non-volatile polarity modulation in diverse 2D electronic devices." on Page 6.

"The AHA-RFET device is fabricated on a pre-cleaned Si/SiO_2 substrate. First, the bottom gate region is patterned by electron beam lithography (EBL), followed by the deposition of 5/30 nm Ti/Au metal electrodes via electron beam evaporation. After lift-off to remove the photoresist and excess metal, the sample is annealed at 200°C for 20 min in N_2 ambient to eliminate residual photoresist. Next, a high-κ dielectric stack consisting of $Al_2O_3/HfO_2/Al_2O_3$ (8/8/24 nm, from top to bottom) is deposited via ALD at temperatures below 200°C. The $MoTe_2$ active layer is prepared by mechanical exfoliation and transferred onto the substrate using a polydimethylsiloxane (PDMS)-assisted dry transfer method. Subsequently, source/drain (S/D) electrodes are defined by EBL, and 30 nm Au is deposited via electron beam evaporation. After lift-off, the device undergoes another 200°C, 20 min N_2 annealing to improve metal-semiconductor contact. To enhance gate insulation, a 3 nm seeding layer is deposited before ALD growth of 10 nm HfO_2. Finally, interconnect gate electrodes are patterned by EBL, and 30 nm Au is deposited." on Page 13.

2. The repeated claim of "scalability," included in the title, abstract, and main text, is unsupported. All devices are fabricated using mechanically exfoliated $MoTe_2$, which is not scalable by any definition. No wafer-scale growth, large-area integration, yield data, or device uniformity is provided. The scalability claim is misleading and not justified by the presented data.

We thank the reviewer for this critical and rigorous feedback. We sincerely acknowledge that the repeated use of the term "scalability" in our initial manuscript was misleading, as the current channel material is indeed limited by mechanical exfoliation. To address your concerns clearly, we would like to clarify our claims regarding scalability from two distinct perspectives:

(1) Scalability of the AHA Architecture

The authors would like to clarify that, our original intention in emphasizing "scalability" was focused specifically on the AHA quasi-floating-gate architecture and its top-gate integration process. Compared to many reported reconfigurable field-effect transistors (RFETs) that rely on mechanisms inherently difficult to scale, such as exfoliated ferroelectrics (e.g., CIPS please refer to ACS nano, 2025, 19(13): 12948-12959.) or non-standard optoelectronic defects (please refer to Nature Electronics, 2023, 6(10): 755-764.). Our AHA stack utilizes standard ALD, which offers providing a highly scalable structural foundation.

(2) Methodological Scalability Despite Material Limitations

We fully agree with the reviewer that claiming overall device scalability is unjustified when the semiconductor channel depends on exfoliated flakes. However, we wish to emphasize that while we are currently restricted by the material synthesis bottleneck, our top-down fabrication methodology is fully scalable.

To demonstrate this to the reviewer, we have successfully evaluated the integration potential of AHA-RFET's structure by fabricating large-area device arrays using CVD-grown $MoTe_2$ (**Figure R10**). The high structural uniformity of these arrays proves that our entire fabrication protocol, including high-resolution lithography, contact engineering, and top-gate dielectric stacking, is fully compatible with wafer-scale synthesized materials.

While this integration is technically feasible, the primary challenge for applying current CVD $MoTe_2$ specifically to AHA-RFETs lies in its electrical polarity; the $MoTe_2$ we used is currently p-type dominant and lacks the stable, balanced ambipolar characteristics essential for the high-performance reconfigurable logic demonstrated in this work. Consequently, we utilize exfoliated flakes as a reliable medium to verify the fundamental physics of the RFET architecture while material synthesis technology matures toward achieving the necessary balanced bipolarity for large-scale functional logic circuits.

Following the reviewer's professional advice, we have performed a rigorous revision of the abstract and main text to remove misleading claims of overall scalability.

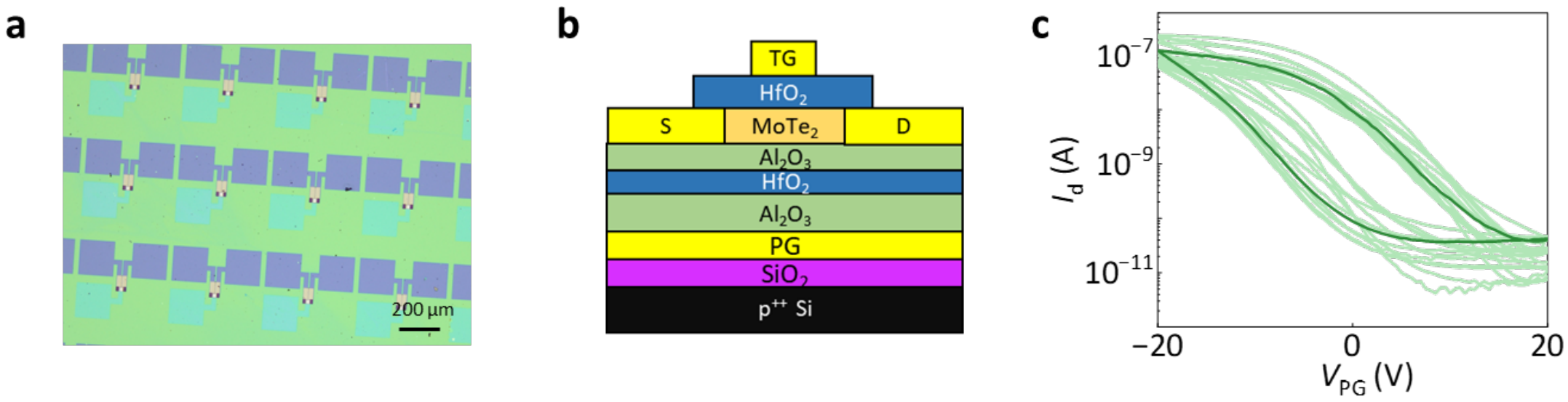

Figure R10. Evaluation of integration potential and uniformity using large-area synthesized $MoTe_2$. a. Optical microscope image of an AHA-RFET array fabricated using CVD-grown $MoTe_2$, with a scale bar of 200 um. **b.** Schematic illustration of the device architecture. **c.** Transfer characteristics (I_d–V_{PG}) of multiple devices within the array, demonstrating high fabrication yield and excellent consistency across the large-area synthesized 2D Material.

Corresponding changes made in the manuscript:

In response to the reviewer's rigorous and constructive feedback, we have carefully re-evaluated the manuscript content. Specifically, we have systematically revised abstract and main text to remove misleading claims regarding overall device scalability, truthfully acknowledging that the core demonstrations rely on mechanical exfoliation. We have further clarified our claims by dividing them into two distinct levels: first, the "scalability" refers strictly to the AHA quasi-floating-gate architecture and our top-down integration methodology; second, while current CVD-grown $MoTe_2$ lacks the balanced ambipolarity required for high-performance logic, our fabrication protocol itself is highly scalable. To empirically demonstrate this methodological scalability, experimental evaluations of large-area device arrays utilizing CVD-grown $MoTe_2$ have been incorporated into the Supplementary Information as Supplementary **Figure S24**. We hope these transparent clarifications and textual revisions significantly improve the technical accuracy of the work, and we thank the reviewer again for the valuable recommendation.

"Here, we demonstrate a reconfigurable field-effect transistor based on the scalable dielectric oxide-van der Waals quasi-floating-gate configuration, enabling nonvolatile polarity switching and multi-state programmability." on Page 1.

"It is worth mentioning that, although the precise carrier modulation and synthetic approaches of ambipolar materials currently require further optimization, the AHA-stack structure establishes a robust and scalable manufacturing route for reconfigurable electronic devices (**Figure S24**). Thus, high-quality exfoliated $MoTe_2$ was utilized to achieve optimal current matching and rigorously evaluate the intrinsic physics of the AHA-RFET device." on Page 9.

3. The choice of $MoTe_2$ further undermines the scalability narrative. $MoTe_2$ is highly unstable in ambient conditions and difficult to synthesize uniformly on a wafer scale. These well-known material limitations directly contradict the authors' suggestion that the device platform has potential for integration. Given this, the technology cannot realistically be considered scalable or manufacturable.

We thank the reviewer for the professional and insightful comments regarding the stability and manufacturability of $MoTe_2$. We sincerely acknowledge that the intrinsic limitations of $MoTe_2$ present real challenges. To directly address these valid concerns, we would like to clarify our technical rationale and experimental validations from two distinct perspectives:

(1) Manufacturability Rooted in Standard Integration Process

Regarding the difficulty of uniform wafer-scale synthesis, we agree that claiming overall "scalability" in the initial text was misleading since the core channel relies on exfoliated flakes. We have revised our claims accordingly. On the other hand, we wish to emphasize that the integration potential we propose is rooted in the AHA quasi-floating-gate architecture itself. Using this standard ALD process and large-area CVD $MoTe_2$, we have successfully fabricated large-scale AHA-FET device arrays that exhibit normal and highly uniform baseline transistor performance (as shown in **Figure R10**). This directly proves that our fabrication methodology is fully capable of large-scale integration. The remaining challenge lies strictly in the material synthesis—specifically, current large-area CVD $MoTe_2$ (provided by Six Carbon Tech) lacks the balanced ambipolarity required for high-performance logic. Thus, we believe that, once the material synthesis technology overcomes this specific bottleneck, our architectural foundation can be readily scaled for large-scale functional circuits.

(2) Resolving Ambient Instability via Architectural Encapsulation

The authors agree with the reviewer that $MoTe_2$ is highly sensitive to ambient conditions. Here, we would like to note that, in our proposed AHA-RFET, the top-gated configuration inherently overcomes this limitation by serving as a robust in-situ encapsulation layer. The high-quality HfO_2 dielectric stack effectively passivates the channel, isolating it thoroughly from ambient moisture and oxygen. To empirically prove this, we have conducted long-term aging tracking of our devices. As shown in **Figure R11**, our AHA-RFET devices based on exfoliated flakes maintain highly stable performance with no significant decay even after 8 months of storage under ambient conditions. This confirms that the environmental stability hurdle has been successfully addressed through our device architecture.

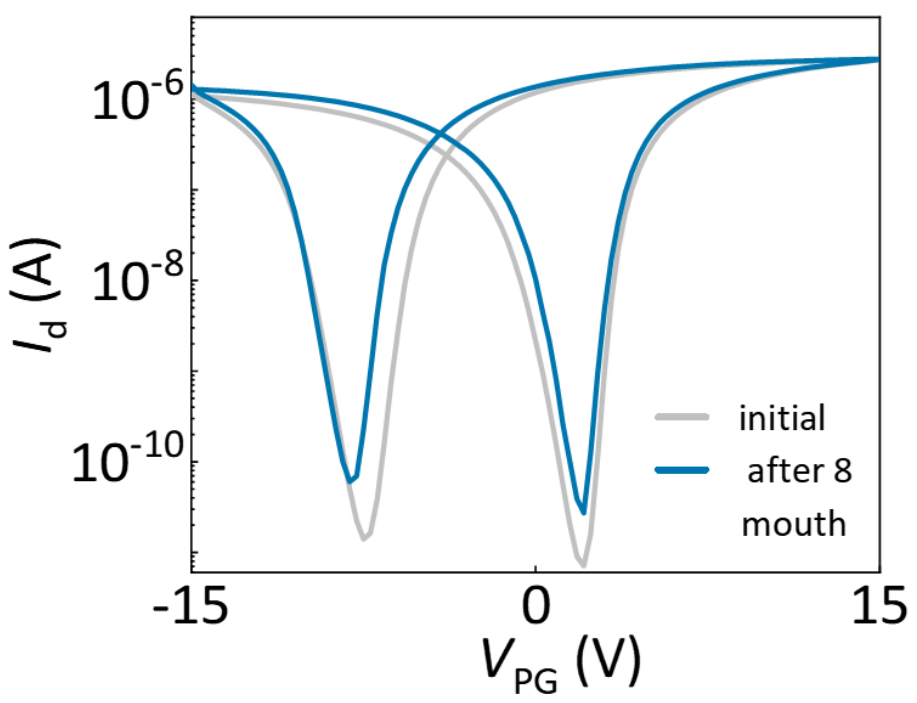

Figure R11. Long-term ambient stability and aging characterization of AHA-RFETs. This plot compares the transfer characteristics (I_d–V_{PG}) of a representative device measured initially and after eight months of storage in ambient conditions. The nearly overlapping performance curves verify that the top-gate HfO_2 dielectric stack serves as an effective in-situ encapsulation layer, protecting the $MoTe_2$ channel from environmental degradation and ensuring robust, non-volatile reconfigurable operation over extended durations.

We appreciate the reviewer's insightful comments regarding the stability and scalability of $MoTe_2$. We fully acknowledge that while the intrinsic air sensitivity of $MoTe_2$ remains a challenge at the bare-material level, our work demonstrates that the AHA-RFET architecture provides a practical solution through its top-gate HfO_2 stack, which serves as a robust in-situ encapsulation layer. This design effectively passivates the channel and shifts device-level stability into a practically usable regime. While the current work focuses on the physical demonstration of this logic architecture using high-quality exfoliated flakes, we are mindful of the requirements for large-area implementation. The synthesis of large-area $MoTe_2$ is an ongoing effort primarily driven by our material-expert collaborators. Moving forward, we will continue to work closely with them to integrate our architectural stability—demonstrated here—with wafer-scale growth and in-situ passivation schemes. This collaborative approach ensures a clear path toward extending our findings to a manufacturable, wafer-scale platform.

Corresponding changes made in the manuscript:

In response to the reviewer's rigorous and constructive feedback, we have carefully re-evaluated the manuscript content. Specifically, we have revised the text to temper our claims regarding overall manufacturability, acknowledging the current limitations of wafer-scale $MoTe_2$ synthesis. Furthermore, to address the concerns regarding material instability, we have added a detailed discussion on how the top-gate HfO_2 dielectric effectively passivates the channel against environmental degradation. This is supported by new long-term aging data, which verifies the ambient stability of our encapsulated AHA-RFETs over an 8-month period. These characterizations have been incorporated into the revised Supplementary Information as **Figure S6** and **S24**. We hope these

systematic revisions significantly improve the technical accuracy of the work, and we thank the reviewer again for the valuable recommendation.

"This hysteresis is highly reproducible across devices with top-gate configuration, during which the HfO_2 layer provides robust encapsulation against moisture and oxygen (**Figure S5**). This scenario is validated by an 8-month ambient aging test, confirming negligible performance degradation and effective protection of $MoTe_2$ (**Figure S6**). Note that related comparison experiments are conducted within a nitrogen-filled glovebox to exclude the effect of ambient artifacts (**Figures S7** and **S8**)." on Page 4.

"It is worth mentioning that, although the precise carrier modulation and synthetic approaches of ambipolar materials currently require further optimization, the AHA-stack structure establishes a robust and scalable manufacturing route for reconfigurable electronic devices (**Figure S24**). Thus, high-quality exfoliated $MoTe_2$ was utilized to achieve optimal current matching and rigorously evaluate the intrinsic physics of the AHA-RFET device." on Page 9.

4. Since the device relies on a charge-trap mechanism, comparison to commercial flash memory is unavoidable. Charge-trap devices operate slowly, often requiring microsecond to-millisecond pulses. The authors do not report any pulse-based programming or erasing speed, leaving the switching characteristics unknown. Commercial flash achieves high density through 3D stacking of hundreds of layers, whereas exfoliated $MoTe_2$ cannot support vertical integration. Claims of potential high-density memory operation are therefore unrealistic.

We thank the reviewer for this critical and practical comment. We apologize for the initial lack of systematic pulse-based characterization in our original manuscript. In response, we have performed switching speed measurements and revised our narrative regarding integration density as follows:

(1) Pulse-Based Switching Speed:

We completely agree with the reviewer that evaluating the programming and erasing (P/E) speed is essential for fully understanding the charge-trap mechanism. To address this, we systematically investigated the P/E dynamics by applying alternating programming (+15 V) and erasing (−15 V) voltage pulses to the program gate. We precisely modulated the pulse widths from 1 ms up to 500 ms and monitored the continuous transient I_{ds} responses. As shown in the **Figure R12**, the transient response curves reveal that the charge-trapping process is highly efficient; the device achieves saturated memory states within just a 1 ms pulse, characterized by a distinct current switching from 10^{-7} A to 10^{-11} A. Extending the pulse duration beyond 1 ms up to 500 ms yields negligible further shifts in the electrical states.

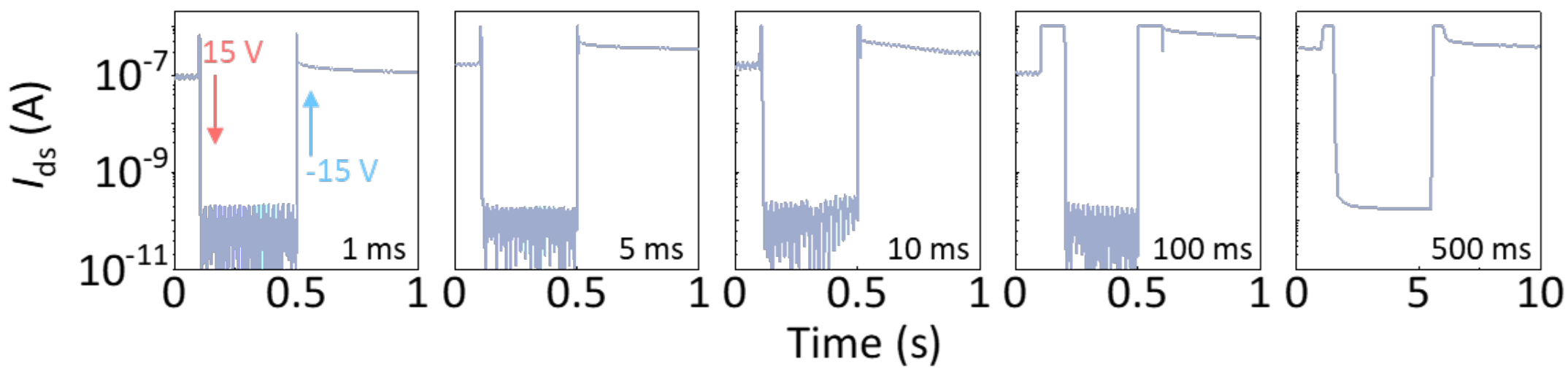

Figure R12. Pulse-based switching dynamics programming/erase speed of AHA-RFETs. Transient drain current (I_{ds}) responses under alternating programming (+15 V) and erasing (-15 V) pulses with varying widths from 1 ms to 500 ms. The device achieves fully saturated memory states within just a 1 ms pulse.

Table R1 The switching speed and operating voltage for various 2D-material-based charge trapping memory devices.

Functional layer	Voltages	speed	References
$Al_2O_3/HfO_2/hBN/WSe_2$	30 V	50 ns	Nat. nanotech. 2023, 18(5): 486-492.
$Al_2O_3/HfO_2/hBN/GR$	4.5 V	20 ns	Nature 2025, 641(8061): 90-97.
$Al_2O_3/HfO_2/hBN/WSe_2$	14 V	1 ms	Nano Lett. 2026. 26, 6, 2089–2097
Al_2O_3/BP	20 V	100 ms	ACS Nano 2016, 10(11): 10428-10435.
$Al_2O_3/HfO_2/Al_2O_3/BP$	18 V	20 ms	Nature Commun. 2022, 13(1): 1485.
$Al_2O_3/HfO_2/Al_2O_3/MoS_2$	26 V	200 ms	ACS Nano, 2015, 9(1): 612-619.
$Al_2O_3/HfO_2/Al_2O_3/BP$	16 V	100 ms	Nanoscale 2016, 8(5): 2686-2692.
$Al_2O_3/HfO_2/Al_2O_3/WSe_2$	40 V	100 ms	Small 2017, 13(17): 1604128.
$Al_2O_3/HfO_2/Al_2O_3/MoS_2$	6 V	1 s	Sci. Bulletin 2019, 64(20): 1518-1524.
$Al_2O_3/HfO_2/Al_2O_3/MoS_2$	18 V	100 ms	Semiconductor Sci. and Tech. 2018, 33(3): 034001.
$Al_2O_3/HfO_2/Al_2O_3/MoTe_2$	15 V	1 ms	This work

It is important to clarify, however, that **this 1 ms switching time represents the hardware limit of our test system (Keysight B2912) rather than the intrinsic physical limit of the device itself**. Specifically, the High-Resolution Source/Monitor Unit required to supply the necessary ±15 V programming voltage has a minimum pulse width limit of 1 ms. Conversely, while our Fast Measurement Unit (B1530A) can generate ultra-fast pulses down to 50 ns, its maximum voltage output is restricted to

±10 V, which is insufficient to fully trigger the charge-trapping dynamics. Therefore, the 1 ms result represents an instrumental lower-bound estimation, and the actual response speed of the device is expected to be faster.

We benchmarked our device against other reported 2D-material-based charge trapping memory technologies (**Table R1**). Devices utilizing ultra-thin two-dimensional h-BN as tunneling layers can achieve nanosecond-level responses; this ultra-fast speed is primarily attributed to the atomically thin nature of 2D h-BN, which facilitates highly efficient direct quantum tunneling across a minimal barrier thickness (Electronic Materials, 2021, 2(4): 495-503.). However, the uniform, large-area synthesis and integration of high-quality h-BN remain technologically immature, posing significant challenges for scalable manufacturing. In contrast, standard oxide tunneling layers(such as Al_2O_3) offer excellent large-area scalability, yet they typically exhibit much slower operation speeds in the tens to hundreds of milliseconds range. Therefore, our 1 ms response time is highly competitive among memories utilizing an $Al_2O_3/HfO_2/Al_2O_3$ functional layer. This demonstrates that our AHA architecture provides a superior balance between large-area scalability and high operational speed.

(2) Integration and High-Density Claims:

On the other hand, we totally agree with the reviewer that comparing exfoliated 2D devices to commercial 3D NAND flash in terms of density is unrealistic. To clarify our original intent and address your concerns, we have refined our claims into the following two aspects:

First, regarding vertical integration compatibility: The primary technical progress in this work is the realization of a fully top-gated architecture using standard ALD process. While we acknowledge that an actual multi-layer 3D demonstration is currently restricted by the large-area synthesis bottleneck of ambipolar 2D materials, this top-down, high-uniformity dielectric stack design inherently establishes the fundamental compatibility of the AHA stack with future 3D vertical stacking processes. It points out a viable technological direction for future integration efforts.

Second, regarding the "high-density" mechanism: We would like to clarify that the "high-density memory" discussed in our initial manuscript referred to the high informational density achieved through multiple conductance states (multi-level storage) via precisely controlled charge trapping within the AHA stack. This mechanism is fundamentally different from commercial flash memory, which achieves high *spatial* density through the physical 3D stacking of hundreds of layers. We realize that our original phrasing lacked this critical distinction and could easily evoke unintended comparisons to commercial 3D NAND.

Therefore, to avoid any misunderstanding and enhance scientific rigor, we have systematically revised the manuscript. We have removed the potentially misleading phrase "high-density memory operation" and replaced it with accurately grounded descriptions such as "multi-level storage capabilities" and "potential for increased logic density."

Corresponding changes made in the manuscript:

In response to the reviewer's critical and practical feedback, we have systematically updated the manuscript to clarify the dynamic performance and integration potential of the AHA-RFET. Specifically, we have performed pulse-based characterizations to determine the switching speed, energy per operation, and endurance, providing a concrete evaluation of the charge-trapping dynamics. Furthermore, to enhance scientific rigor, we have refined our narrative regarding "high-density memory", replacing potentially misleading phrasing with more precise terms such as "multi-level storage capabilities" to distinguish our informational density from the spatial density of commercial 3D NAND. These new results and the corresponding benchmark analysis have been incorporated into the revised **Figure 2c** and Supplementary Information as **Figure S11** and **Table S1**. We believe these revisions address the reviewer's concerns regarding technical clarity and the realism of our claims.

"As shown in Figure 2c, pulses as short as 1 ms effectively trigger conductance switching, with current levels scaling significantly following the varying pulse duration. Consequently, 64 distinct nonvolatile conductance states (6 bit) are realized (Figure 2d). Each state remains stable for 1000 s and is reproducibly modulated across a dynamic range of 10^{-11} A to 10^{-6} A over multiple cycles, highlighting the AHA stack's multi-level storage capabilities (**Figure S9**)." on Page 5.

"Beyond spatial scalability, the AHA-RFET achieves fully P/E switching under rapid pulses with minimal energy consumption, while maintaining robust reversibility and cyclic endurance without significant fatigue behavior of the atomic tunneling layer (**Figure S11**). These metrics are highly competitive among AHA-based charge-trapping devices (**Table S1**)." on Page 5.

5. The TCAM claims are similarly unconvincing. SRAM-based TCAMs operate at nanosecond speeds with high endurance and efficiency, while charge-trap devices are inherently slow and less durable. Given the slow device physics, the proposed device is not practical for TCAM applications, and the claims are overstated.

We thank the reviewer for the rigorous comparison between SRAM-based TCAMs and AHA-based TCAMs. We acknowledge that SRAM-based TCAM excel in nanosecond-level switching; however, we believe these two architectures serve different application scenarios and should not be generalized under a single performance metric. To address your concerns, we have organized our response into the following two points:

(1) Search/Match Speed Based on Transistor Switching

We fully understand the reviewer's concern regarding the operating speed of our device and its potential correlation with the charge-trap mechanism. It is important to clarify that the search and matching speed of the AHA-based TCAM is primarily governed by the dynamic switching speed of the AHA transistor's conduction state (on/off transition) once the logic polarity is fixed. The non-volatile programming speed used for memory

configuration plays a relatively minor role in this high-speed operational phase. Once the device polarity is programmed to define the stored logic, the search operation relies on the rapid electrical response of the gate-to-drain conduction. Consequently, this search process is fundamentally independent of the programming endurance and programming speed associated with the charge-trap structure.

To explicitly demonstrate this, we have detailed our transient response measurements in the supplementary experimental data (**Figure R13**). In this setup, complementary search voltage pulses (±3 V) were applied to the Search Lines (SL), and the transient voltage evolution of the Match Line (ML) was continuously monitored. The results clearly verify that the device successfully executes "Match" and "Mismatch" logic operations, achieving a stable search cycle of approximately 10 μs, which driven by the dynamic switching of the AHA transistor's conduction state. While this search time is currently slower than that of mature high-speed SRAM, we must emphasize that this latency is not an intrinsic limitation. Instead, it is primarily dominated by unintended parasitic RC delays arising from our current experimental circuit layout—specifically, the parasitic capacitance induced by the physical overlap of the match line and search line in vertical space during wiring—rather than the intrinsic switching physics of the charge-trap mechanism.

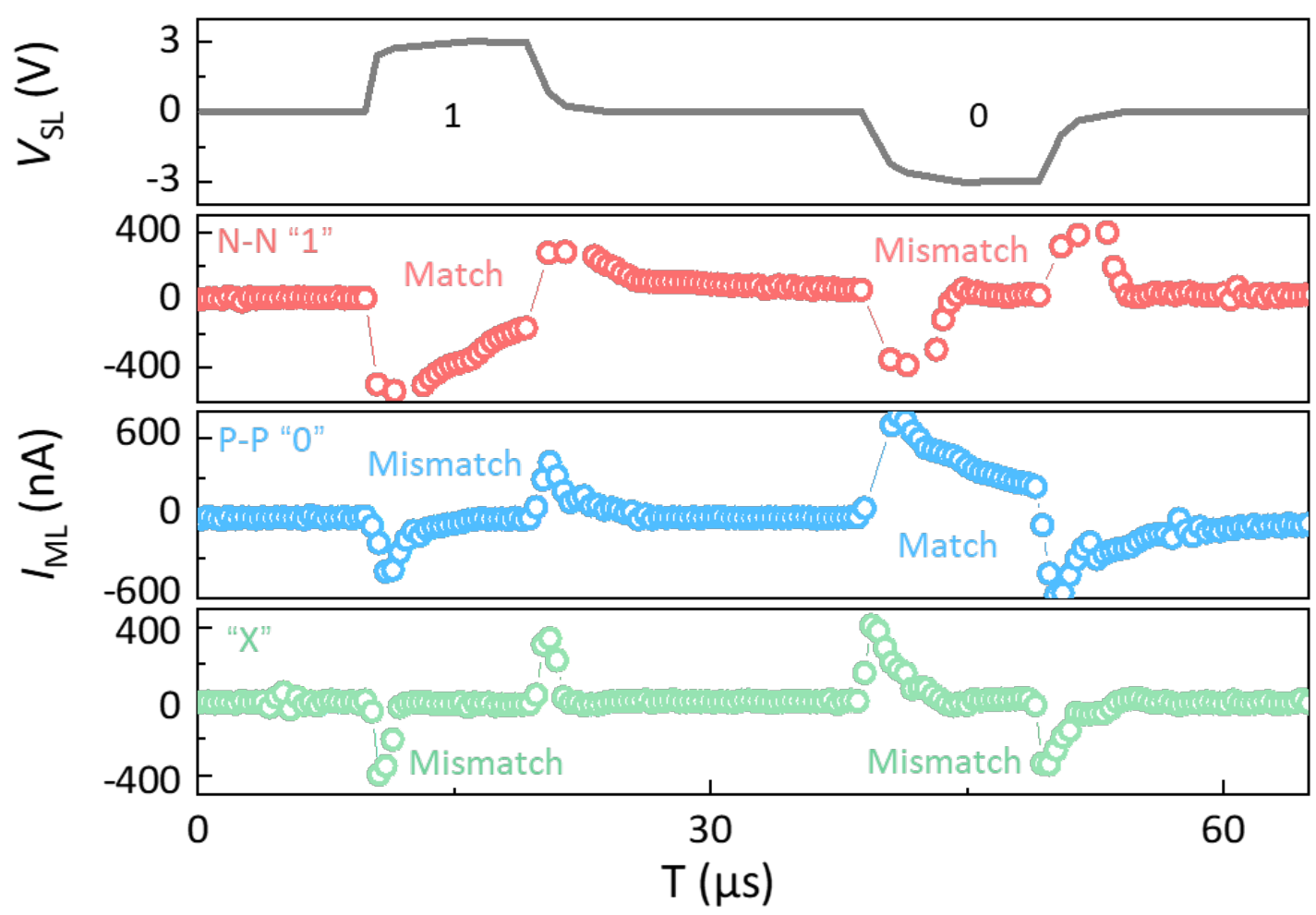

Figure R13. Experimental demonstration of non-volatile TCAM search operations. The timing diagrams illustrate the relationship between the Search Line voltage (V_{SL}) and the resulting Match Line current (I_{ML}) for various logic configurations, including N-N "1", P-P "0", and "X" or "Don't Care". The device successfully executes reliable "Match" and "Mismatch" operations within a microsecond-scale search cycle (approximately 10 μs per search). Search speeds primarily limited by the RC delays of the experimental testing environment rather than the intrinsic charge-trapping physics.

(2) Non-volatility and Area Efficiency Advantages

The reviewer's concerns regarding the performance gap relative to mature SRAM technologies are well-taken and provide a valuable perspective for contextualizing our

work. The authors here would like to clarify that, our AHA-based architecture is primarily designed to deliver systemic advantages through its inherent non-volatility, which serves to eliminate standby power consumption and address the "memory wall" challenge. These features offer a distinct and complementary value proposition compared to traditional volatile SRAM-based TCAMs. To emphasize the advantages of the proposed AHA-RFET TCAM devices, we survey related works and collect them in **Table R2** to support our discussion.

As summarized in **Table R2**, while SRAM-based TCAMs possess an advantage in nanosecond-level switching, our AHA-based TCAM operates on a fundamentally different paradigm. Our search speed is independent of the charge-trap programming speed and is currently limited by extrinsic RC delays rather than intrinsic device physics. It notes that, even though under these constraints, our device achieves a stable search cycle of 10 μs, which is highly competitive and comparable to recent state-of-the-art reports for emerging memory-based TCAMs. Furthermore, our AHA-RFET TCAM offers a massive advantage in area efficiency: traditional SRAM TCAM cells typically require a complex 16-transistor (16T) configuration, whereas our design achieves equivalent functionality using only 2 transistors (2T). This significant reduction in hardware footprint makes it a highly compelling candidate for read-dominant, edge-computing search tasks where hardware density and energy efficiency are prioritized.

Table R2 Performance comparison of search speed and cell composition among various reported CAM/TCAM architectures

Kind	Composition	Speed	References
CAM	2T	500 μs	Nat. Nanotechnol. 2026, 21(2): 198-206.
CAM	2C	10 μs	Nat. Commun. 2025, 16(1): 8047.
TCAM	2T	100 μs	Nat. Electron. 2019, 2(11): 521-529.
TCAM	2C	5 μs	Small 2025, 21(9): 2408618.
TCAM	2T	5 μs	IEEE Electron Device Letters 2025, 46(5): 872-875.
TCAM	2T1R	100 μs	IEEE Transactions on Electron Devices 2022, 69(9): 5262-5269.
TCAM	2T1R	500 ns	2021 IEEE international Electron devices meeting (IEDM). IEEE, 2021: 1-4.
TCAM	1M	100 ms	IEEE Electron Device Letters 2022, 44(1): 64-67.
TCAM	1C1N	17.5 μs	Advanced Intelligent Systems 2026, 8(3): 2500586.
TCAM	2T	10 μs	This Work

Corresponding changes made in the manuscript:

To address the reviewer's concerns, we transient search operation (10 μs) has been added to **Figure 5f** and **Table S2**, with a discussion on how parasitic RC delays, rather

than intrinsic physics, currently limit the speed. The 2T-per-cell area efficiency is also highlighted as a key advantage over traditional 16T SRAM-TCAMs.

"The searching speed of the AHA-RFET NAND TCAM is experimentally characterized (Figure 5f). Although its microsecond-range search speed is currently limited by testing environment RC delays, as summarized in **Table S2**, our device achieves a stable searching operation of 10 μs, which is highly competitive and comparable to recent state-of-the-art reports for TCAMs. Furthermore, the AHA-RFET TCAM provides a highly area-efficient solution, reducing the cell footprint to 2T from the conventional 16T requirement." on Page 12.

6. The charge-trap layer sits beneath the $MoTe_2$, and the presented top-gate architecture does not show compatibility with multilayer or 3D integration. Unless they can be stacked as a top gate, the scalability cannot be proved. Commercial charge-trap technologies already exist in far more advanced forms, making the structural novelty questionable.

We sincerely appreciate the reviewer's rigorous evaluation regarding the structural architecture, its compatibility with 3D integration, and the novelty compared to existing commercial technologies. These are critical aspects, and we welcome the opportunity to clarify our design choices and core innovations.

(1) Feasibility of Top-Gate Architecture and 3D Integration

The authors fully understand the reviewer's concern regarding scalability based on our current prototype, where the charge-trap layer sits beneath the $MoTe_2$. Here, we would like to note that, this configuration is entirely compatible with the top-gate stacking, where the charge-trap layer can be fabricated on top of the $MoTe_2$ channel. This scenario could be experimentally confirmed by previous work on top-gate AHA stack FETs (please refer to ACS Nano, 9(1): 612-619, 2015). In this literature, similar dielectric stacks can be successfully grown on top of 2D materials to form functional top-gate architectures. Thus, in our work, for the initial demonstration, we opted for the bottom-gate configuration primarily to manage experimental costs and simplify the lab-scale fabrication process. Therefore, the fundamental physical mechanisms and material stack are entirely compatible with multilayer and 3D vertical stacking once transitioned to a top-gate configuration in a more advanced fabrication flow.

Furthermore, our present experimental results also can well support this direct 3D-compatibility argument. On one hand, **Figure R14a** demonstrates a functional top-gated stack grown directly on the 2D channel. Recognizing that 2D materials like $MoTe_2$ lack surface dangling bonds—which makes direct high-quality ALD of dielectrics extremely difficult—we successfully introduced a thin SiO_2 seeding layer. This seeding layer facilitates uniform ALD growth, ensuring the top dielectric is dense and continuous, and confirms that the dielectric stack retains its charge-trapping and polarity-reconfiguration behavior when sitting above the $MoTe_2$ layer (**Figure R14b,c**). On the other hand, **Figure R14d** shows that this identical SiO_2-assisted top-gated integration strategy extends seamlessly to large-area synthesized $MoTe_2$ arrays. Taken

together, these two observations establish that the bottom-gate prototype in the main text is a deliberate engineering choice for clean physics extraction, not a structural limitation, and that the same functional stack can be robustly deposited as a top gate over a lower-layer channel, which is exactly the geometry required for scalable multilayer vertical integration.

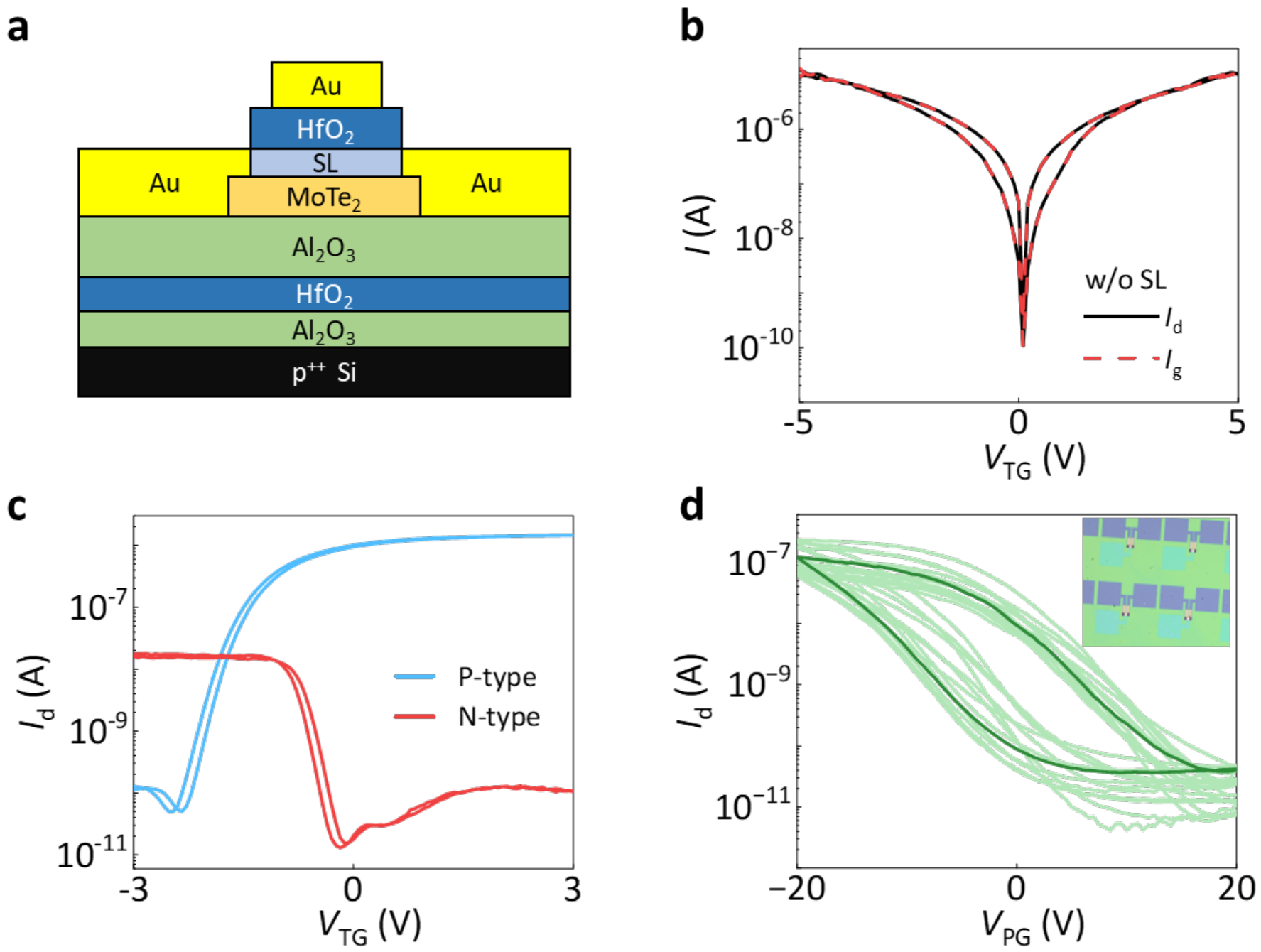

Figure R14. Top-gate exploration of the AHA-RFET. (a) Schematic illustration of the top-gated device structure with a seeding layer (SL). (b) Top-gate transfer characteristics (I_d–V_{TG}) of the device fabricated without a seeding layer (w/o SL). The overlapping drain current (I_d) and gate leakage current (I_g) indicate complete dielectric breakdown. (c) Top-gate transfer characteristics of the device utilizing a thin SiO_2 seeding layer to facilitate top dielectric growth, demonstrating distinct and robust polarity reconfiguration (P-type and N-type). (d) Application of this SiO_2-assisted top-gate integration strategy to CVD-grown $MoTe_2$. The main panel shows the transfer characteristics of the large-area arrays, and the inset displays the optical microscopy image of the fabricated device array.

(2) Core Innovation of AHA-RFET

We appreciate the reviewer's constructive perspective, here, we would like to clarify that the AHA-RFET and commercial charge-trap flash are designed for distinct functional objectives. Commercial charge-trap flash is a mature technology highly optimized for static, high-density bit storage. In contrast, our work focuses on a reconfigurable logic-memory fusion cell intended for dynamic computing tasks.

The primary distinction lies in the physical utility of the charge-trapping effect. While traditional charge-trap flash uses trapped charges to define binary memory states (0 or 1), our AHA-RFET leverages the AHA stack to actively modulate the conduction

polarity of the 2D channel. This capability enables the device to transition between logic, memory, and TCAM functions within the same physical footprint—a versatility not provided by standard flash architectures. Therefore, the core contribution of this work is the functional flexibility and logic-memory co-design.

Corresponding changes made in the manuscript:

In response to the reviewer's critical and professional feedback regarding structural architecture and 3D compatibility, we have revised the manuscript to clarify our integration roadmap and core innovation. Specifically, we have added a detailed discussion and new experimental data demonstrating a functional top-gated Architecture enabled by a SiO_2 seeding layer. This validates the AHA structure's inherent compatibility with 3D vertical stacking and its seamless extension to large-area CVD materials. These revisions, supported by new data in **Figure S23** and **Figure S24**, clarify that our current bottom-gate prototype is a deliberate choice for physics extraction rather than a structural constraint.

"Due to the surface dangling bondless feature, a critical component of this fabrication process is the deposition of a 3-nm SiO_2 seeding layer (SL) prior to HfO_2 growth to ensure a dense, continuous dielectric film (**Figure S23**)." on Page 9.

"It is worth mentioning that, although the precise carrier modulation and synthetic approaches of ambipolar materials currently require further optimization, the AHA-stack structure establishes a robust and scalable manufacturing route for reconfigurable electronic devices (**Figure S24**). Thus, high-quality exfoliated $MoTe_2$ was utilized to achieve optimal current matching and rigorously evaluate the intrinsic physics of the AHA-RFET device." on Page 9.

7. The manuscript also lacks essential pulse-based programming and erasing measurements. For any non-volatile, switching speed, energy per operation, and speed–endurance trade-offs should be reported. Also, the authors should clearly report pulse based programming/erasing and report the highest possible switching speed.

We thank the reviewer for highlighting the necessity of these dynamic performance characterizations. We completely agree that pulse-based switching speed, energy consumption, and endurance are critical metrics for evaluating any non-volatile memory. To address this, we have systematically supplemented the required pulse-based measurements and evaluations. Our detailed responses are as follows:

(1) Pulse-Based Switching Speed:

Firstly, we systematically investigated the P/E dynamics by applying alternating pulses (±15 V) to the gate, modulating widths from 1 ms to 500 ms. As shown in **Figure R15a**, the I_{ds} responses reveal highly efficient charge-trapping, with the device achieving saturated memory states within just 1 ms. This is evidenced by a distinct current switch from 10^{-7} A to 10^{-11} A. Negligible further shifts beyond 1 ms confirm rapid saturation of the trapping sites at this voltage.

It is important to clarify that this 1 ms switching time represents the hardware limit of our test system (Keysight B2912) rather than the intrinsic physical limit of the device itself. Specifically, the High-Resolution Source/Monitor Unit required to supply the 15 V programming voltage has a minimum pulse width limit of 1 ms. Conversely, while our Fast Measurement Unit (B1530A) can generate ultra-fast pulses down to 50 ns, its maximum voltage output is restricted to 10 V, which is insufficient to fully trigger the charge-trapping dynamics. Therefore, 1 ms represents the highest possible switching speed accessible with our current instrumentation, and the actual physical response of the device is expected to be faster.

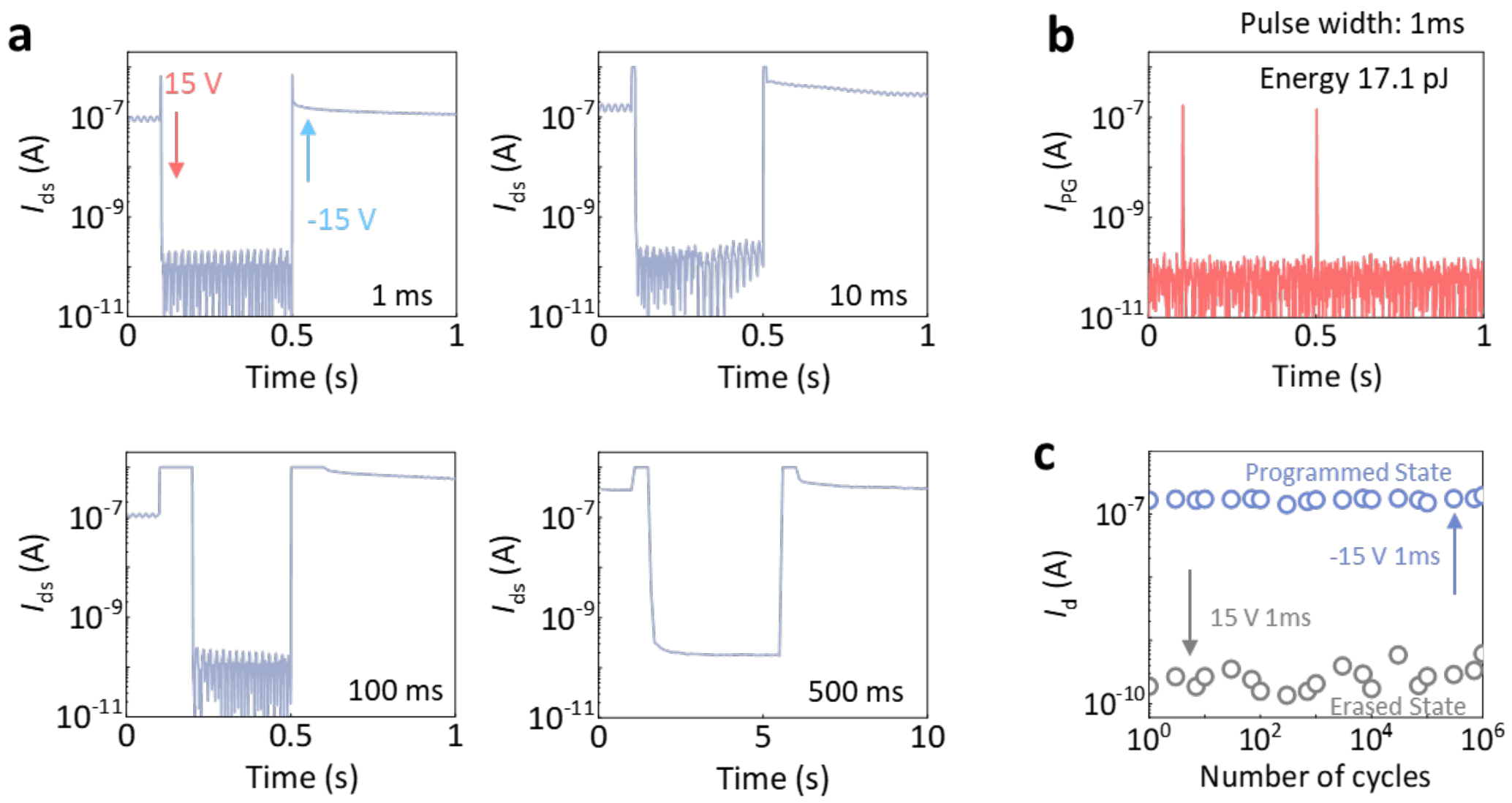

Figure R15. Transient dynamic characterizations and reliability analysis. a. Transient drain current responses measured over time upon the application of alternating programming and erasing voltage, with widths varying from 1 ms to 500 ms. b. Transient programming gate current (I_{PG}) response during a 1 ms, +15 V pulse, with calculated operation energy consumption of 17.1 pJ. c. Cyclic endurance testing at the fastest accessible pulse configuration (1 ms, ±15 V), maintaining a stable memory window over approximately 10^6 cycles before device failure.

(2) Energy per Operation

Based on the pulse-based measurement of the programming gate current (I_{PG}) during dynamic switching, we evaluated the dynamic energy consumption per operation (**Figure R15b**). The dynamic dissipation is estimated using $E = V_{PG} \times I_{PG} \times t$, where V_{PG} represents the amplitude of the programming gate pulse, I_{PG} is the corresponding transient gate current measured during the pulse, and t denotes the pulse duration. For the 1 ms, +15 V pulse, the calculated dynamic energy per operation is approximately 17.1 pJ. Notably, this energy consumption is orders of magnitude lower than that of Magnetoresistive Random-Access Memory (MRAM) (please refer to IEEE Electron Device Letters 36, 402-404 (2015)), which typically operates at the μJ level, and is

highly competitive with Resistive Random-Access Memory (RRAM) (please refer to Nature 464, 873-876 (2010)), which generally ranges from 10 to 100 pJ. This exceptionally low energy consumption, achieved even at the instrument's lower bound speed, highlights the excellent insulation and efficiency of the AHA stack during dynam operation.

(3) Speed–Endurance based on 1 ms pulse

To address the reliability under dynamic conditions, we performed cyclic endurance testing utilizing the fastest accessible pulse configuration (1 ms, ±15 V). As shown in **Figure R15c**, the AHA-RFET maintains a distinct memory state over 10^6 P/E cycles without significant degradation before device failure. While a full continuum study of speed-versus-endurance trade-offs is currently constrained by our instrumentation, these results confirm that operating at the highest accessible speed (1 ms) does not induce severe fatigue in the tunneling layer. The robust trapping/detrapping reversibility up to 10^6 cycles demonstrates the device's reliability under extreme instrumental conditions.

Corresponding changes made in the manuscript

In response to the reviewer's rigorous and constructive feedback, we have carefully re-evaluated the manuscript content. Specifically, we have revised the text to incorporate essential dynamic performance characterizations, acknowledging that pulse-based measurements are critical for thoroughly evaluating any non-volatile memory. Furthermore, to directly address the concerns regarding operational efficiency and reliability, we have added a detailed discussion on the switching speed, energy per operation, and speed-endurance trade-offs. This is supported by new transient pulse measurement data, which verifies that the AHA-RFET achieves fully switched states within a 1 ms pulse, consumes an exceptionally low dynamic energy of ~17.1 pJ per operation, and maintains robust reversibility for 10^6 cycles. These comprehensive characterizations and device benchmarking have been incorporated into **Figure 2c**, **Figure S11** and **Table S1**. We hope these systematic additions significantly improve the technical completeness of the work, and we thank the reviewer again for the valuable recommendation.

"As shown in Figure 2c, pulses as short as 1 ms effectively trigger conductance switching, with current levels scaling significantly following the varying pulse duration." on Page 5.

"Beyond spatial scalability, the AHA-RFET achieves fully P/E switching under rapid pulses with minimal energy consumption, while maintaining robust reversibility and cyclic endurance without significant fatigue behavior of the atomic tunneling layer (**Figure S11**). These metrics are highly competitive among AHA-based charge-trapping devices (**Table S1**)." on Page 5.

8.Finally, the multi-level conductance results are not particularly significant. Commercial flash achieves 3–4 bits per cell in nanometer scale device dimension, while the authors' multi-state operation is shown in large exfoliated $MoTe_2$ channels, where such behavior is predictable.

We thank the reviewer for the critical comment regarding the significance and scaling potential of our multi-level conductance results. We completely agree that demonstrating multi-state operation in large exfoliated channels does not fully address the requirements of high-density integration. To address your concerns, we provide the following clarifications and supplementary data:

(1) Mechanism clarification of Multi-bit Storage

We would like to clarify that the multi-level storage in our AHA-RFET is fundamentally different from commercial 3D NAND flash in terms of physical principles. Commercial flash achieves high bit-per-cell density primarily through complex peripheral circuit control and massive 3D vertical stacking. In contrast, our AHA-RFET achieves high informational density by utilizing the precise charge-trapping dynamics within the AHA stack and the high electrostatic sensitivity of the 2D channel. This enables the device to resolve 64 discrete conductance states within a single, planar footprint.

(2) Evaluation of Multi-level Performance in Sub-micron Devices

To address the reviewer's concerns regarding the correlation between channel dimension and multi-level performance, we provide the following clarification and supplementary data. To directly test whether the multi-state capability survives channel-length scaling, we carried out a targeted scaling experiment on our AHA-RFET. Using Electron Beam Lithography (EBL), we fabricated a scaled-down AHA device with a channel length of 460 nm L_{ch} = 460 nm). The updated **Figure R16** includes optical microscope and AFM images (**Figure R16a**) which confirm that the device has successfully reached sub-micron dimensions. As shown in **Figure R16b**, the transfer curves exhibit a well-defined memory window within a ±15 V gate sweep range, indicating that the fundamental storage characteristics remain unaffected by channel scaling.

Even at this reduced dimension, the device continues to exhibit stable 6-bit (64 discrete levels) storage (**Figure R16c**). By applying precise programming pulses, we achieved discrete threshold voltage shifts and highly reliable conductance states. This robust multi-level performance proves that the AHA stack provides sufficient electrostatic control and trap uniformity to effectively mitigate the stochastic fluctuations often encountered in smaller dimensions. These results demonstrate that the high informational density of our AHA-RFET is not merely a product of large channel areas but is an intrinsic advantage of the AHA-RFET architecture that persists into the sub-micron regime.

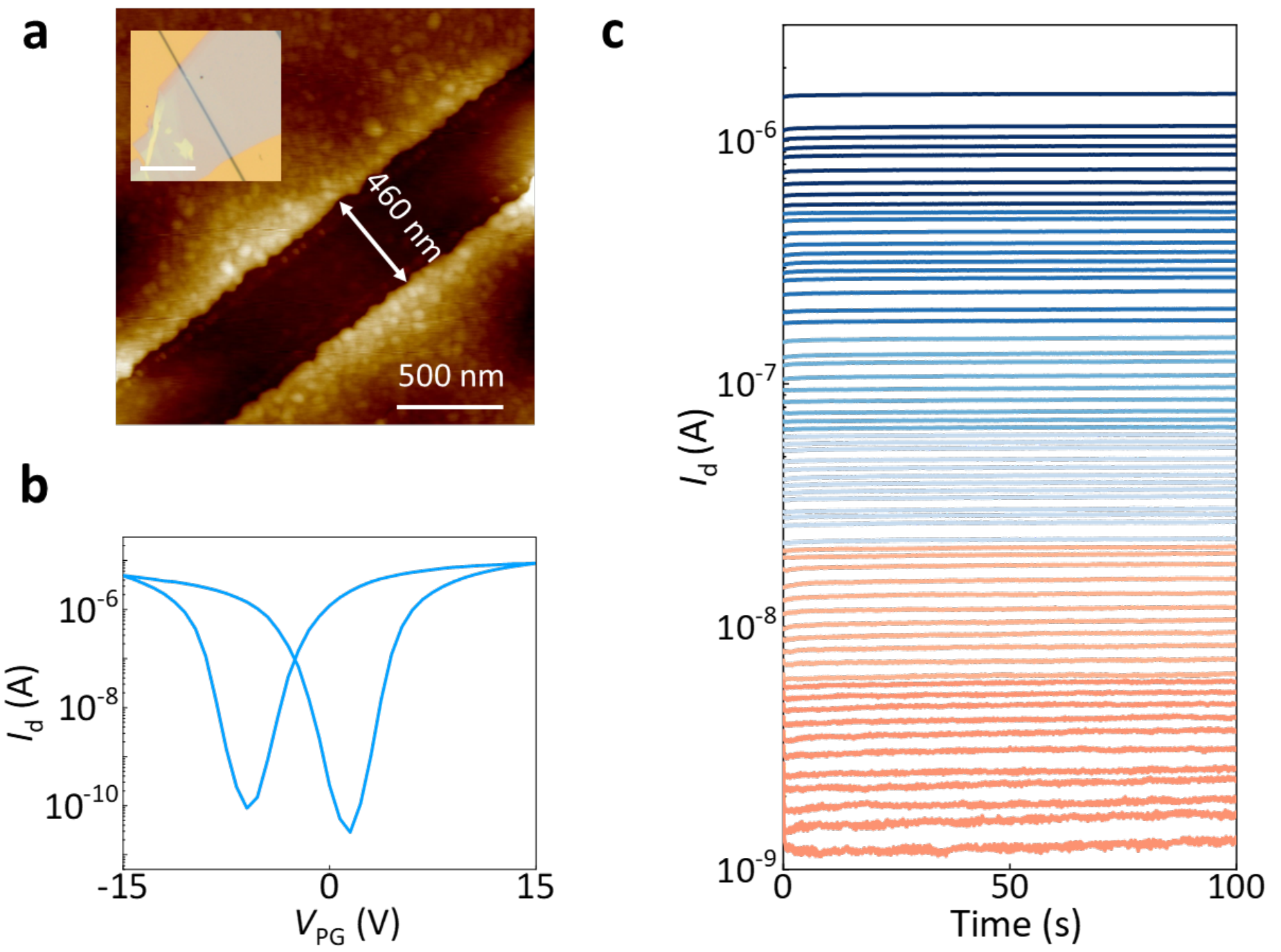

Figure R16. Multi-level conductance characterization of a sub-micron AHA-RFET. (a) Optical microscope (inside) image and AFM topography of the scaled device with a channel length of 460 nm. Scale bar (inside) 10 μm. (b) The transfer characteristics (I_d–V_G) of the sub-micron AHA-RFET. **(c)** Multi-state conductance data demonstrating stable 6-bit (64 discrete levels) storage capability maintained over 100 s. The results confirm that the AHA stack facilitates high-precision charge control and trap uniformity even at the sub-micron scale, mitigating stochastic fluctuations typically encountered in reduced dimensions.

Corresponding changes made in the manuscript:

In the revised manuscript, we have added new sub-micron scaling data (L_{ch} = 460 nm) in **Figure S10**, which confirms that the 6-bit multilevel conductance manipulation is robustly maintained at reduced dimensions. This evidence proves that the high multi-level performance is an intrinsic advantage of the AHA architecture rather than an effect of large channel areas.

"The AHA-RFET's multi-state capability further extends to miniaturized geometries of 460-nm channel length (L_{ch}), for which the reproducible 6-bit multilevel conductance manipulation demonstrate excellent structural scalability (**Figure S10**). This performance at reduced dimensions suggests that the AHA stack provides robust electrostatic control, effectively mitigating the stochastic fluctuations typically encountered in short-channel devices." on Page 5.

Response to Reviewer #4:

Reviewer #4 (Remarks to the Author):

The manuscript presents a scalable reconfigurable field-effect transistor (RFET) platform based on an oxide–van der Waals quasi-floating-gate architecture and top-gate engineering, which enable nonvolatile polarity switching and multi-state programmability. The integration of an $Al_2O_3/HfO_2/Al_2O_3$ (AHA) dielectric stack with a dual-gate design demonstrates reliable non-volatile electrical manipulation, including high endurance (>10^6 cycles), long retention (>10^4 s), and well-balanced N/P operation. The work further showcases solid experimental demonstration on the reconfigurable logic gates and compact ternary content-addressable memory (TCAM), highlighting its potential for logic in-memory computing. The study is well-structured, experimentally thorough, and supported by TCAD simulations and surface potential analysis. Overall, this manuscript presents an outstanding advancement in reconfigurable logic device design, integrating novel material engineering, robust device physics, and system-level prototyping with a clear path to integration. It addresses multiple key issues that have hampered prior efforts, namely, symmetric polarity switching, retention, endurance, and CMOS compatibility. I support publication in Nature Communications, pending minor revisions to clarify and complete the following points.

We are sincerely grateful to Reviewer #4 for the highly positive assessment of our AHA-RFET and for the insightful suggestions that have significantly enhanced the depth of this work. To address the reviewer's points and further demonstrate the robustness of our technology, we have performed comprehensive supplementary characterizations, which are summarized below:

(1) Universality of the Device Design: We fabricated devices using an alternative $SiO_2\backslash HfO_2\backslash SiO_2$ (SHS) dielectric stack and extended the AHA-RFET protocol to various 2D channel materials beyond $MoTe_2$, confirming that our quasi-floating-gate architecture is a universal design (**Figure R17, Figure R18**).

(2) In-depth Noise Analysis: We conducted low-frequency noise (LFN) measurements (**Figure R19**), proving that the noise in AHA-RFETs primarily originates from the charge trapping and de-trapping dynamics within the dielectric stack.

(3) Systematic Structural Optimization: We explored AHA stacks with various thickness configurations, observing consistent and stable non-volatile reconfigurable behavior with predictable performance trends across all batches (**Figure R20**).

(4) Scalable Integration Path: We demonstrated the integration feasibility of our AHA-RFET with CVD-grown 2D materials, showcasing excellent uniformity in FET arrays and establishing a clear path for large-scale manufacturing (**Figure R21**).

1.Regarding the device structure, the manuscript successfully demonstrated the functionality of the AHA stack with a specific thickness. To better establish the design's generality, could the authors comment on whether such a multiple dielectric stacking configuration is a universal design? Are there more material choices for this triple-layer dielectric stack?

We thank the reviewer for this constructive suggestion. Establishing the versatility of this dielectric-stack-based RFET structure is pivotal for validating the fundamental physics of the quasi-floating-gate architecture and expanding the design landscape for diverse material systems. To address this, we performed supplementary experiments involving alternative dielectric and channel materials:

(1) Generality of the Dielectric Configuration

The quasi-floating-gate mechanism is not restricted to the specific $Al_2O_3/HfO_2/Al_2O_3$ (AHA) combination but is fundamentally rooted in the stack engineering of energy band offsets among the tunneling, trapping, and blocking layers. To verify this, we fabricated alternative devices employing a SiO_2\$HfO_2$\$SiO_2$ (SHS) configuration (**Figure R17a**). The transfer characteristics of the device (**Figure R17b**) show that, despite the change in dielectric materials, we observed consistent non-volatile charge-trapping dynamics and reliable polarity switching. This confirms that our triple-layer stacking is a universal design rule; as long as the dielectric synergy facilitates controlled charge injection and retention, the quasi-floating-gate effect can be successfully implemented across various oxide systems.

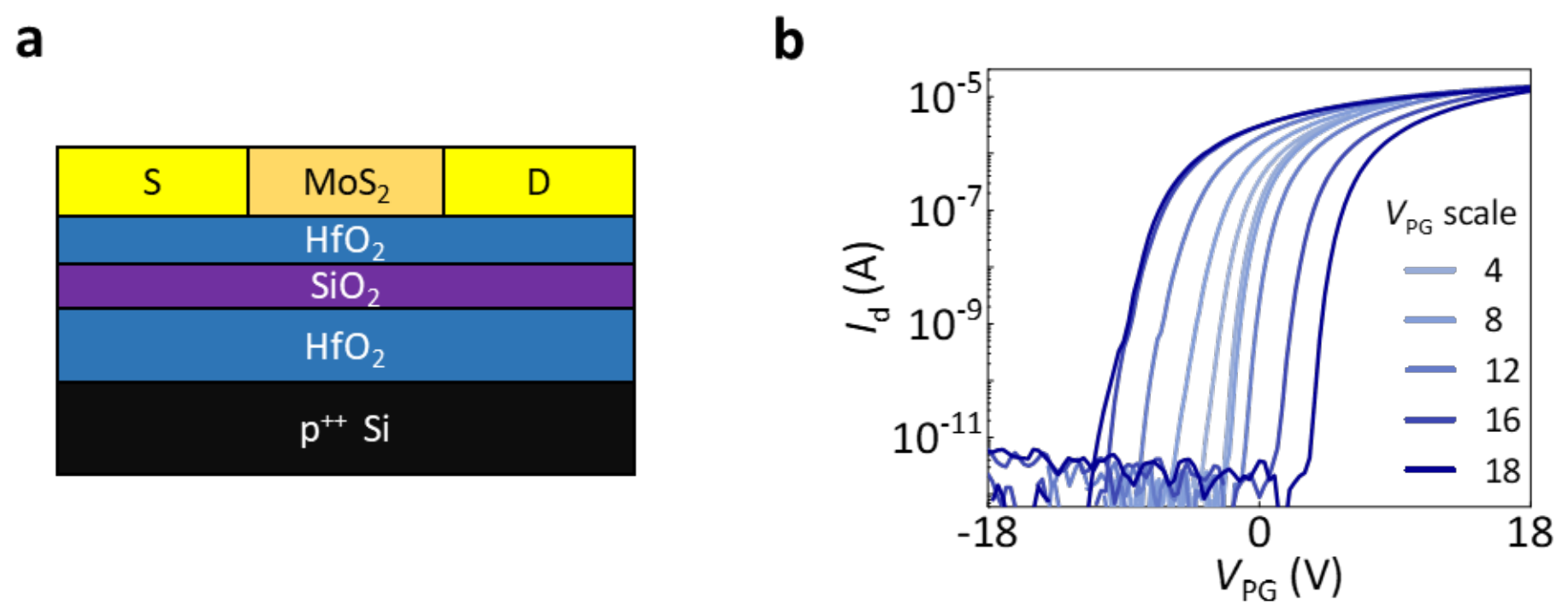

Figure R17. Generality of the quasi-floating-gate mechanism with an alternative SHS dielectric configuration. a. Schematic illustration of the device architecture featuring a $SiO_2/HfO_2/SiO_2$ (SHS) dielectric stack integrated with the 2D channel. **b.** Transfer characteristics (I_d–V_{PG}) of $SiO_2/HfO_2/SiO_2$ (SHS) dielectric FET demonstrating a distinct and reliable non-volatile memory window.

(2) Generality of the Channel Materials

We would like to point out that the AHA-RFET is inherently material-agnostic and compatible with various 2D semiconductors. Throughout our study, we extensively characterized several materials, including WSe_2- and $ReSe_2$-based AHA-FETs, to verify this versatility (**Figure R18**). As expected, the consistent emergence of reliable

polarity switching across these distinct material systems underscores the robustness and universal nature of our design.

Comparison of experimental results, we observed that $MoTe_2$ provides a more balanced performance profile than WSe_2—specifically superior current matching between the P- and N-branches. Since high-performance logic gate demonstrations require symmetric driving capabilities, $MoTe_2$-based results are employed in the main text. Meanwhile, the comprehensive data for WSe_2 and $ReSe_2$ have been allocated to the Supplementary Information to support our conclusion regarding the universal role of the AHA stack.

These results collectively establish our architecture as a robust design that can be generalized to a wide range of dielectric-semiconductor combinations.

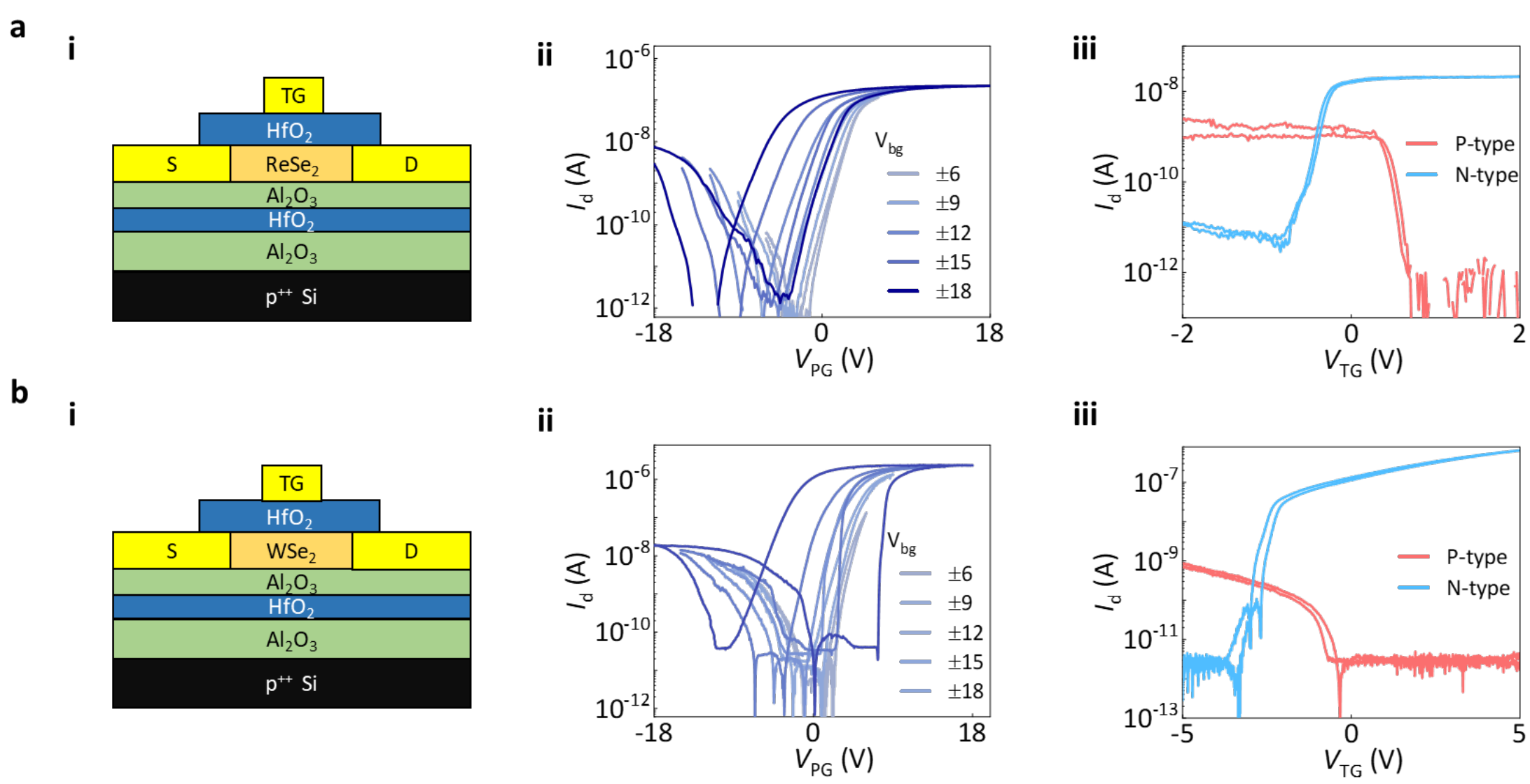

Figure R18. Universal applicability of the AHA architecture across diverse 2D semiconductor channels. Characterization of AHA-FETs utilizing $ReSe_2$ (**a**) and WSe_2 (**b**) as channel materials. (**i**) Device schematics illustrating the integration of the AHA dielectric stack with different semiconductors. (**ii**) Transfer characteristics (I_d–V_{PG}) under varying programming voltages scale and (**iii**) dual-polarity transport under top gate, demonstrations showing well-defined P-type and N-type branches.

Corresponding changes made in the manuscript

To demonstrate the generality of our design, we have incorporated supplementary data for alternative dielectric stacks ($SiO_2/HfO_2/SiO_2$) and diverse 2D channels (WSe_2 and $ReSe_2$) into **Figure S13** and **Figure S14**. These results confirm that the quasi-floating-gate mechanism is a universal design rule effective across various material combinations. The manuscript has been revised to emphasize the material-agnostic nature of the AHA architecture for achieving reliable polarity switching.

"Notably, this synergistic polarity modulation extends beyond $MoTe_2$ to WSe_2 and $ReSe_2$, establishing the AHA architecture as a universal strategy for reconfigurable devices (**Figure S13**). Given the better current matching behavior of P- and N-branches, $MoTe_2$ devices are employed for the following logic-circuit demonstrations. Beyond

material versatility, the quasi-floating-gate mechanism is independent of the specific AHA combination. By employing an alternative $SiO_2/HfO_2/SiO_2$ (SHS) stack, we observed similarly consistent non-volatile dynamics and reliable polarity switching (**Figure S14**). These results establish the stack-engineered quasi-floating-gate architecture as a universal and robust strategy for non-volatile polarity modulation in diverse 2D electronic devices." on Page 6.

2.For physical mechanisms, the authors attributed the nonvolatile polarity switching to charge trapping at oxygen vacancies within the HfO_2 layer, as determined through device structural simulation. Is it possible to characterize this phenomenon experimentally? A more direct investigation of the carrier trapping dynamics would strengthen the discussion.

We thank the reviewer for this insightful suggestion. To provide direct experimental evidence of the carrier trapping dynamics and further strengthen our mechanistic discussion, we have performed low-frequency noise (LFN) characterizations on the AHA-RFETs. The noise behavior of our devices provides critical insights into the origin of the non-volatile polarity switching. As shown in the updated **Figure R19**, the results are summarized as follows:

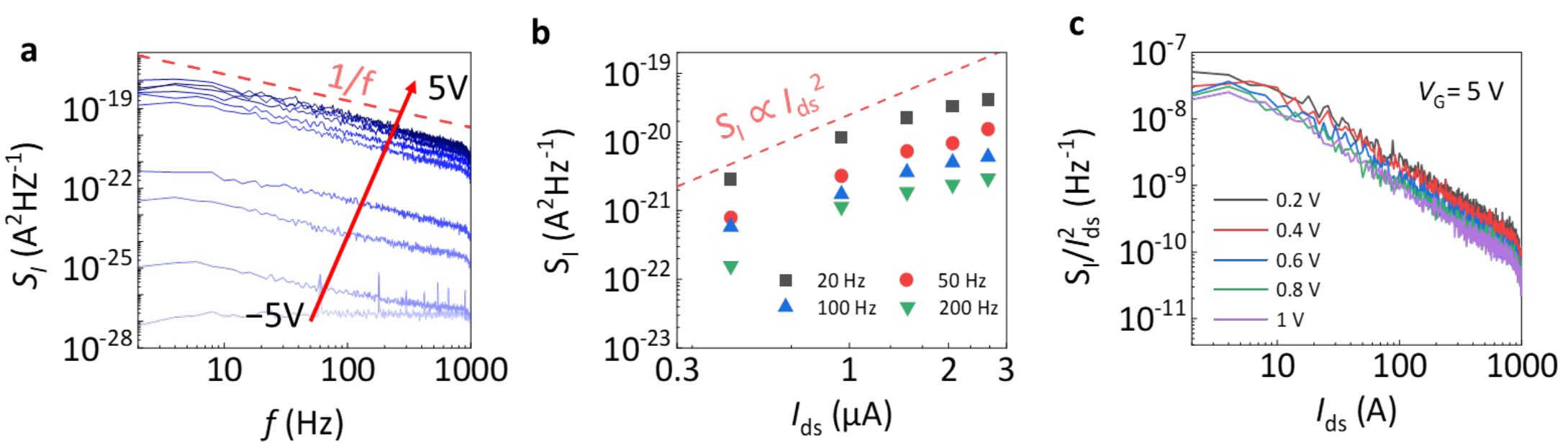

Figure R19 Low-frequency noise analysis of the AHA-RFET. a. Noise power spectral density (S_I) as a function of frequency (f) under various gate voltages, exhibiting a clear $1/f$ dependence. **b.** S_I as a function of drain current (I_{ds}) at different frequencies; the dashed line represents the $S_I \propto I_{ds}^2$ relationship, characteristic of resistance fluctuations. **c.** Normalized noise (S_I/I_{ds}^2) versus I_{ds} at various V_{ds} values. The overlapping curves indicate that the LFN originates from the conductive channel rather than contact barriers, while the significant decrease with increasing current points to the dominance of surface-trapping events at the dielectric/channel interface.

Through measuring the noise power spectral density (S_I) as a function of frequency under various gate voltages, we observed a clear $1/f$ dependence (**Figure R19a**), indicating the presence of typical flicker noise behavior in our devices. Furthermore, analyzing the relationship between S_I and the drain current (I_{ds}) across different frequencies reveals that the extracted data strictly follows the S_I/I_{ds}^2 scaling (**Figure R19b**). This demonstrates that the device noise is fundamentally governed by carrier resistance fluctuations. Building on this, to explicitly isolate the noise source, we evaluated the normalized noise (S_I/I_{ds}^2) under varying drain-source voltages (V_{ds}). As

depicted in **Figure R19c**, the normalized noise curves at different V_{ds} exhibit perfect overlapping. This crucial bias-independence confirms that the noise fundamentally originates from the conductive channel rather than the contact barriers, thereby providing definitive experimental evidence that the non-volatile polarity switching is dominated by trapping/detrapping events near the channel.

By demonstrating that the noise originates from a uniform trap distribution surrounding the channel, we provide robust experimental validation for the non-volatile reconfigurable mechanism of the AHA-RFET. We have incorporated these characterizations and corresponding discussions into the revised manuscript.

Corresponding changes made in the manuscript

To provide direct experimental evidence of the carrier trapping dynamics, we have incorporated low-frequency noise (LFN) characterizations into the revised manuscript and Supplementary Information as **Figure S19**. The observed 1/f noise behavior and the $S_I \propto I_{ds}^2$ scaling, combined with the bias-independent normalized noise curves, definitively confirm that the non-volatile polarity switching originates from trapping/detrapping events at the dielectric/channel interface.

"To probe the microscopic origin of nonvolatile reconfigurability and interface quality, low-frequency noise (LFN) characterizations are further performed (**Figure S19**). The measured noise power spectral density exhibits a distinct $1/f$ dependence, and the normalized noise (S_I/I_{ds}^2) remains nearly independent of the drain bias, confirming that the dynamic charge disturbances in the devices are governed by trapping/detrapping at the AHA stack rather than the source-drain contact or carrier mobility variations. This validates the high-quality, low-defect nature of the heterointerface and the precise electrostatic control afforded by the AHA stack." on Page 7.

3.Considering that carrier trapping and tunneling are critical for constructing the nonvolatile polarity switching behavior, the material thickness effect of such an AHA stack on the electrical characteristics should be clarified. Does this non-volatile feature strongly rely on a particular thickness? A systematic benchmark in terms of the device parameters, such as dielectric layer, thickness, retention behaviors, and multiple-state features, should be provided.

We thank the reviewer for this insightful question regarding the thickness optimization of the AHA dielectric stack. Establishing the thickness-dependent characteristics is crucial for validating the robustness of the charge-trapping mechanism. In response, we conducted a systematic study by fabricating several batches of AHA-RFETs with varying layer thicknesses to evaluate their electrical performance (**Figure R20**). Our results demonstrate that stable non-volatile polarity switching is consistently observed across all thickness configurations, confirming that the trapping dynamics are not restricted to a specific dimension. The detailed findings are summarized as follows:

(1) **Operating Voltage Dependence on Blocking Layer Thickness**: As shown in **Figure R20a**, we first evaluated the operating voltage based on the programming gate voltage required to open a distinct memory window for devices with different blocking

layer thicknesses (20, 24, and 30 nm). The memory window is quantified as the ratio of the threshold voltage shift to the sweeping range, defined by the formula:

$$\text{Memory Window (\%)} = \frac{\Delta V_{th}}{V_{PG,max} - V_{PG,min}} \times 100\%$$

The variation of the memory window with operating voltage shows that a thinner blocking layer requires a lower voltage to achieve the same proportional memory window. Our systematic characterization confirms that both the required operating voltage and the breakdown threshold increase in correlation with the thickness of the blocking layer. Specifically, for the 20 nm blocking layer, the memory window opens at ±8 V, and the device approaches its breakdown limit at ±12 V. For the 24 nm blocking layer, the window opens at ±12 V, with the breakdown limit near ±15 V. Finally, for the 30 nm blocking layer, the memory window opens at ±15 V, and it nears the breakdown limit at ±21 V.

(2) **Optimization of Response Speed via Tunneling Layer Thickness**: To evaluate the programming speed, we applied a −10 V programming pulse with varying durations (from 10 μs to 1 s) and monitored the transient I_{ds} response. As presented in **Figure R20b**, the variation of drain current response with programming pulse width demonstrates that a thinner tunneling layer significantly accelerates the programming response speed. This is further detailed in the transient I_{ds} responses (**Figure R20d**), which reveal that the 6 nm tunneling layer enables fast switching within 50 μs (**Figure R20d, i**), whereas the 8 nm version requires 100 μs (**Figure R20d, ii**). For the 10 nm version, the response remains limited, requiring significantly longer pulses (up to 500 μs) to achieve a similar current response (**Figure R20d, iii**).

It should be noted that the choice of a 10 V programming voltage is primarily dictated by the hardware constraints of our Keysight B1500A system. Specifically, to capture these microsecond-scale dynamics, we utilized the Fast Measurement Unit (B1530A), which enables ultra-fast pulses down to 50 ns but is strictly restricted to a maximum output of ±10 V. While the High-Resolution Source/Monitor Unit (B1517A) can provide higher voltages, its minimum pulse width is limited to 1 ms, which is insufficient for characterizing the switching behavior in the microsecond regime.

(3) **Retention Capability Enhancement through Tunneling Layer Engineering**: To assess non-volatile reliability, we continuously monitored the on-state and off-state currents over a prolonged period (8000 s) after removing the programming voltage. As shown in **Figure R20c**, the variation of current decay over time indicates that optimizing the tunneling layer thickness effectively suppresses current loss to enhance data retention. The current decay is defined as the ratio of the current change to the initial current, calculated by the formula:

$$\text{Current Decay (\%)} = \frac{I_0 - I(t)}{I_0} \times 100\% = \frac{\Delta I}{I_0} \times 100\%$$

The detailed retention tests (**Figure R20e**) demonstrate that for the 6 nm tunneling layer, the high-state current drops noticeably (by nearly an order of magnitude) within 8,000 s (**Figure R20e, i**). In contrast, for both the 8 nm and 10 nm layers (**Figure R20e, ii and iii**), the high-state current remains nearly constant over the same duration, demonstrating robust non-volatile characteristics.

Based on these systematic comparisons, the 8/8/24 nm configuration was selected as it provides the optimal balance between high-speed operation, a manageable programming voltage, and excellent long-term retention. We have incorporated these experimental justifications into the revised manuscript as **Figure S3**.

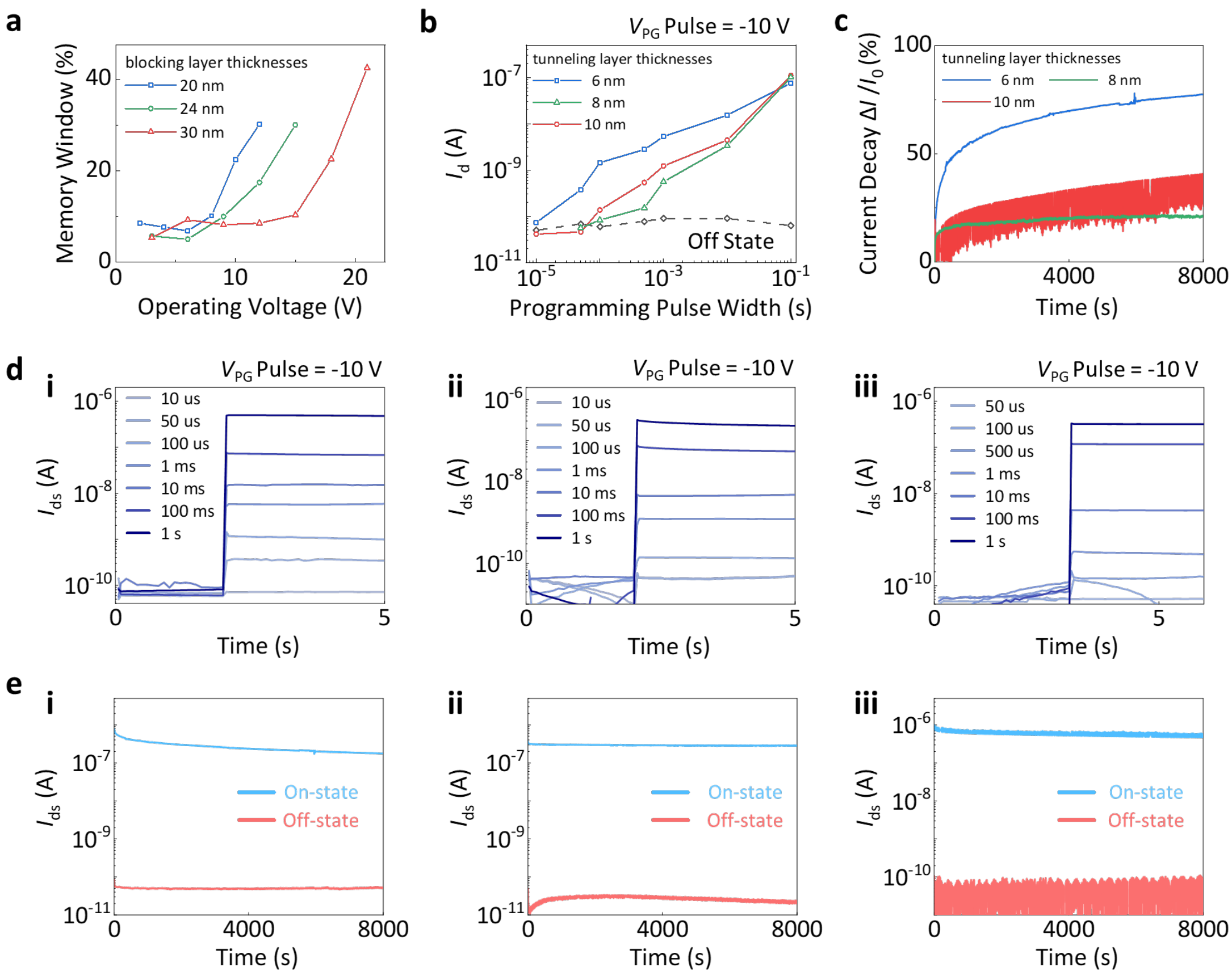

Figure R20. Systematic optimization and justification of the AHA dielectric stack thicknesses. a. The variation of the memory window with operating voltage under different blocking layer thicknesses, showed that a thinner blocking layer requires a lower voltage to achieve a same proportional memory window. **b.** The variation of drain current response with programming pulse width under different tunneling layer thicknesses, demonstrated that a thinner tunneling layer significantly accelerates the programming response speed. **c.** The variation of current decay over time under different tunneling layer thicknesses, indicated that optimizing the tunneling layer thickness effectively suppresses current loss to enhance data retention. **d.** Transient I_d response to pulses of varying widths, reveals that the 6 nm tunneling layer enables fast switching within 50 µs, whereas the 10 nm version requires significantly longer pulses

(up to 500 μs) to achieve a similar current response. **e.** Retention tests of the on-state and off-state over 8,000 s, demonstrate the 6 nm tunneling layer leads to noticeable current decay. **i-iii.** Multi-configuration comparisons across three specific tunneling layer thickness recipes: 6 nm (**i**), 8 nm (**ii**), and 10 nm (**iii**).

Corresponding changes made in the manuscript

In response to the reviewer's insightful comments, we have carefully re-evaluated the manuscript content and the Supplementary Information. Specifically, we have incorporated a systematic optimization study of the AHA dielectric stack by characterizing various thickness configurations. We have described the comprehensive consideration of the relationship between thickness and various performance metrics to justify our design selection. These experimental justifications and the comprehensive performance benchmarks have been added to the revised manuscript as **Figure S3**. We hope these changes can clarify our technical route and improve the overall accuracy of the work, and we thank the reviewer again for the valuable recommendation.

"This specific 8/8/24 nm AHA configuration is optimized among various thickness design to precisely balance the trade-off among several critical parameters, such as operating voltage, programming speed, and non-volatile charge retention (**Figure S3**). Such an optimized dielectric engineering ensures both robust reconfiguration and long-term stability for the designed structure." on Page 4.

4. The manuscript acknowledges the use of mechanically exfoliated $MoTe_2$ as a limitation. Have the authors evaluated the feasibility of CVD-grown or wafer-scale vdW materials for integration? Scalable channels would help contextualize the near-term applicability.

We thank the reviewer for this insightful comment. We fully agree that transitioning from mechanical exfoliation to wafer-scale fabrication is a critical step for the near-term applicability of vdW electronics. To address this, we have performed supplementary experiments to evaluate the integration feasibility of our fabrication flow using large-area synthesized 2D materials.

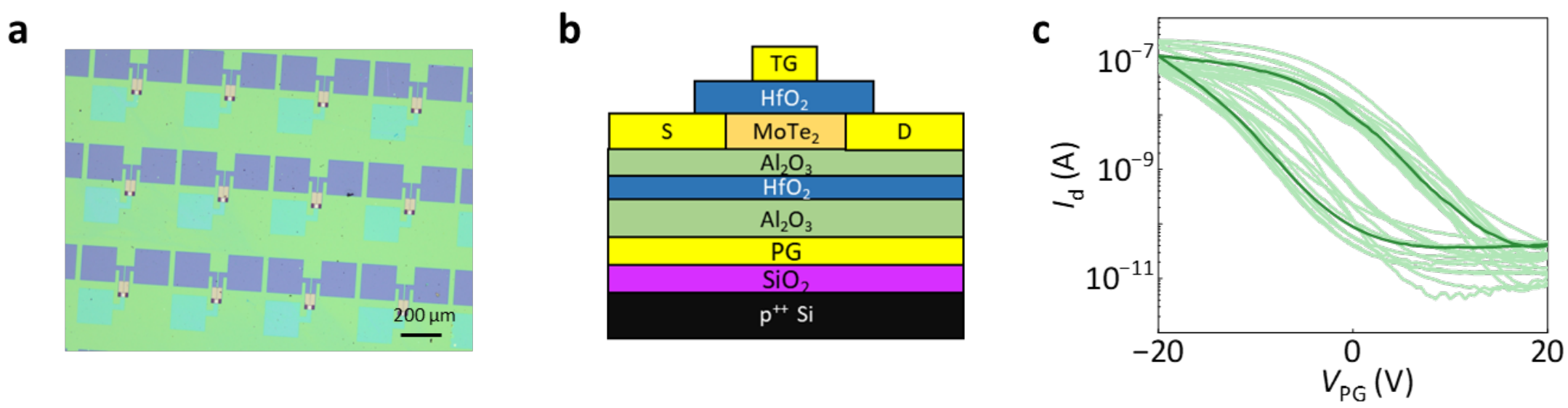

Figure R21. Evaluation of integration potential and uniformity using large-area synthesized $MoTe_2$. a. Optical microscope image of an AHA-RFET array fabricated using CVD-grown $MoTe_2$, with a scale bar of 200 um. **b.** Schematic illustration of the device architecture. **c.** Transfer characteristics (I_d–V_{PG}) of multiple devices within the

array, demonstrating high fabrication yield and excellent consistency across the large-area synthesized 2D Material.

As shown in **Figure R21** (added to the revised manuscript), we have successfully fabricated regular device arrays using CVD-grown 2D materials. This demonstration proves that our entire integration protocol—including high-resolution lithography, contact engineering, and top-gate dielectric deposition—is fully compatible with wafer-scale synthesized materials. While the primary focus of this work is the fundamental physics of the AHA-RFET architecture, the high uniformity and yield observed in these CVD-based arrays establish a clear technical path for scaling up our quasi-floating-gate design to high-density, wafer-level systems in the future. We have added these results and corresponding discussions to the revised manuscript to better contextualize the scalability of our approach.

Corresponding changes made in the manuscript

To evaluate scalability and near-term applicability, we have incorporated supplementary data for CVD-grown 2D materials $MoTe_2$ into **Figure S24**. These results demonstrate regular device arrays with high yield and uniform performance, proving our fabrication protocol—including lithography and dielectric deposition—is fully compatible with wafer-scale synthesis. The revised manuscript now includes a discussion on the technical path toward high-density, wafer-level integration.

"It is worth mentioning that, although the precise carrier modulation and synthetic approaches of ambipolar materials currently require further optimization, the AHA-stack structure establishes a robust and scalable manufacturing route for reconfigurable electronic devices (**Figure S24**). Thus, high-quality exfoliated $MoTe_2$ was utilized to achieve optimal current matching and rigorously evaluate the intrinsic physics of the AHA-RFET device." on Page 9.
